# Calcium-dependent cytosolic phospholipase A_2_ activation is implicated in neuroinflammation and oxidative stress associated with ApoE4

**DOI:** 10.1186/s13024-022-00549-5

**Published:** 2022-06-15

**Authors:** Shaowei Wang, Boyang Li, Victoria Solomon, Alfred Fonteh, Stanley I. Rapoport, David A. Bennett, Zoe Arvanitakis, Helena C. Chui, Patrick M. Sullivan, Hussein N. Yassine

**Affiliations:** 1 Keck School of Medicine, University of Southern California, Los Angeles, CA USA; 2 Huntington Medical Research Institutes, Pasadena, CA USA; 3 National Institute on Alcohol Abuse and Alcoholism, Bethesda, MD USA; 4 Rush Alzheimer’s Disease Center, Rush University Medical Center, Chicago, IL USA; 5 Department of Medicine, Duke University Medical Center, Durham Veterans Health Administration Medical Center’s Geriatric Research, Education and Clinical Center, Durham, NC USA

**Keywords:** cPLA2, ApoE4, Alzheimer’s disease, p38 MAPK, Neuroinflammation, Oxidative stress

## Abstract

**Background:**

Apolipoprotein E4 (*APOE4*) is associated with a greater response to neuroinflammation and the risk of developing late-onset Alzheimer’s disease (AD), but the mechanisms for this association are not clear. The activation of calcium-dependent cytosolic phospholipase A_2_ (cPLA2) is involved in inflammatory signaling and is elevated within the plaques of AD brains. The relation between *APOE4* genotype and cPLA2 activity is not known.

**Methods:**

Mouse primary astrocytes, mouse and human brain samples differing by *APOE* genotypes were collected for measuring cPLA2 expression, phosphorylation, and activity in relation to measures of inflammation and oxidative stress.

**Results:**

Greater cPLA2 phosphorylation, cPLA2 activity and leukotriene B4 (LTB4) levels were identified in ApoE4 compared to ApoE3 in primary astrocytes, brains of ApoE-targeted replacement (ApoE-TR) mice, and in human brain homogenates from the inferior frontal cortex of persons with AD dementia carrying *APOE3/4* compared to *APOE3/3*. Higher phosphorylated p38 MAPK but not ERK1/2 was found in ApoE4 primary astrocytes and mouse brains than that in ApoE3. Greater cPLA2 translocation to cytosol was observed in human postmortem frontal cortical synaptosomes with recombinant ApoE4 than ApoE3 ex vivo. In ApoE4 astrocytes, the greater levels of LTB4, reactive oxygen species (ROS), and inducible nitric oxide synthase (iNOS) were reduced after cPLA2 inhibition.

**Conclusions:**

Our findings implicate greater activation of cPLA2 signaling system with *APOE4*, which could represent a potential drug target for mitigating the increased neuroinflammation with *APOE4* and AD.

**Supplementary Information:**

The online version contains supplementary material available at 10.1186/s13024-022-00549-5.

## Background

The enzyme phospholipase A2 (PLA2) catalyzes the hydrolysis of the stereospecifically numbered (*sn*-2) ester bond of substrate phospholipids in the cell membrane to produce a free fatty acid and a lysophospholipid [1]. Calcium-independent PLA2 (iPLA2) has a greater affinity for releasing docosahexaenoic acid (DHA, 22:6 n-3), which acts as a signaling molecule during neurotransmission and as the precursor of anti-inflammatory and antioxidant resolvins [2, 3]. Calcium-dependent cytosolic phospholipase A2 (cPLA2) releases arachidonic acid (AA, 20:4 n-6), which plays important functions in storing energy, as a second messenger in neurotransmission, and as the precursor of eicosanoids [4, 5]. Free AA can be oxidized by cyclooxygenase (COX) or lipoxygenase (LOX) to produce prostaglandins or leukotrienes, which are potent mediators of inflammation [1, 6]. In astrocytes, cPLA2 interacts with mitochondrial antiviral-signaling protein (MAVS) to boost nuclear factor kappa-light-chain-enhancer of activated B cell (NF-kB)-driven inflammatory responses [7]. In microglia, cPLA2 and AA metabolic pathways contribute to reactive oxygen species (ROS) and nitric oxide (NO) production during cell activation [8]. cPLA2 activity depends on its phosphorylation, regulated by mitogen-activated protein kinase (MAPK) pathways [9, 10].

A lower amount of Aβ oligomers and the absence of glial activation markers in both astrocytes and microglia distinguish the brains of individuals with greater brain Aβ plaques and tangles but resilience to AD dementia from those with dementia [11]. cPLA2 activation is one of the pathways that activates microglia and astrocytes in the brain. The cPLA2 gene, protein levels, and phosphorylated form are increased around AD brains’ plaques compared to healthy controls [12–14]. Increased activation of cPLA2 is observed in the hippocampus of human amyloid precursor protein (hAPP) transgenic mice [14]. The activation of cPLA2 by Aβ oligomers contributes to dysregulation of fatty acid metabolism and promotes neurodegeneration [15, 16]. Overexpression of p25 (Protein 25, a cyclin-dependent kinase 5 activator) in neurons increases the expression of cPLA2, leading to lysophosphatidylcholine (LPC) secretion and the activation of astrocytes and production of proinflammatory cytokines [17]. Conversely, cPLA2 deficiency in AD mouse models ameliorates the memory impairment and hyperactivated glial cells observed in AD mouse models [14, 18]. Knocking out cPLA2 in microglia decreases lipopolysaccharide (LPS) induced oxidative stress and inflammatory response [8].

Carrying the *APOE4* allele is the strongest genetic risk factor for late-onset AD. The ApoE4 protein seems to have proinflammatory and/or reduced anti-inflammatory functions, which could exacerbate AD pathology. This ApoE4 effect on inflammation was clearly demonstrated in the Framingham cohort, where participants with *APOE4* and elevated plasma C-reactive protein (CRP) levels had a greater risk of developing late-onset of AD than age and sex-matched *APOE2* and *APOE3* carriers [19]. In the brains of participants with AD, *APOE4* is associated with greater levels of lipid peroxidation, eicosanoids, and oxidative stresses markers [20], but the mechanisms for these observations are not clear. Here, we hypothesized that ApoE4 activates cPLA2 to enhance AA release and eicosanoid levels, leading to an enhanced inflammatory and oxidative stress response. Accordingly, we examined cPLA2 expression and activation in mouse primary astrocytes and in mouse and human brain samples that differed by *APOE* genotype, and determined the cellular effects of cPLA2 inhibition on measurements of neuroinflammation and oxidative stress.

## Results

### cPLA2 and phosphorylated cPLA2 are increased in ApoE4 mouse primary astrocytes

**Fig. 1:**
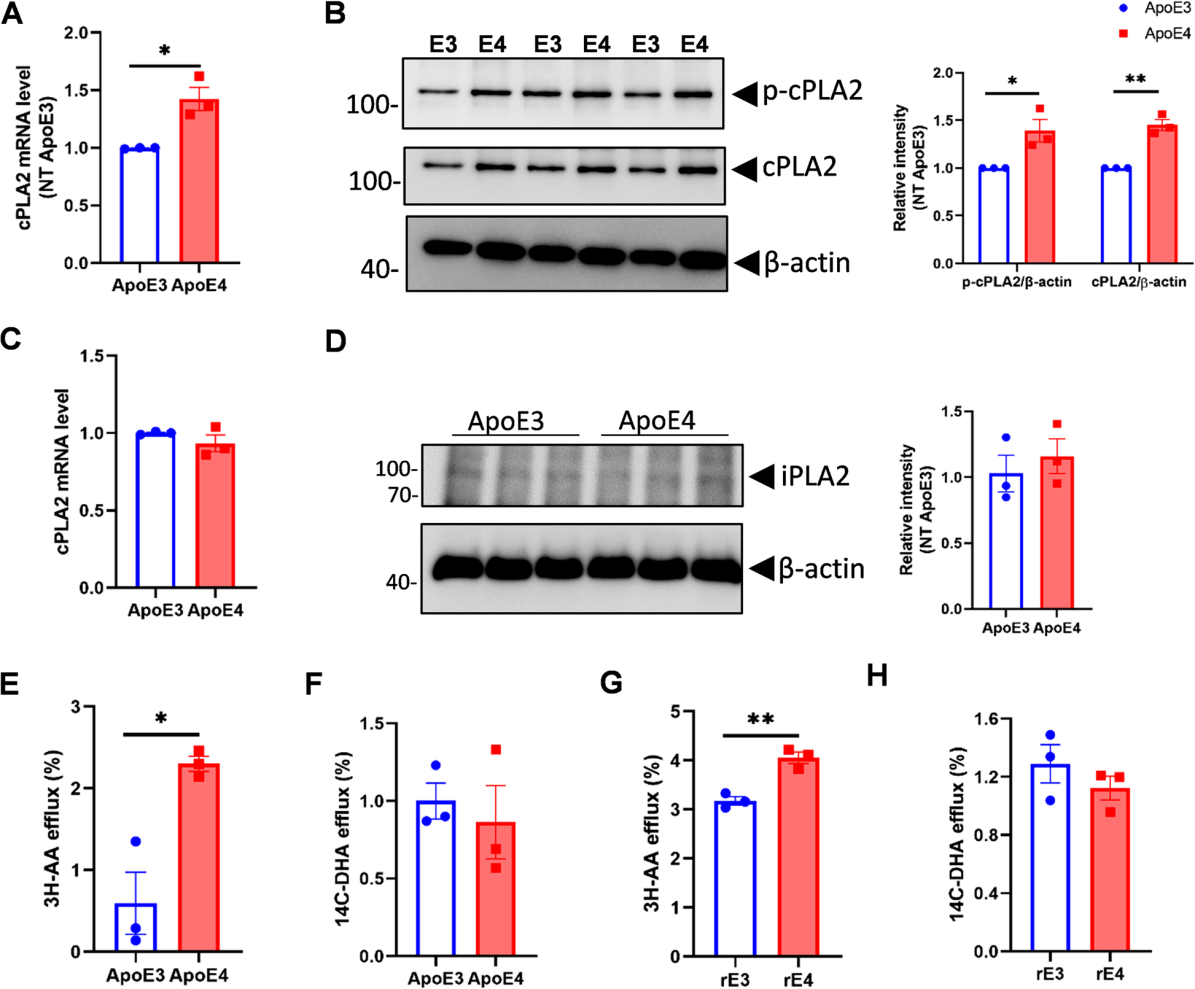
ApoE4 increases cPLA2 but not iPLA2 expression in mouse primary astrocytes. Primary astrocytes were cultured from the cortex of P1-P4 mouse pups. **A** cPLA2 mRNA levels in primary astrocytes from ApoE3 and ApoE4-TR mice were measured by qPCR. **B** cPLA2, and phosphorylated cPLA2 (p-cPLA2) protein levels in primary astrocytes from ApoE3 and ApoE4-TR mice were detected by Western blot. **C** iPLA2 mRNA levels in primary astrocytes from ApoE3 and ApoE4-TR mice. **D** iPLA2 protein levels in primary astrocyte cultures from ApoE3 and ApoE4-TR mice was detected by Western blot. (*n* = 3 for each genotype for A-D). **E**, **F** Primary astrocytes from ApoE3 and ApoE4-TR mice were incubated with ^3^H-labelled AA (E) or ^14^C-labeled DHA (F) for 24 h, followed by induction by 100 nM ATP for 15 min. The efflux of ^3^H-AA (E) and ^14^C-DHA (F) from cells to media was measured by scintillation counting. **G, H** Primary astrocytes from C57BL/6 wild type mice were labeled with ^3^H-AA (G) or ^14^C-DHA (H) for 24 h, and then treated with rE3 or rE4 (0.2 μM) for 24 hours, followed by induction with 100 nM ATP for 15 min. ^3^H-AA (G) and ^14^C-DHA (H) efflux were measured by scintillation counting (*n* = 3 for E-H). Data are represented as mean ± SEM and analyzed by Student’s t-test (two-tailed). *p < 0.05, **p < 0.01. *rE3*: recombinant ApoE3, *rE4*: recombinant ApoE4

We previously found that DHA/AA ratio in cerebrospinal fluid (CSF) is lower in *APOE4/E4* carriers compared to *APOE3/E3* carriers [21, 22]. Since astrocytic cPLA2 and iPLA2 enzymes are important determinants of brain AA and DHA metabolism [2, 23], these enzymes’ expression and activity were first examined in primary astrocytes from ApoE-TR mice. First, total and phosphorylated cPLA2 antibodies were validated using cPLA2 siRNA or ATP treatment in astrocytes, respectively (Fig. S1A and B). ApoE4 astrocytes had greater mRNA and protein levels of cPLA2 and phosphorylated cPLA2 compared with ApoE3 astrocytes (Fig. 1A, B). In contrast, iPLA2 mRNA and protein levels did not differ between ApoE4 and ApoE3 primary astrocytes (Fig. 1C, D). These measures were also significantly greater in ApoE4 immortalized astrocytes compared to ApoE3 (Fig. S2A and B). No differences were found in phosphorylated and total cPLA2 level between ApoE3 and ApoE4 primary microglial cells from mice (Fig. S2C). To identify cellular cPLA2 localization, cytosolic and membrane fractions were obtained from primary ApoE astrocytes. As expected, the majority of cPLA2 was present in the cytosol (Fig. S2). To further explore the activities of cPLA2 and iPLA2, the efflux of ^3^H-AA or ^14^C-DHA from ApoE3 and ApoE4 primary astrocyte cells to media with or without ATP stimulation for 15 min was examined. ^3^H-AA efflux was significantly greater in stimulated ApoE4 compared to ApoE3 primary astrocytes (Fig. 1E), whereas ^14^C-DHA efflux showed no difference between ApoE4 and ApoE3 (Fig. 1F). To confirm the ApoE protein’s effect, cultured primary astrocytes from C57BL/6 mice were labeled with ^3^H-AA or ^14^C-DHA and then treated with 0.2 μM recombinant ApoE3 (rE3) or recombinant ApoE4 (rE4) proteins for 24 h under similar conditions to primary astrocytes cultured from ApoE-TR mice. ^3^H-AA efflux was greater after rE4 than rE3 treatment (Fig. 1G), whereas DHA efflux did not differ between rE4 and rE3 treatments (Fig. 1H). Taken together, these results confirmed that cPLA2 expression and activity were greater in ApoE4 compared to ApoE3 astrocytes.

### Phosphorylated cPLA2 and cPLA2 activity are increased in *APOE4* mouse brains

**Fig. 2:**
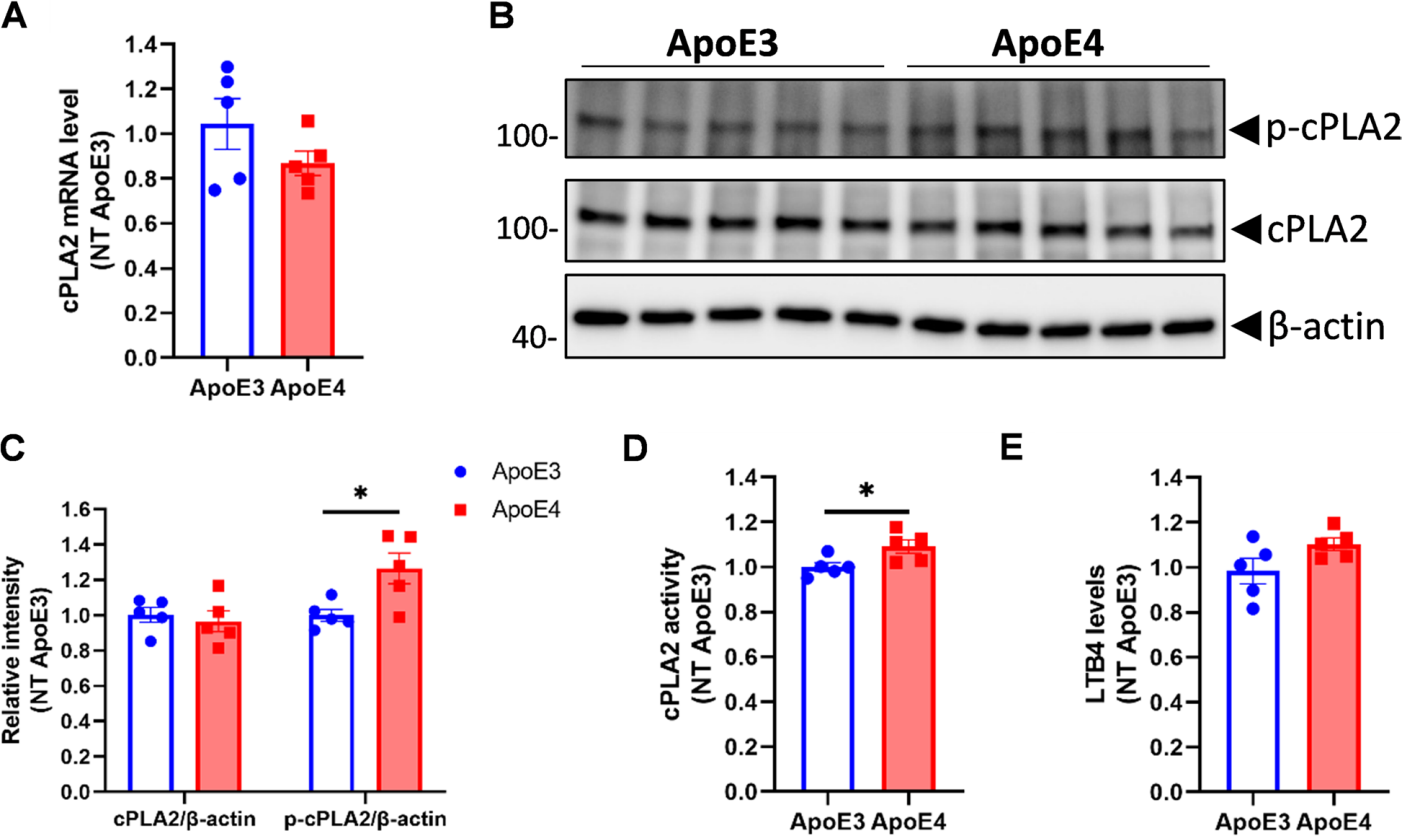
cPLA2 activity is greater in brains of ApoE4-TR mice compared with ApoE3-TR mice. The cortex of 8-month old ApoE3 and ApoE4-TR mice was collected to extract RNA and proteins. **A** cPLA2 mRNA level in the cortex was detected by qPCR. **B** Phosphorylated-cPLA2 and total cPLA2 protein levels in the cortex were detected by western blot. **D**ensitometric quantification from B. **D** cPLA2 activity in mouse cortex homogenates were measured by cPLA2 activity assay kit. **E** LTB4 levels in mouse cortical homogenates were measured by an LTB4 assay kit. (*n* = 5 for each genotype for A-E, 3 males and 2 females). LTB4: leukotriene B4. Data are represented as mean ± SEM and analyzed by Student’s t-test (two-tailed). *p < 0.05

To investigate the effect of the ApoE isoforms on cPLA2 in vivo, mRNA, total protein, and phosphorylated protein levels of cPLA2 were measured in the cerebral cortex from 8-month-old ApoE3-TR and ApoE4-TR mice. There was no difference in cortical cPLA2 mRNA levels between ApoE3-TR and ApoE4-TR mice (Fig. 2A). Since phosphorylated cPLA2 levels were too low to detect in total brain homogenates, cPLA2 was enriched by immunoprecipitation with a cPLA2 antibody using 500 μg of cortical homogenate, and total and phosphorylated cPLA2 levels were measured by Western blot. Total cPLA2 levels did not differ between ApoE3-TR and ApoE4-TR mouse cortex (Fig. 2B, C). However, phosphorylated cPLA2 was significantly increased in the ApoE4-TR mouse cortex compared to the ApoE3-TR mouse cortex (Fig. 2B, C). Consistent with these observations, cortical cPLA2 activity (based on the hydrolysis of the arachidonoyl thioester bond to release a detectable free thiol by endogenous brain PLA2) and leukotriene B4 (LTB4) levels (downstream product of AA release after cPLA2 activation) were higher in ApoE4-TR than ApoE3-TR mice (Fig. 2D, E).

### p38 MAPK but not ERK1/2 is increased in ApoE4 mouse primary astrocytes

**Fig. 3:**
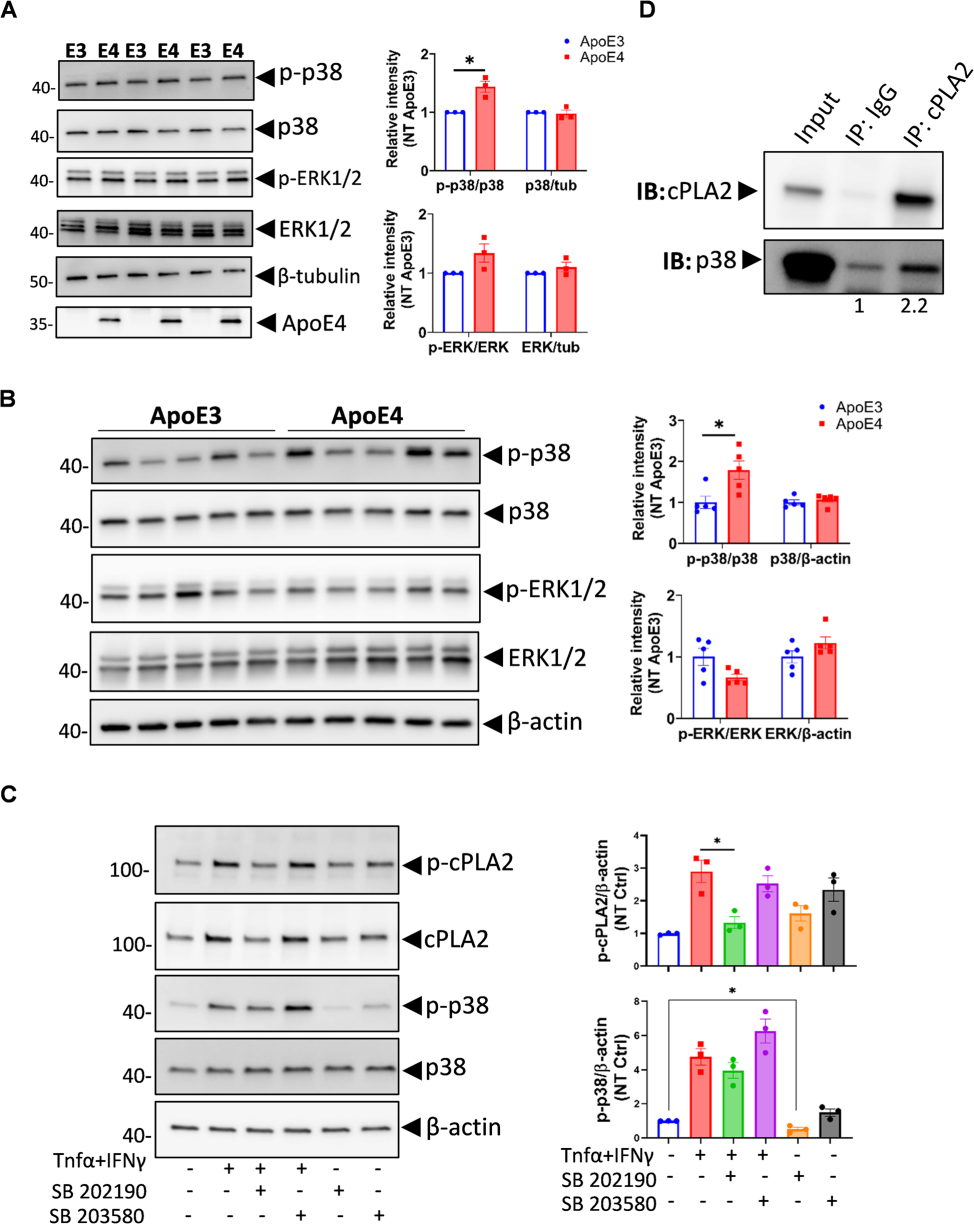
Increased phosphorylated-cPLA2 in *APOE4* is mediated by p38 MAPK. **A** Phosphorylated and total p38 and ERK levels in primary astrocyte from ApoE3 and ApoE4-TR mice were detected by WB (*n* = 3 for each genotype). **B** Phosphorylated and total p38 and ERK levels in cortical homogenates from ApoE3 and ApoE4-TR mice were detected by WB. (*n* = 5 for each genotype). **C** ApoE4 primary astrocytes from mouse were pre-treated with p38 inhibitors SB 202190 (10 μM) or SB 203580 (10 μM) for 20 minutes and then treated with medium or TNFα plus IFNγ together for 30 minutes. The total and phosphorylated cPLA2 and p38 were detected in the cell lysate by WB. Quantification was obtained from 3 independent repeats. **D** cPLA2 bound with p38. Immunoprecipitation was performed in the cell lysate of immortalized ApoE4 astrocytes using anti-cPLA2 antibody or species-matched IgG. cPLA2 and p38 were co-detected after immunoprecipitation by WB. WB: Western Blot. Data are represented as mean ± SEM and analyzed by Student’s t-test (two-tailed). *p < 0.05

Phosphorylation of cPLA2 is regulated by MAPK pathways, including p38 MAPK and ERK1/2 MAPK [10, 24, 25]. We tested the phosphorylation of p38 and ERK1/2 in primary astrocytes and mouse cortex from ApoE3 or ApoE4-TR mice by immunoblot using antibodies against total and phosphorylated proteins. Total p38 and ERK1/2 proteins did not differ between ApoE3 and ApoE4 primary astrocytes (Fig. 3A). Interestingly, only phosphorylated p38, but not phosphorylated ERK1/2, was significantly greater in ApoE4 primary astrocytes than ApoE3 primary astrocytes (Fig. 3A). In agreement, greater p38 phosphorylation but not ERK1/2 was evident in the cerebral cortex of 8-months old ApoE4-TR mice compared to ApoE3-TR mice (Fig. 3B). To test whether cPLA2 activation is dependent on p38 MAPK signaling, we treated ApoE4 primary astrocytes with two different p38 MAPK pathway inhibitors (SB202190 and SB203580) prior to the induction of cPLA2 activation with TNFα and IFNγ. The results showed that SB202190 significantly reduced phosphorylated (activated) cPLA2 levels (Fig. 3C). Interestingly, SB203580 had no inhibitory effects on cPLA2 activation (Fig. 3C), as SB203580 inhibited MAPKAPK-2 activity but not phosphorylation of p38 MAPK itself [26]. cPLA2 was found to be complexed with p38 as indicated by p38 co-immunoprecipitating with cPLA2 by anti-cPLA2 antibodies in immortalized ApoE4 astrocytes (Fig. 3D). These observations confirmed that p38 MAPK but not the ERK1/2 MAPK pathway regulate cPLA2 phosphorylation in ApoE4.

### Phosphorylated cPLA2 is increased in *APOE4* human brains

**Fig. 4:**
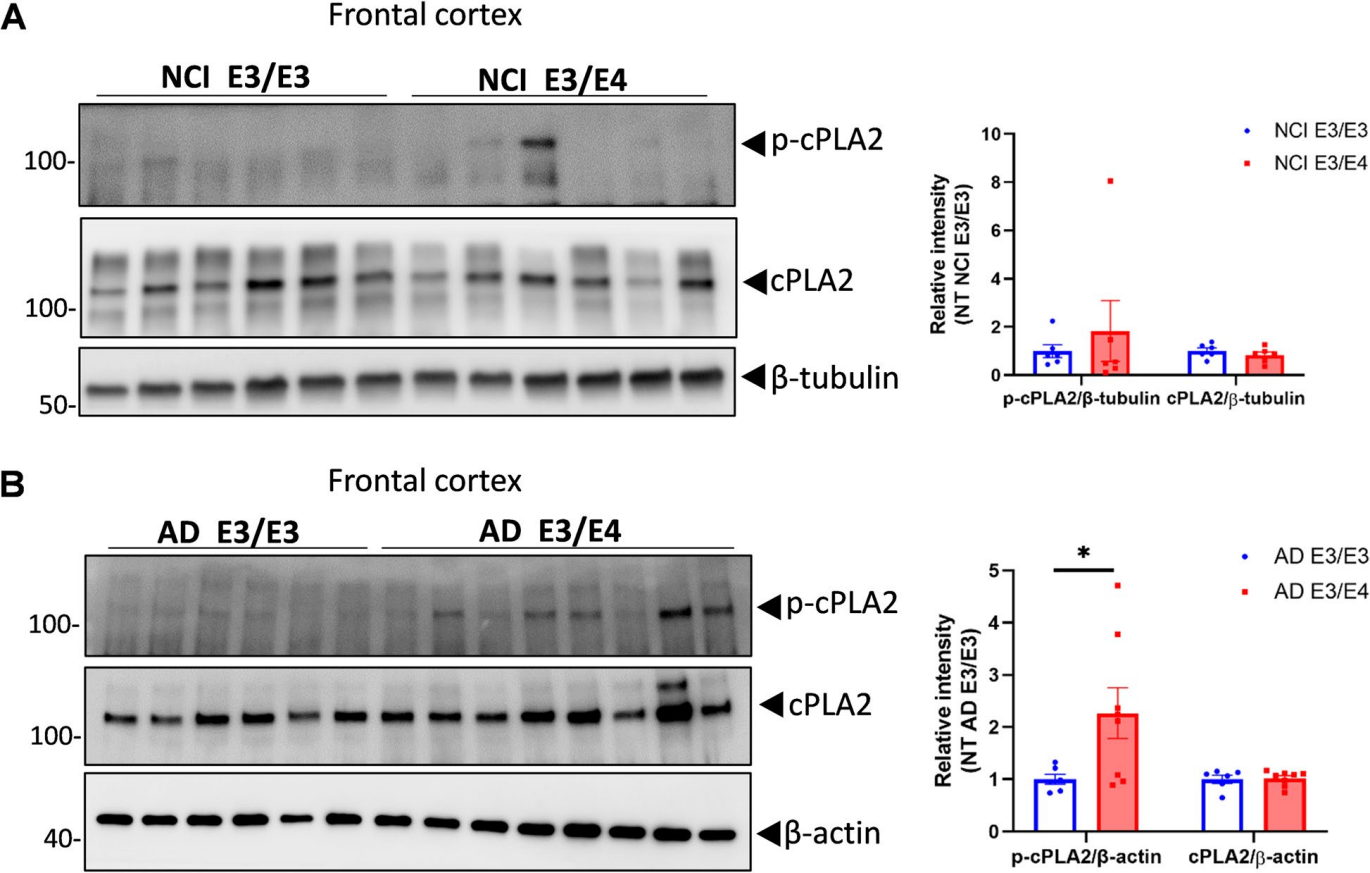
Phosphorylated-cPLA2 levels are greater in the frontal cortex of persons with AD dementia with *APOE3/E4* compared with *APOE3/E3*. The frozen inferior frontal lobe tissue from persons with NCI or AD dementia with different *APOE* genotypes were homogenized with RIPA buffer. cPLA2 was enriched by immunoprecipitation with anti-cPLA2 antibody and then the eluted complex was used to measure the phosphorylated cPLA2 (p-cPLA2) and total cPLA2 protein levels by Western blot. β-tubulin or β-actin were used as loading controls. **A** p-cPLA2 and cPLA2 protein levels in the cortex from persons with NCI (*n* = 6 for E3/E3, *n* = 6 for E3/4). **B** p-cPLA2 and cPLA2 protein levels in the cortex from persons with AD dementia (*n* = 6 for E3/E3, *n* = 8 for E3/E4). NCI, no cognitive impairment; AD, Alzheimer’s disease. Data are represented as mean ± SEM and analyzed by Student’s t-test (two-tailed). *p < 0.05

To determine whether these findings can be demonstrated in human brains, we compared phosphorylated and total cPLA2 in the inferior frontal cortex of persons with a similar clinical diagnosis but with different *APOE* genotypes. Characteristics of brain samples tested are summarized in Table 1. After enrichment of cPLA2 from the cortex, phosphorylated and total cPLA2 levels were measured by Western blot. In the NCI group, total cPLA2 did not significantly differ between the *APOE3/E3* and *APOE3/E4* carriers, while the phosphorylated cPLA2 level showed a trend increase in *APOE3/E4* carriers as compared to *APOE3/E3* carriers (Fig. 4A). In patients with AD, phosphorylated cPLA2 levels were significantly greater in *APOE3/*E4 carriers compared with *APOE3/E3* carriers, while the total cPLA2 levels did not differ between the two groups (Fig. 4B). Braak stage did not significantly differ between *APOE3/E3* and *APOE3/E4* carriers with AD dementia (*p* = 0.08). In a multivariate linear model, we did not find an association of cPLA2 phosphorylation with Braak stage (Fig. S6C). However, a larger sample size is needed to adequately address this question. A nonsignificant difference in soluble Aβ42 monomers was observed in the brains of *APOE3/*E4 carriers compared with *APOE3/E3* carriers with AD (Fig. S4).

**Table 1:** Characteristics of human samples

Regions sampled and source	Inferior frontal lobe (ROSMAP, RUSH ADRC)			
Clinical diagnosis	AD dementia	AD dementia	NCI	NCI
Genotype	E3/E3	E3/E4	E3/E3	E3/E4
Sample size, n	12	10	12	10
Age at death (years ± SD) ^a^	92 ± 6	95 ± 5	83 ± 5	85 ± 4
Sex (n, female/male) ^a^	5/7	6/4	6/6	5/5
Braak stage				
I	1	0	0	0
II	2	0	2	3
III	3	0	5	2
IV	0	0	4	5
V	6	10	1	0

^a^Age and sex did not differ between groups compared using ANOVA. *NCI* No cognitive impairment. *AD* Alzheimer’s disease

### p38 MAPK is increased in *APOE4* human brain samples

**Fig. 5:**
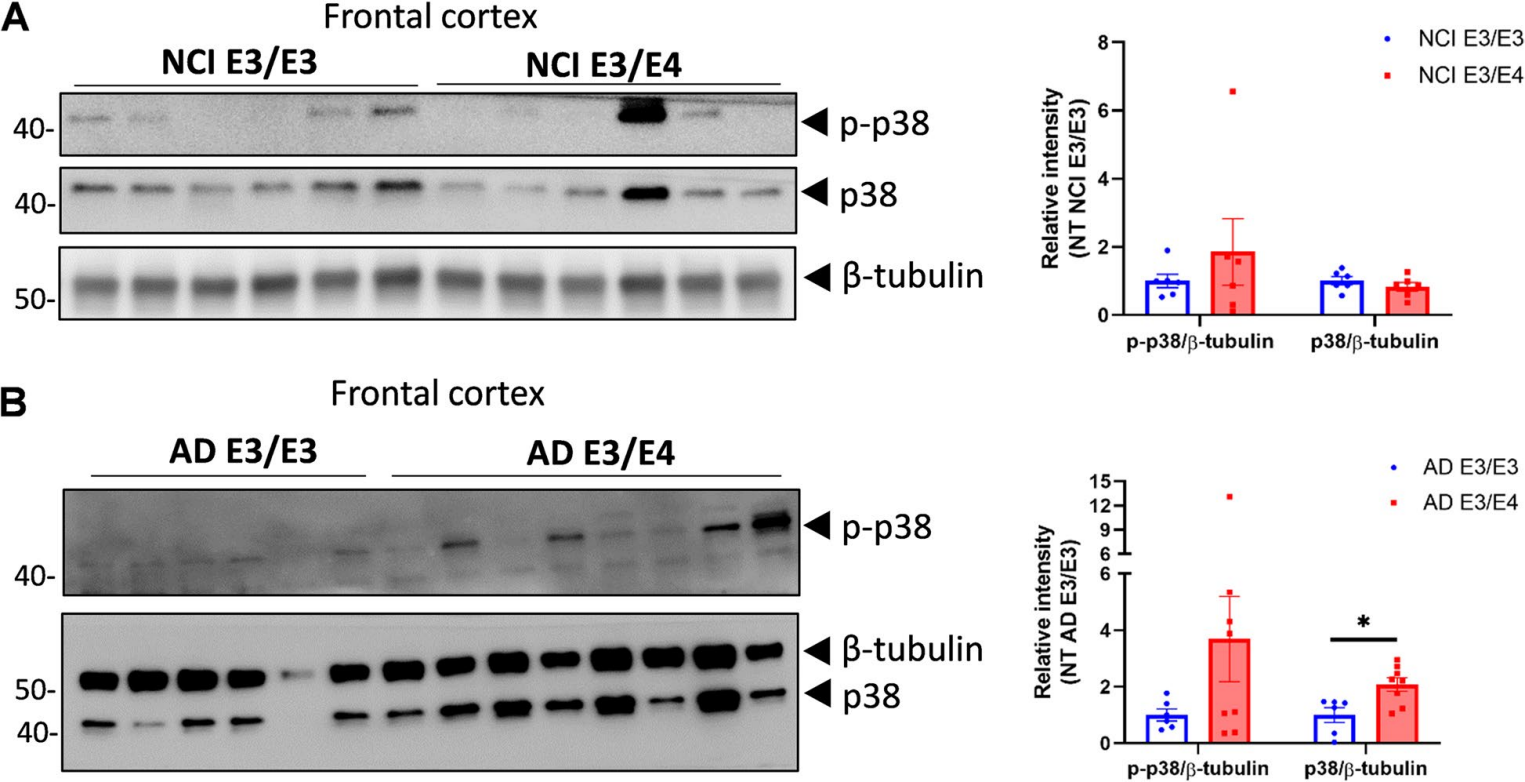
p38-MAPK levels are greater in the frontal cortex of persons with AD dementia with *APOE3/E4* compared with *APOE3/E3*. The frozen inferior frontal lobe tissue from persons with NCI or AD dementia with different *APOE* genotypes was homogenized to test phosphorylated p38 (p-p38) and total p38-MAPK protein levels using WB. **A** p-p38 and p38 protein levels in the cortex from persons with NCI (*n* = 6 for E3/E3; *n* = 6 for E3/4). **B** p-p38 and p38 protein levels in the cortex from persons with AD dementia (*n* = 6 for E3/E3; *n* = 8 for E3/E4). NCI, no cognitive impairment; AD, Alzheimer’s disease. WB: Western Blot. Data are represented as mean ± SEM and analyzed by Student’s t-test (two-tailed). *p < 0.05

Previous results from mouse astrocyte and cortex showed increased p38 activation in ApoE4-TR compared to ApoE3-TR mice. Phosphorylated and total p38 levels did not differ between NCI *APOE3/E3* and NCI *APOE3/E4* groups (Fig. 5A), while total p38 level was significantly greater in the AD *APOE3/E4* group compared to the AD *APOE3/E3* group (Fig. 5B). In a second brain cohort from the USC ADRC neuropathology core (Supplementary Table 1), nonsignificant differences were observed in phosphorylated cPLA2/total cPLA2 in the hippocampus of the *APOE4/E4* AD group compared to the *APOE3/E3* NCI group (Fig. S5A), despite a significantly greater ratio of phosphorylated p38/ total p38 (Fig. S5B). These results support that greater activation of p38 MAPK pathway with ApoE4 that is most prominent in persons with AD.

### LTB4 levels are increased in *APOE4* human brain samples

**Fig. 6:**
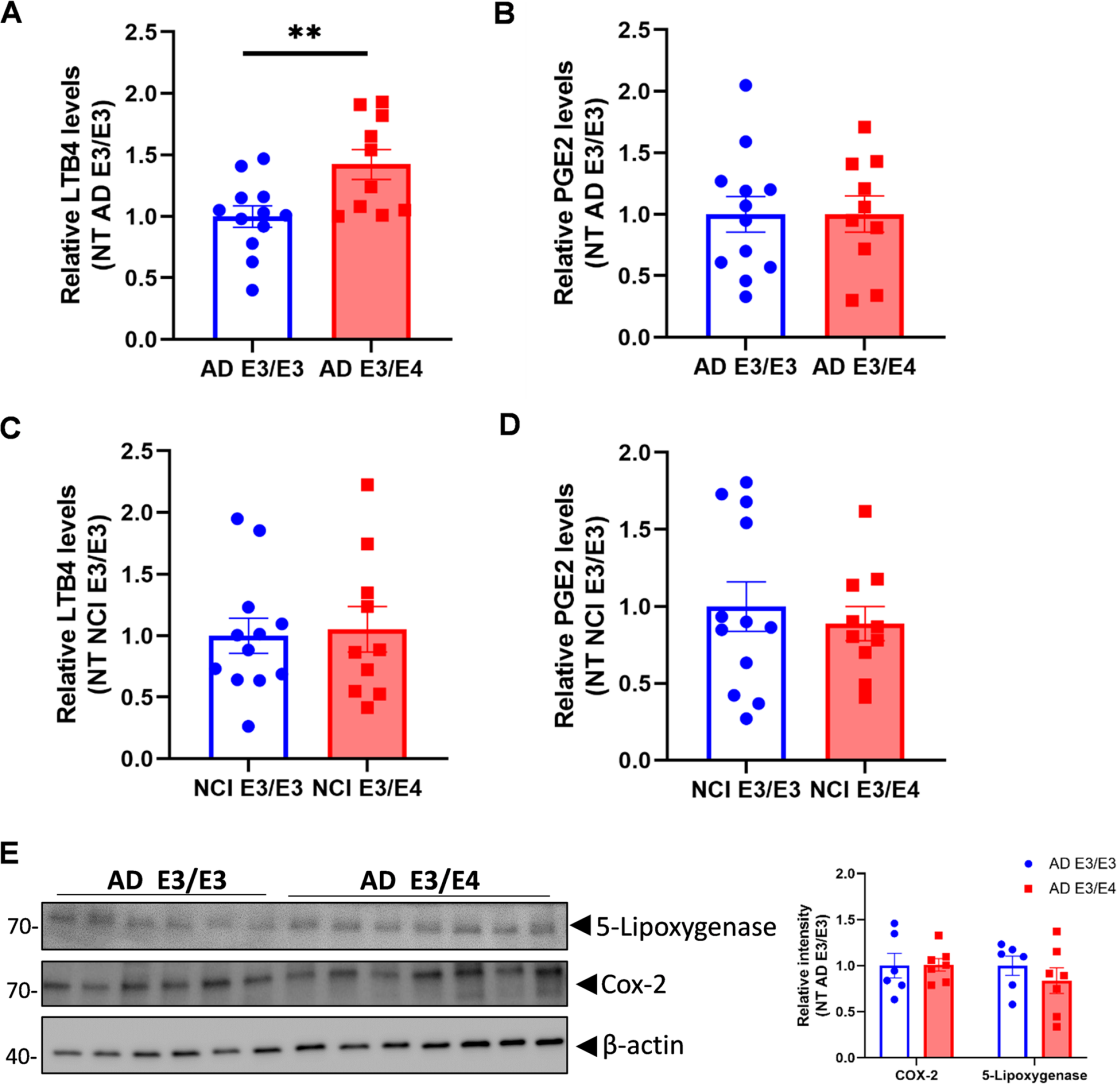
LTB4 levels are greater in the frontal cortex of persons with AD dementia in* APOE3/E4* carriers compared to *APOE3/E3* carriers. The frozen inferior frontal lobe tissue from persons with NCI or AD dementia with different *APOE* genotypes was homogenized and the supernatant was collected to measure the LTB4 (**A**, **C**) and PGE2 levels (**B, D**). (*n* = 12 for NCI E3/E3; *n* = 10 for NCI E3/E4; *n* = 12 for AD E3/E3; *n* = 10 for AD E3/E4). **E** 5-Lipoxygenase and Cox-2 protein levels in the RIPA homogenates of inferior frontal cortex from persons with AD dementia were detected by western blot. (*n* = 6 for E3/E3; *n* = 7 for E3/E4). NCI, no cognitive impairment; AD, Alzheimer’s disease; LTB4: leukotriene B4, PGE2: Prostaglandin E2; Cox-2: cyclooxygenase. Data are represented as mean ± SEM and analyzed by Student’s t-test (two-tailed). **p < 0.01

AA is released by cPLA2 hydrolysis of membrane phospholipids, and then can be rapidly oxidized by COX or LOX enzymes to prostaglandins or leukotrienes (LTB4 and PGE2), potent mediators of inflammation and signal transduction [2]. To test the effect of the greater cPLA2 phosphorylation in *APOE4* AD brains, PGE2 and LTB4 levels were assayed in brain homogenates from the inferior frontal cortex. LTB4 levels were significantly greater in the AD *APOE3/4* group compared with the AD *APOE3/3* group (Fig. 6A), while PGE2 levels did not differ between the two groups (Fig. 6B). The greater LTB4 levels in APOE3/E4 group were also not affected by sex, age, or Braak stage. No significant differences were found in either LTB4 or PGE2 levels between the NCI *APOE3/3* and NCI *APOE3/4* groups (Fig. 6C and D). The expression of 5-LOX and COX-2 did not differ between the AD *APOE3/3* and AD *APOE3/4* groups (Fig. 6E). These results indicate that ApoE4’s activation of cPLA2 in AD selectively increases LTB4 levels in the AD brain.

### The NF-kB inflammasome is not induced in the *APOE4* brain

**Fig. 7:**
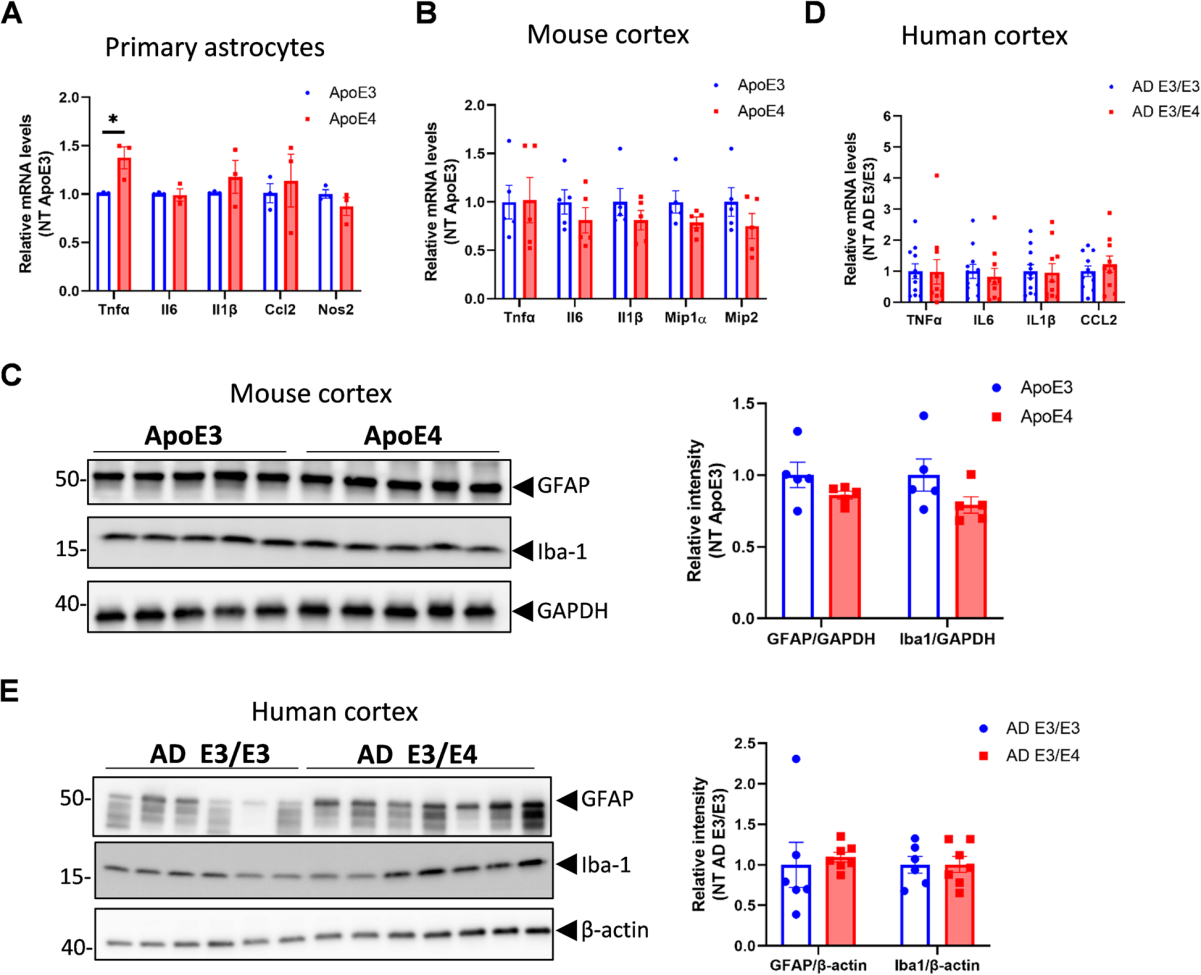
Selected inflammatory mediators do not differ by *APOE* genotype in primary astrocytes, mouse, and human cortex. **A** mRNA levels of some of the proinflammatory markers in primary astrocyte from ApoE3-TR or ApoE4-TR mice (*n* = 3 for each genotype). **B** mRNA levels of some of the proinflammatory cytokines in cortex of ApoE3-TR or ApoE4-TR mice. **C** GFAP, and Iba-1 expression in cortex of ApoE3-TR or ApoE4-TR mice. (*n* = 5, 3 males and 2 females for B and C). **D** mRNA levels of some of the proinflammatory markers in inferior frontal cortex from persons with AD dementia (*n* = 11 for E3/E3; *n* = 10 for E3/E4). **E** GFAP and Iba-1 expression in inferior frontal cortex from persons with AD dementia (*n* = 6 for E3/E3, *n* = 7 for E3/E4). AD, Alzheimer’s disease. Data are represented as mean ± SEM and analyzed by Student’s t-test (two-tailed). *p < 0.05

It is not clear whether *APOE4* can induce neuroinflammation via activation of the NF-kB inflammasome in vivo, and whether cPLA2 is involved in this pathway. Although we found greater TNFα mRNA levels in ApoE4 than in ApoE3 astrocytes, IL1β, IL6 and Ccl2 did not differ between ApoE3 and ApoE4 astrocytes (Fig. 7A). In addition, the mRNA levels of these cytokines and chemokines were comparable in different ApoE genotypes from mice brains (Fig. 7B) or the human brain samples (Fig. 7D). Similarly, the abundance of glial fibrillary acid protein (GFAP) in astrocytes and ionized calcium binding adaptor molecule 1 (Iba1) in microglia also did not differ by genotype (Fig. 7D-E). No associations were found between the p-cPLA2 and GFAP or Iba1 levels in human cortex samples (Fig. S6). These results indicate that neuroinflammation with *APOE4* does not favor the NF-kB inflammatory response pathway.

### cPLA2 is involved in the ApoE4 mediated up-regulation of LTB4 and ROS

**Fig. 8:**
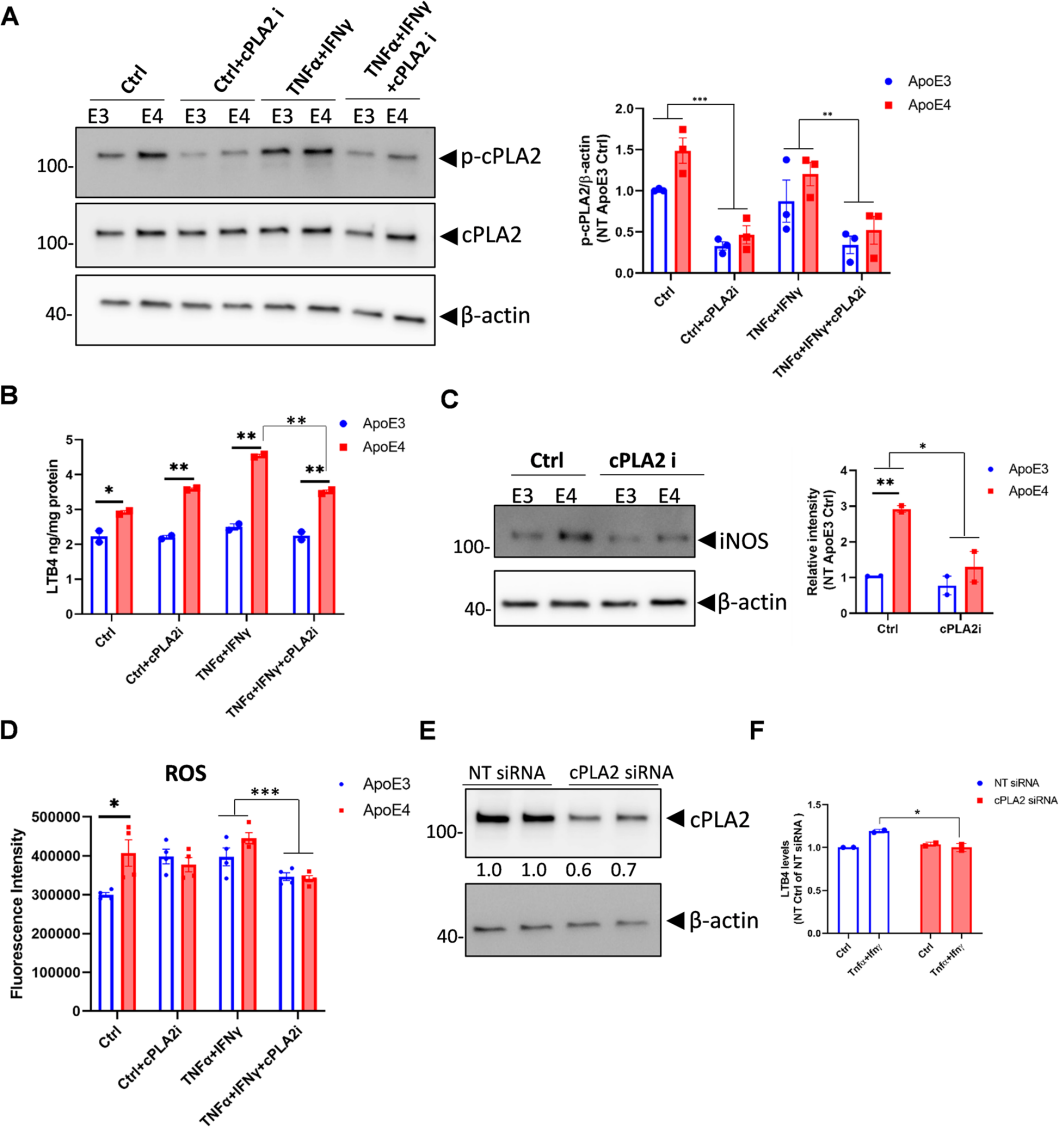
Inhibition of cPLA2 reduces ApoE4 mediated up-regulation of LTB4, ROS, and iNOS levels. **A**, **B** and **C** ApoE3 and ApoE4 primary astrocytes from mice pups were pre-treated with medium (Ctrl) or the cPLA2 inhibitor (pyrrophenone) for 30 min followed by treatment with TNFα plus IFNγ together for 18 hours. **A** A representative blot showing total and phosphorylated-cPLA2 levels. The quantification was from 3 independent repeats. **B** LTB4 levels in the culture medium were measured by the assay kit. **C** A representative blot showing iNOS expression in cell lysate. The quantification was from 2 independent repeats. **D** ApoE3 and ApoE4 primary astrocyte were pre-treated with pyrrophenone for 30 min and then treated with medium or TNFα plus IFNγ together for 24 h. ROS levels were detected by the DCFDA probe. **E**, **F** ApoE4 primary astrocytes were transfected with cPLA2 siRNA or non-target (NT) siRNA for 48 hours and then treated with medium or TNFα plus IFNγ together for 24 hours. **E** cPLA2 protein levels in cell lysate were detected by WB. **F** LTB4 levels in the culture medium were measured by the assay kit. WB, Western Blot. DCFDA: 2′,7′–dichlorofluorescin diacetate. LTB4: leukotriene B4. Data are represented as mean ± SEM and analyzed by Student’s t-test (two-tailed). Two-way ANOVA was used in A, C, and D for group comparisons. *p < 0.05, **p < 0.01, ***p < 0.001

To explore whether cPLA2 inhibition mitigates the downstream effects of LTB4 production on ROS and iNOS, ApoE3 and ApoE4 primary astrocytes were treated with the cPLA2 inhibitor - pyrrophenone (Fig. 8A). Treatment with pyrrophenone reduced LTB4 levels in ApoE3 and ApoE4 astrocytes, but to a greater extent in ApoE4 astrocytes (Fig. 8B). Furthermore, cPLA2 inhibition significantly decreased iNOS and ROS levels in ApoE3 and ApoE4 primary astrocytes (Fig. 8C, D). These results indicated that greater cPLA2 activity promoted greater levels of iNOS and ROS in the ApoE4 group and can be reduced with cPLA2 inhibition. To confirm the specific effect of cPLA2 in LTB4 production, we knocked down cPLA2 using small interfering RNA (siRNA) in ApoE4 primary astrocytes (Fig. 8E). In agreement, LTB4 levels were significantly decreased in the cPLA2 siRNA treatment group compared to the non-target siRNA treatment group (Fig. 8F).

### rE4 and Aβ_42_ induce greater cPLA2 translocation into the cytosol than rE3 in human synaptosomes

**Fig. 9:**
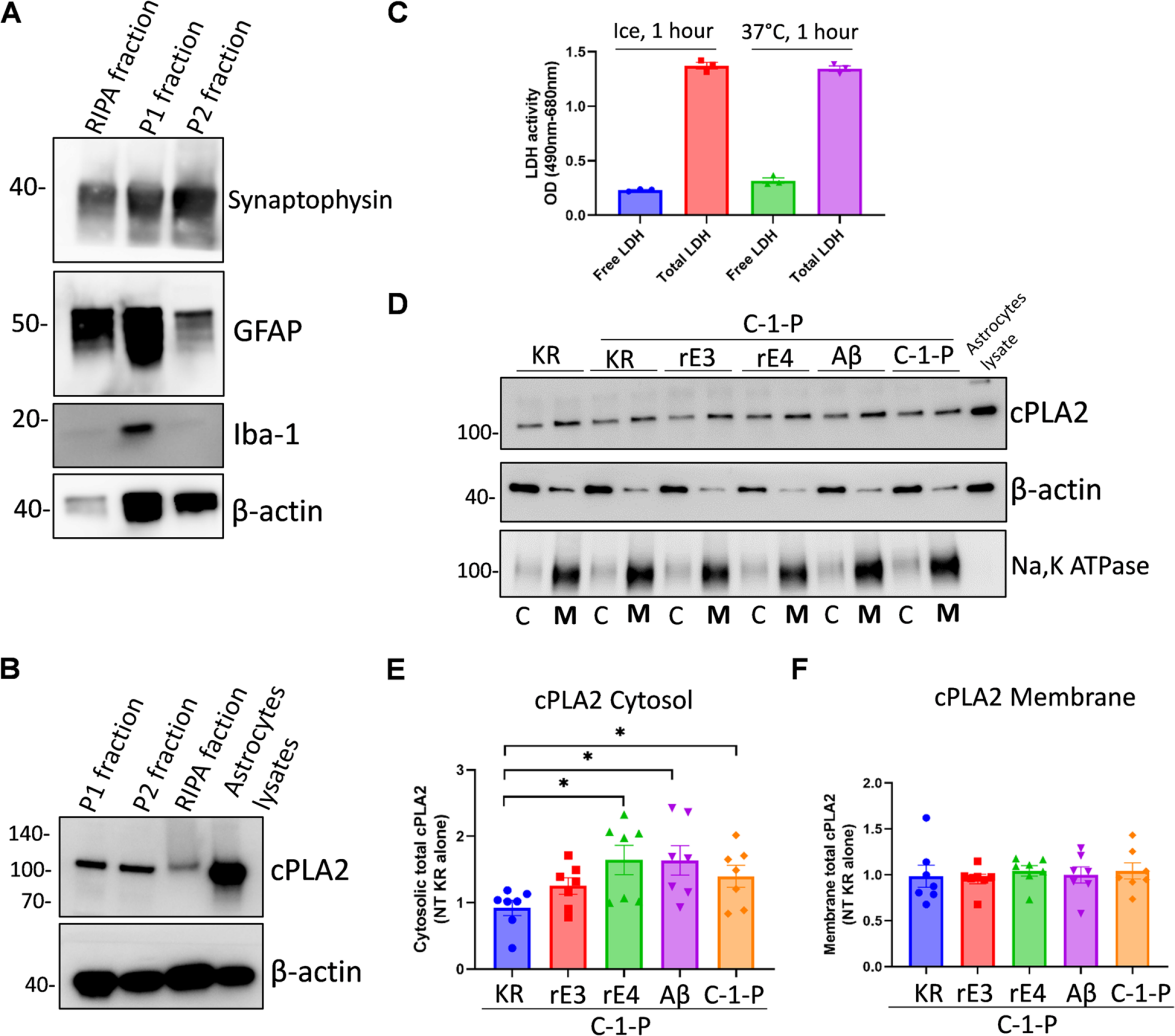
rE4 and Aβ42 induce greater cPLA2 translocation to synaptic vesicles (cytosolic fraction) in human synaptosomes than rE3. **A** Frozen postmortem human cortex samples were prepared to collect the supernatant (P1 fraction) and pellets (P2 fraction, synaptosomes) and the different cell type markers were measured by WB. **B** cPLA2 expression was detected in different fractions. **C** The LDH assay was performed to test the membrane integrity of the isolated synaptosomes after incubation for 1 hour at different temperatures. **D** One group of synaptosomes was treated with KR alone (control), and the remaining groups were treated with rE3, rE4, Aβ42 and C-1-P for 30 minutes, followed with C-1-P treatment for 15 minutes. After treatment, the cytosolic and membrane fractions of synaptosomes were isolated by centrifugation. cPLA2 was enriched by immunoprecipitation with anti-cPLA2 antibody and then phosphorylated and total cPLA2 were measured by WB. A representative blot for total cPLA2 is shown. Phosphorylated cPLA2 levels were low and not shown. **E** Quantification of cPLA2 protein expression in the cytosol fraction. cPLA2 was normalized to β-actin for each group and then adjusted to KR alone for all groups. **F** Quantification of cPLA2 protein expression in membrane fraction. cPLA2 protein expression was normalized to Na,K ATPase for each group and then adjusted to KR alone for all groups. For **E** and **F**, data was obtained from 7 independent repeats. WB. Western blot; C, cytosol; M, Membrane; rE3, recombinant ApoE3; rE4, recombinant ApoE4; C-1-P, Ceramide-1-phosphate; KR, Krebs Ringer buffer. Data presented as mean ± SEM and analyzed by Student’s t-test (two-tailed). *p < 0.05

Since cPLA2 was shown to be expressed in neurons and activated by Aβ monomers [27], we examined the effect of exogenous Aβ_42_ and rE on its activation in synaptosomes from human postmortem frontal cortices obtained from control participants without AD pathology. First, we successfully isolated synaptosomes from frozen human cortex (P2 fraction, which is synaptophysin enriched fraction) (Fig. 9A) and confirmed cPLA2 expression (Fig. 9B). Then, we verified that the membrane integrity of synaptosomes would be conserved after 1 hour incubation at 37 °C (Fig. 9C) by the LDH assay [28]. Isolated synaptosomes were pretreated with rE3, rE4 and Aβ_42_ followed by the treatment with C-1-P to induce cPLA2 translocation. The translocation cPLA2 into cytosolic synaptic vesicles activates Ca^2+^ mediated neurotransmission [29], and C-1-P is known to enhance this translocation to Golgi derived membranes [30]. Significantly greater levels of cytosolic cPLA2 were observed in rE4 and Aβ_42_ pre-treated groups compared to KR control. In contrast, rE3 pre-treatment had no significant effects on cytosolic cPLA2 in synaptosomes (Fig. 9D, E). Membrane cPLA2 levels did not differ by treatment groups (Fig. 9D, F). The levels of phosphorylated cPLA2 in synaptosomes with or without treatment were low and were not quantified. These results indicated rE4 and Aβ_42_ could induce more cPLA2 translocation than rE3 in the vesicle enriched cytosolic fraction of synaptosomes, suggesting that greater cPLA2 activation in the human cortex of AD E3/E4 compared to AD E3/E3 might arise from the concurrent effects of ApoE4 and greater Aβ_42_ accumulation.

## Discussion

Despite multiple past observations associating *APOE4* with greater neuroinflammatory and oxidative stress response than *APOE2* or *APOE3* (Table 2), the underlying mechanisms are not clearly understood. Here, we identify a plausible mechanism where *APOE4* induces greater activation of the cPLA2 system in both astrocytes and synaptosomes, with greater release of AA, LTB4, iNOS, and generation of ROS in astrocytes. The increase of LTB4 in *APOE4* was corroborated in human brain samples matched by disease state. Inhibition of cPLA2 activity lowered the greater neuroinflammation associated with *APOE4*, reinforcing the candidacy of cPLA2 as a therapeutic target for mitigating the increase in AD risk conferred by carrying *APOE4*.

**Table 2:** Summary of the association of *APOE4* with greater neuroinflammation

Author	Key findings
**Cultures (microglia, astrocytes, or mixed cultures) and inflammatory response by genotype**	
Vitek et al. [31]	Microglia derived from ApoE4-TR mice demonstrate increased NO production, increased NOS2 mRNA levels, and greater TNFα, IL-6, IL12 levels compared to microglia from ApoE3-TR mice.
Colton et al. [32]	Significantly more NO was produced in primary microglia and macrophages from ApoE4-TR mice compared to ApoE3-TR mice.
Guo et al. [33]	The addition of exogenous ApoE4 induced greater IL1β than ApoE3 in rat mixed glial cells.
Chen et al. [34]	ApoE4, but not ApoE3, stimulated secretion of PGE2 and IL-1β in rat primary microglia.
Shi et al. [35]	Higher TNFα, IL1β, and IL1α levels were observed in primary microglia from ApoE4-TR mice stimulated with LPS than ApoE2 and ApoE3.
Tai et al. [36]	Greater astrogliosis and microgliosis, higher levels of IL1β in E4FAD mice compared with E3FAD and E2FAD mice.
Zhu et al. [37]	Higher levels of microglia/macrophage, astrocytes, and invading T-cells after LPS injection in ApoE4-TR mice than ApoE3-TR mice. ApoE4-TR mice also displayed greater and more prolonged increases of cytokines (IL1β, IL6, TNFα) than ApoE2 and ApoE3-TR mice.
Ophir et al. [38]	The expression of inflammation-related genes (NF-κB response elements) following intracerebroventricular injection of LPS was significantly higher and more prolonged in ApoE4 than in ApoE3-TR mice.
**Both human and mouse models**	
Gale et al. [39]	ApoE4-TR mice displayed enhanced plasma cytokines after systemic LPS compared with ApoE3 counterparts. After intravenous LPS, *APOE3/4* patients had higher plasma TNF-α levels than *APOE3/3* patients.
**Human brain studies of inflammation and oxidative stress studies by** ***APOE*** **genotype**	
Montine at al [40]	Pyramidal neuron cytoplasm was immunoreactive for 4-hydroxy-2-nonenal (HNE) in 4 of 4 *APOE4* homozygotes, 2 of 3 *APOE3/4* heterozygotes, and none of 3 *APOE3* homozygotes
Ramassamy et al. [20]	In hippocampal homogenates from AD brains, *APOE4* carriers had greater levels of thiobarbituric acid-reactive substances (TBARS), lower catalase activity, and glutathione peroxidase and glutathione than tissues from patients homozygous for the *APOE3* allele (*n* = 10 per group).
Egensperger et al. [41]	The number of activated microglia and the tissue area occupied by these cells increased significantly with the *APOE4* gene dose (*n* = 20).
Minett et al. [42]	*APOE4* allele was significantly related to greater expression of CD68, HLA-DR, and CD64 in microglia (*n* = 299).
Friedberg et al. [43]	Cellular density of microglial marker-Iba1 was positively associated with tau pathology in *APOE4* carrier participants only (*n* = 154).
**Systemic inflammation and dementia risk by genotype**	
Tao et al. [19]	Participants with *APOE4* and elevated plasma C reactive protein (CRP) levels had a shortened latency for the onset of AD (*n* = 2562).

There is evidence from clinical studies implicating greater cPLA2 activation around AD brain plaques [12]. cPLA2 activity is also increased in the CSF of patients with AD [44]. cPLA2 activation can be indirectly assessed by the release of AA from membrane phospholipids [2]. ^11^C AA brain uptake by PET and unesterified AA/DHA measurement in CSF are surrogate brain cPLA2 activity markers. Indeed, greater incorporation coefficients of ^11^C AA by PET scans were observed in the grey-matter regions of the brain of AD patients compared to control subjects [45]. Moreover, a greater AA/DHA ratio in both CSF and plasma was present in *APOE4* carriers with mild AD compared to *APOE3* carriers after DHA supplementation [21]. A greater AA/DHA ratio in plasma phospholipids in cognitively healthy *APOE4* carriers was associated with greater conversion to MCI/AD [46]. The greater plasma AA/DHA in *APOE4* suggests a systemic (for example, in the liver) activation of cPLA2 that is not just confined to the brain.

Our studies in human brains revealed that carrying an *APOE4* allele is not sufficient to activate cPLA2. This is unsurprising as not all *APOE4* carriers develop AD pathology. cPLA2 activation was significantly greater in *APOE4* carriers compared to *APOE3* carriers with AD, but not in those with NCI. We also found rE4 and Aβ42 induced more cPLA2 translocation in postmortem frontal lobe synaptosomes (Fig. 9). One biological explanation is that the effects of soluble Aβ oligomers in AD is additively intensified by ApoE4 to promote a neuroinflammatory phenotype. We speculate that treatments which reduce activation of cPLA2 especially in *APOE4* carriers can protect these subjects from neuroinflammation and neurodegeneration, but this hypothesis is yet to be proven. In contrast to observations made in human brains, the activation of cPLA2 in APOE4 KI mouse models in both primary astrocytes and animal brains was measured independent of Aβ. The APOE4 KI mouse models used here are *APOE4* homozygous and are maintained under a controlled environment that can allow for observing a greater *APOE4* effect than with human studies.

Greater cPLA2 activation is mechanistically involved in AD pathology and may represent one pathophysiological link between Aβ oligomers and neuroinflammatory responses [47]. An increase of phosphorylated cPLA2 but not of total cPLA2 was observed in the brains of AD mouse models compared with WT mice [14]. In vitro studies suggested that Aβ oligomers can trigger cPLA2 activation and PGE2 production in neurons, eventually leading to neurodegeneration [27, 48]. Inhibition of cPLA2 prevented synaptic loss and memory deficits induced by Aβ oligomers in mice [49]. Similar to Aβ, there is evidence that human prion peptide can also induce neurotoxicity by activating cPLA2, which can be prevented by cPLA2 inhibition [50]. In support of greater cPLA2 activity, hippocampal levels of AA and AA-derived metabolites were much greater in hAPP mice than in non-transgenic control mice [14].

The pattern of enhanced neuroinflammation of the *APOE4* AD brains observed in this study does not support the induction of the NF-ĸB inflammasome by cytokines or chemokines such as TNFα, IL1β, IL6, and Ccl2, as past findings supporting these activation patterns were mostly a result of high doses LPS injections in cell culture and in vivo animal models (summarized in Table 2). Instead, we found a greater level of leukotrienes (LTB4) in the cerebral cortex of AD with E3/E4 carriers compared to E3/E3 carriers and ApoE4 astrocytes, which was associated with the greater phosphorylation of cPLA2. These observations provide a mechanism for the greater levels of oxidative stress in the *APOE4* brain [20, 40]. It is plausible that astrocytes and microglia contribute to the greater LTB4, ROS, and iNOS production with *APOE4*. An extensive recent proteomic and lipidomic investigation in animal brains of ApoE-TR mice corroborates the enhanced eicosanoid signaling with *APOE4* [51]. LTB4 signaling may have a prominent role in inducing oxidative stress. Chuang et al. reported that ROS and NO production during microglia activation is reduced by inhibition of lipoxygenase but not cyclooxygenase [8], suggesting induced LOX signaling as the primary driver of oxidative stress.

Activation of cPLA2 may differ by cell type and within cellular compartments. Recently, astrocytic activation of cPLA2 bound directly with MAVS enhanced NF-ĸB pathways to produce proinflammatory factors such as Ccl2 and Nos2 in an animal model of multiple sclerosis (MS) [7]. The fact that we did not observe greater CcL2 or Nos2 expression in *APOE4* astrocytes, mouse, or human brains in our current study suggests the selective activation cPLA2 by location within the astrocyte leading to a distinct neuroinflammatory phenotype. In addition to MS, the increase in AA release and its metabolism to prostaglandins and leukotrienes have been observed in cancers and other neurodegeneration diseases [52–54]. For example, *PIK3CA* mutant breast cancer tumor cells displayed dramatically elevated AA and eicosanoid levels, promoting tumor cell proliferation [53].

The activation of MAPK system by ApoE4 likely involves complex set of ApoE receptors or signaling pathways. In neurons, ApoE4 was shown to produce greater activating of the MAPK/ERK system (isoform dependent manner) to induce greater production of APP [55]; however, it was not clear if this activation involved ApoE signaling receptors (e.g., ApoER2 and VLDLR) or metabolic receptors (e.g., LRP1 and LDLR). Further studies are needed to sort out the receptor(s) involved in different cell types. That could help elucidate the physiological and pathological pathways relevant to ApoE and/or the receptors and their effect of P38-cPLA2 signaling.

Activation of cPLA2 activity is associated with its phosphorylation [10]. cPLA2 phosphorylation is regulated by ERKs and p38 MAPK pathways, which phosphorylates cPLA2 at Ser-505 and increases its enzymatic activity [9]. cPLA2 phosphorylation and AA release in response to PMA and ATP stimulation in mouse astrocytes are mediated by ERKs and p38 MAPK pathways [10]. In the platelets, cPLA2 phosphorylation was induced by p38 MAPK activation [24]. Here, we found that ApoE4 selectively activated p38 but not ERKs, and inhibition of p38 in ApoE4 astrocytes decreased cPLA2 activation. This activation of p38 is consistent with a previous report of greater p38 activation but not ERKs pathway in ApoE4-TR mice [56]. Interestingly, p38 inhibitors are in drug development pipelines for AD [57].

Our study has several strengths and some limitations. We confirmed our findings of greater cPLA2 activation in several independent models: primary cells, synaptosomes, in ApoE-TR animal models, and in human brains matched by disease stage and differing by genotype. We identified the signaling pathway involved in cPLA2 activation- (MAPK-p38) and validated this in both animal and human brains. Some of the limitations include not defining the cell-specific cPLA2 activation profile in vivo (such as astrocytes, microglia, neurons and oligodendrocytes). In the clinical cohort, we did not study cPLA2 expression in *APOE4* homozygote patients without cognitive impairment, as this condition is infrequent. We also acknowledge that the small sample sizes the human brain cohort that can preclude the full examination of the effect of sex and other AD risk factors on the association between *APOE4* and neuroinflammation. Larger sample sizes may identify associations between cPLA2 activation and Braak stages among persons with AD dementia with or without *APOE4*. Future studies will include larger sample sizes and more specific approaches (such as single-cell sequencing) to capture cPLA2’s activation fingerprint on different brain cell types.

## Conclusions

**Fig. 10:**
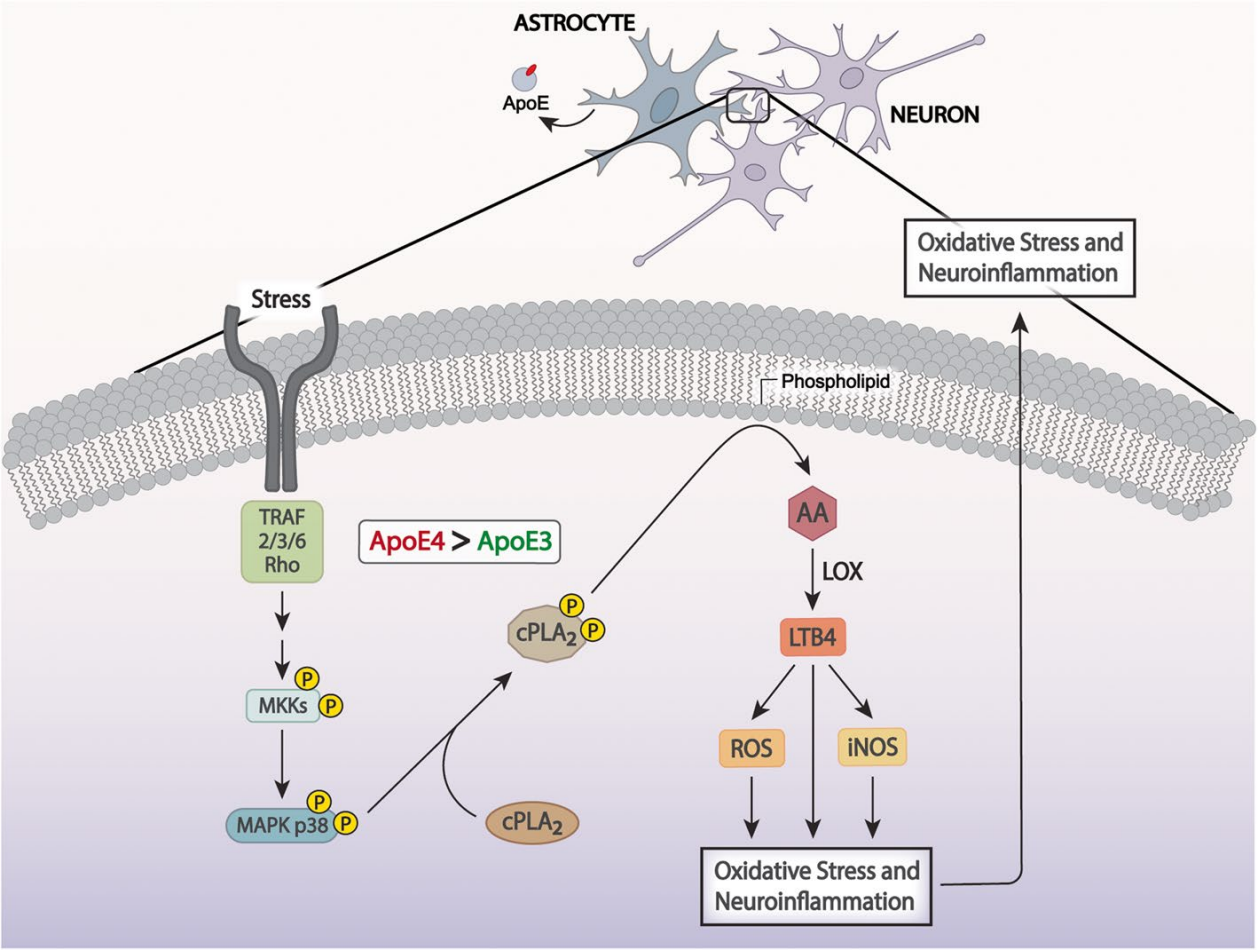
Illustration of ApoE4 in astrocytes and neurons inducing greater cPLA2 activation than ApoE3 through p38 MAPK pathway, leading to more LTB4, iNOS, and ROS production, increased oxidative stress and neuroinflammation

Overall, using multiple approaches, our study has identified that the activation of cPLA2 is implicated in neuroinflammation and oxidative stress associated with *APOE4* (Fig. 10). Our findings support the induction of the MAPK-p38 pathway as the driving factor for the activation of the cPLA2-LTB4 signaling cascade, and our cellular studies prioritize astrocytes as the target cell type. Inhibition of brain cPLA2 signaling may provide an attractive strategy to reduce the risk of AD dementia associated with carrying the *APOE4* allele.

## Materials and methods

### Clinical samples

The frozen hippocampi of persons with AD dementia with *APOE4/E4* carriers (*N* = 9) and no-cognitive impairment (NCI) with *APOE3/E3* carriers (*N* = 7) were collected from the University of Southern California (USC) Alzheimer Disease Research Center (ADRC) Neuropathology core, which was approved by USC’s Institutional Review Board (IRB) protocol (HS-16-00888). The frozen inferior frontal lobe (Brodmann area 10) of the individuals with NCI and the *APOE3/E3* carriers (*N* = 12) and *APOE3/E4* carriers (*N* = 10) and persons with AD patients and the *APOE3/E3* (*N* = 12) and *APOE3/E4* genotypes (*N* = 10) were obtained from the Rush Alzheimer’s Disease Center (RADC) at the Rush University Medical Center. Rush Memory and Aging Project was approved by an Institutional Review Board (IRB) of Rush University Medical Center.

### Animals

ApoE3-TR and ApoE4-TR mice were a generous gift from Dr. Patrick Sullivan. The endogenous mouse ApoE was replaced by either human APOE3 or APOE4, created by gene targeting, as described previously [58]. All experiments were performed on age-matched animals (8 months of age) and were approved by the USC Animal Care Committee. Every effort was made to reduce animal stress and to minimize animal usage. The mice were anesthetized with isoflurane and perfused with PBS. The brains were split in half for further analysis.

### Cell cultures

Primary astrocytes were obtained from C57JB6, ApoE3-TR, and ApoE4-TR mice pups and cultured, as described previously [59]. Briefly, cerebral cortices from each 1 to 3 day-old neonatal mouse were dissected in ice-cold Hanks’ Balanced Salt Solution (HBSS) (Corning, 21–021-CV) and digested with 0.25% trypsin for 20 min at 37 °C. Trypsinization was stopped by the addition of a 2-fold volume of DMEM (Corning, 10–013) with 10% fetal bovine serum (FBS) (Omega Scientific, FB-12) and 1% antibiotic-antimycotic (Anti-anti) (Thermo Fisher, 15,240,062). The cells were dispersed into a single-cell level by repeated pipetting and filtered through 100 μm cell strainers (VWR, 10199–658). After filtering, cells were centrifuged for 5 min at 1000 rpm and resuspended in a culture medium supplemented with 10% FBS and antibiotics. Then, cells were seeded in a 75 cm^2^ flask and cultured at 37 °C in 5% CO2. The medium was changed on the next day and then replaced every 3 days. These mixed glia cultures reached confluence after 7–10 days. The cells were then shaken at 250 rpm for 16 h at 37 °C to remove microglia and oligodendrocyte progenitor cells. The remaining cells were harvested by digestion with trypsin. At this stage, the culture contained 95% astrocytes and was used for further experiments.

Immortalized mouse astrocytes derived from human ApoE3 and ApoE4 knock-in mice [60] were gifts from Dr. David Holtzman and grown in DMEM/F12 (Corning, MT10090CV) containing 10% FBS, 1 mM sodium pyruvate (Thermo Fisher, 11,360,070), 1 mM geneticin (Thermo Fisher, 10,131–035) and 1% anti-anti.

### cPLA2 antibody validation

cPLA2 antibodies from Santa Cruz Biotech (sc-376,636, sc-376,618 and sc-454, 1:400) and Sigma (SAB4502200, 1:1000) were tested in astrocytes transfected with cPLA2 siRNA (15 nM) for 48 hours, then cells were lysed with RIPA buffer. After transfer and blocking, antibodies were added and incubated overnight at 4 °C. HRP-anti-mouse or HRP-anti-rabbit were used as secondary antibodies (Supplementary Figure 1 A). To test the phosphorylated cPLA2 antibody, astrocytes were treated with 100 μM ATP for 20 minutes. Cells were lysate with RIPA buffer with proteinase and phosphatase inhibitors. After centrifugation, the supernatants were collected and added with 4X sample buffer followed by boiling 5 minutes at 95 °C. Then 10 μL of lysates were loaded into 4–15% TGX gel (Bio-rad). After transferring and blocking, phospho-cPLA2 antibody (#53044, cell signaling technology) (1:1000) was added and incubated overnight at 4 °C. HRP-anti-rabbit was used as secondary antibody. The membrane was stripped with stripping buffer for 15 minutes at room temperature after imaging and blocked again for 1 hour with 5% milk. cPLA2 antibody (sc-376,618, Lot J1521, Santa Cruz Biotech) (1:400) was added and incubated 1 hour at room temperature. HRP-anti-mouse was used as secondary antibody (Supplementary Figure 1B). To test the cPLA2 antibody performance in human samples, astrocytes lysate (~ 2 μg total protein/lane) and human cortex lysate (10 μg total protein/lane) were loaded into 4–15% TGX gel (Bio-rad). After transferring and blocking, cPLA2 antibody (sc-376,618, Lot J1791, Santa Cruz Biotech) (1:400) was added and incubated overnight at 4 °C. HRP-anti-mouse was used as secondary antibody (Supplementary Figure 1C).

### Cell lysate and brain homogenate preparation

The immortalized or primary astrocytes were lysed with 1x RIPA buffer (Cell Signaling Technology, CST 9806) containing protease inhibitor cocktail (Sigma, P8340) and phosphatase inhibitor cocktail (Sigma, P0044), followed by centrifugation at 14,000 gs for 10 min at 4 °C. The supernatant was collected for further analysis.

The mouse cerebral cortex, human hippocampus, and inferior frontal cortex were weighed, then RIPA buffer containing protease inhibitor cocktail and phosphatase inhibitor cocktail was added as 1:30 (w/v). The tissue was then homogenized using a 2 mL glass Dounce tissue grinder, followed by centrifugation with 14,000 gs for 10 min at 4 °C. The supernatant was collected, and the concentration was measured by BCA kit.

### cPLA2 protein enrichment

To detect the phosphorylated cPLA2 in mouse cortex homogenates, cPLA2 protein was enriched by immunoprecipitation. For each mouse sample, 5 μg of cPLA2 antibody (Santa Cruz Biotechnology, sc-376,618) was conjugated to 50 μL Dynabeads Protein G (Thermo Scientific, 10003D) for 1 hr. at room temperature, then 500 μg total protein in 500 μL RIPA was added to the cPLA2-beads complex and incubated with rotation overnight at 4 °C. The beads were washed with 0.1% PBST 3 times by rotation for 5 min. After washing, 30 μL of 1x sample buffer (Bio-Rad, 1,610,747) was added to the beads and heated for 10 minutes at 100 °C. The supernatant was collected by magnetic force and used for the further Western-blot assay.

### Western blot

The cell lysates, cortex homogenate, and enriched cPLA2 proteins were separated by 4–15% mini-precast protein gels (Bio-Rad, 4,561,086) under reducing conditions and then transferred onto nitrocellulose membranes (Bio-Rad, 1,704,270). After transfer, membranes were blocked with 5% fat-free milk (Bio-Rad, 1,706,404) in TBST for 1 h at room temperature, followed by overnight incubation with the primary antibody in 5% BSA at 4 °C. Then, the membranes were incubated with HRP conjugated secondary antibody for 1 h at room temperature. Chemiluminescent HRP substrate (Millipore, WBKLS0500) was used for detection. Fujifilm LAS-4000 imager system was used to capture images, and the densitometric quantification was done by Gel Quant NET software.

The following antibodies and dilution factors were used: cPLA2 antibody (Santa Cruz Biotechnology, sc-376,618) (1:200), phospho-cPLA2 (Ser505) antibody (CST, 53044) (1:1000), phospho-ERK1/2 antibody (CST, 4370) (1:1000), ERK1/2 antibody (CST, 4595) (1:1000), p38 antibody (CST, 9212) (1:1000), phospho-p38 antibody (CST, 4511) (1:1000), GFAP antibody (CST, 12389) (1:1000), Iba-1 antibody (GeneTex, GTX100042) (1:1000), iNOS antibody (CST, 13120) (1:1000), β-actin antibody (CST, 3700) (1:1000), β-tubulin antibody (CST, 2146) (1:1000), synaptophysin antibody (CST, 36406) (1:1000), Na,K-ATPase antibody (CST, 3010S) (1:1000), ApoE4 antibody (CST, 8941S) (1:1000), HRP-linked anti-mouse IgG (CST, 7076) (1:2000), HRP-linked anti-rabbit IgG (CST, 7074) (1:2000).

### qPCR

The cells and brain specimens were harvested, and RNA was extracted using an RNA extraction kit (Thermo Fisher, K0731). Synthesis of cDNA was done using High-Capacity cDNA Reverse Transcription Kit (Thermo Fisher, 4368814). qPCR was performed using the PowerUp SYBR Green Master Mix (Thermo Fisher, A25742). The following primers were synthesized by Integrated DNA Technologies. The cPLA2 sense (5′-CTGCAAGGCCGAGTGACA-3′) and antisense (5′-TTCGCCCACTTCTCTGCAA-3′); mouse Tnfα sense (5′-GCCTCTTCTCATTCCTGCTTG-3′) and antisense (5′-CTGATGAGAGGGAGGCCATT-3′); mouse Il1β sense (5′-GCAACTGTTCCTGAACTCAACT-3′) and antisense (5′-ATCTTTTGGGGTCCGTCAACT-3′); mouse Il6 sense (5′-TAGTCCTTCCTACCCCAATTTCC-3′) and antisense (5′-TTGGTCCTTAGCCACTCCTTC-3′); mouse Ccl2 sense (5′-GTCCCTGTCATGCTTCTGG-3′) and antisense (5′-GCTCTCCAGCCTACTCATTG-3′); mouse Mip1α sense (5′- TGAAACCAGCAGCCTTTGCTC-3′) and antisense (5′-AGGCATTCAGTTCCAGGTCAGTG-3′); mouse Mip2 sense (5′-ATCCAGAGCTTGAGTGTGACGC-3′) and antisense (5′- AAGGCAAACTTTTTGACCGCC-3′); mouse β-actin sense (5′-ACCTTCTACAATGAGCTGCG-3′) and antisense (5′-CTGGATGGCTACGTACATGG-3′); human TNFα sense (5′-ACTTTGGAGTGATCGGCC-3′) and antisense (5′-GCTTGAGGGTTTGCTACAAC-3′); human IL1β sense (5′-ATGCACCTGTACGATCACTG-3′) and antisense (5′-ACAAAGGACATGGAGAACACC-3′);

human IL6 sense (5′-CCACTCACCTCTTCAGAACG-3′) and antisense (5′-CATCTTTGGAAGGTTCAGGTTG-3′); human CCL2 sense (5′- TGTCCCAAAGAAGCTGTGATC-3′) and antisense (5′-ATTCTTGGGTTGTGGAGTGAG-3′); human GAPDH sense (5′-ACATCGCTCAGACACCATG-3′) and antisense (5′-TGTAGTTGAGGTCAATGAAGGG-3′).

### AA and DHA efflux assays

To investigate arachidonic acid (AA) and docosahexaenoic acid (DHA) release by cPLA2 and iPLA2 activation, respectively, we performed an AA and DHA efflux assay as described previously [2]. ApoE3 and ApoE4 primary astrocytes were seeded at 5000 cells/well in 96-well plates. After 24 h, the culture medium was changed with serum-free DMEM containing fatty acid-free BSA (5 mg/mL) (Sigma, A9647) and ^3^H-AA (1 μCi/mL) or ^14^C-DHA (1 μCi/mL) (Moravek) for 24 h. The cells were then washed twice with 100 μL of DMEM, and 100 μL of DMEM containing BSA (5 mg/mL) was added. After 30 minutes, the medium was removed, and 100 μL of ATP (100 μM) in DMEM without BSA was added. After 15 minutes, the cell culture medium was collected and transferred to scintillation vials filled with 3 mL of scintillation cocktail. The cells were solubilized in 90 μL of NaOH (0.5 N) for 5 minutes, neutralized with 60 μL PBS, and then transferred to scintillation vials filled with 3 mL scintillation cocktail. After rigorous mixing, the vials were counted in a Beckman LS6500 liquid scintillation counter (Beckman Coulter). The efflux of AA and DHA were assessed by the ratio of the corresponding fatty acid in the medium to total (medium and cell lysate). The change of AA and DHA efflux was calculated by subtracting the levels of AA and DHA in the ATP treated group to ATP non-treated group for each genotype. WT primary astrocytes were plated and labeled with ^3^H-AA (1 μCi/mL) or ^14^C-DHA (1 μCi/mL) as described above. Then, the cells were washed twice with 100 μL of DMEM. After wash, 10 μL of DMEM containing BSA and 0.2 μM recombinant ApoE3 or ApoE4 protein were added. After 24 h, the medium was removed, and 100 μL of ATP (100 μM) in DMEM without BSA was added. The AA and DHA efflux were measured as described above after 15 minutes.

### cPLA2 activity assay

cPLA2 activity was detected by the cPLA2 activity assay kit (Cayman Chemical, 765,021). The mouse cortex was homogenized into HEPES buffer (50 mM, pH 7.4, containing 1 mM EDTA) as 1:10 (w/v), and the supernatant was collected after centrifuged and used for cPLA2 activity detection.

### Immunoprecipitation

Immortalized ApoE4 astrocytes were cultured in a 100-mm dish for 18 hours and then were lysed with RIPA containing protease and phosphatase inhibitors. The lysates were used for immunoprecipitation with an anti-cPLA2 antibody or species-matched IgG. After elution, cPAL2 and p38 were detected by Western-blot.

### p38 MAPK inhibition experiment

ApoE4 primary astrocytes were seeded in a 24-wells plate with the intensity of 100,000 cells per well. Forty-eight hours later, cells were pre-treated with p38 MAPK inhibitors – SB202190 (10 μM, Sigma, S7076) or SB203580 (10 μM, Sigma, S8307) in the DMEM culture medium without FBS for 20 minutes, followed by the treatment with vehicle or TNFα (10 ng/mL) (R&D Systems, 210-TA-005) plus IFNγ (100 ng/mL) (Sigma, SRP3058) together for 30 minutes. Then, the cells were lysed with RIPA. Total and phosphorylated cPLA2 and p38 were detected by western-blot.

### LTB4 and PGE2 measurement

For the LTB4 and PGE2 measurements in the human brain samples, brain tissue was weighed, then PBS containing 1 mM EDTA, 10 μM indomethacin (Cox inhibitor, Sigma I8280), and 10 μM NDGA (Lox inhibitor, Sigma 479,975) as 1:10 (w/v) were added. The tissue was then homogenized using a 2 mL glass Dounce tissue grinder, followed by centrifugation with 8000 x g for 10 minutes at 4 °C. The supernatant was collected, and the protein concentration was measured using a BCA kit. LTB4 and PGE2 levels were detected by the assay kit (LTB4 ELISA Kit, Cayman Chemical, 10,009,292; PGE2 ELISA Kit, Cayman Chemical, 500,141).

For the LTB4 measurement in the cells, ApoE3 and ApoE4 primary astrocytes were seeded in a 24-wells plate with the intensity of 100,000 cells per well. Forty-eight hours later, cells were pre-treated with cPLA2 inhibitor-Pyrrophenone (500 nM, Sigma, 5,305,380,001) in the DMEM culture medium without FBS but containing N2 supplement for 30 minutes, followed by the treatment with vehicle or TNFα (10 ng/mL) (R&D Systems, 210-TA-005) plus IFNγ (100 ng/mL) (Sigma, SRP3058) together for 18 hours. Then, the culture media and cell lysate were collected. LTB4 levels were measured in a 4-fold concentrated medium using the assay kit.

ApoE4 primary astrocytes were seeded in a 24-wells plate with the intensity of 100,000 cells per well. Forty-eight hours later, cells were transfected with cPLA2 or non-target (NT) siRNA (10 nM) for 48 hours, followed by the treatment with vehicle or TNFα (10 ng/mL) plus IFNγ (100 ng/mL) together for 24 hours. Then, the culture media and cell lysate were collected. LTB4 levels were measured in a 4-fold concentrated medium by the assay kit.

### ROS measurement

ROS were detected by the DCFDA cellular ROS detection assay kit (Abcam, ab113851). ApoE3 and ApoE4 primary astrocytes were seeded in dark, clear bottom 96-wells plate with the intensity of 20,000 cells per well. Forty-eight hours later, cells were pre-treated with cPLA2 inhibitor (1 μM) in the DMEM culture medium without FBS but containing N2 supplement for 30 minutes, followed by the treatment with vehicle or TNFα (10 ng/mL) plus IFNγ (100 ng/mL) together for 24 hours. After removing the media and washing plate once with 1x assay buffer, the cells were stained with DCFDA solution (100 μL/well) for 45 minutes at 37 °C in the dark. Then, the DCFDA solution was removed, and the 1x assay buffer (100 μL/well) was added to the plate. ROS levels were measured using a fluorescent plate reader at Excision/Emission = 485/585 nm.

### Assessment of cellular distribution of cPLA2 in synaptosomes

Synaptosomes prepared from postmortem human frontal cortices using an established method with minor modification [61]. Briefly, the postmortem human frontal cortical tissue (about 100 mg) were homogenized in 10 volume (w:v) of ice-cold homogenization buffer (10 mM HEPES, pH 7.4, 0.32 M sucrose, 0.1 mM EDTA containing EDTA-free protease inhibitor cocktail (Roche, 04693159001) and 0.2% 2-mercaptoethanol) using a Teflon/glass homogenizer (10 strokes). The homogenates were cleared by centrifugation (1000 x g for 10 min), and the supernatants were centrifuged at 15,000 x g at 4 °C for 30 min to pellet the synaptosomes (P2 fraction). The synaptosomes were washed twice at 4 °C in 1 mL of ice-cold oxygenated KR (Kreb’s-Ringer) solution (25 mM HEPES, pH 7.4, 118 mM NaCl, 4.8 mM KCl, 25 mM NaHCO3, 1.3 mM CaCl2, 1.2 mM MgSO4, 1.2 mM KH2PO4, 10 mM glucose, 100 μM ascorbic acid, EDTA-free protease inhibitor cocktail). The synaptosomes were then resuspended in 1 mL of K-R solution, and the protein concentrations were determined by the BCA kit. Two hundred μg synaptosomes were incubated with 0.1 μM of Aβ42, rE3, rE4 or 0.7 μM Ceramide-1-phosphate (C-1-P) (Sigma, C4832) in 200 μL oxygenated KR buffer for 30 min at 37 °C followed by incubation with 2.5 μM C-1-P for 15 min. Upon completion of incubation, an ice-cold protein phosphatase inhibitor cocktail (Roche, 04906837001) was added and placed on ice for 5 min, and synaptosomes were pelleted by centrifugation.

The cytosolic and membranous fractions of the synaptosomes were isolated by centrifugation. The synaptosomes were briefly sonicated (Kontes Micro Cell Disrupter) in 500 μL of immunoprecipitation buffer (25 mM HEPES, pH 7.5, 200 mM NaCl, 1 mM EDTA, protease and protein phosphatase inhibitor cocktails, and 0.02% 2-mercaptoethanol and centrifuged at 48,000 x g for 15 min at at 4 °C. The resultant supernatant was collected as the cytosolic fraction, and the pellet was dissolved in 500 μL immunoprecipitation buffer containing 0.5% digitonin, 0.2% sodium cholate, and 0.5% NP-40 and incubated at 4 °C with end-to-end shaking for 1 h. Then, the pellet was centrifuged again at 48,000 x g for 15 min at 4 °C to collect the supernatant as the membrane fraction. cPLA2 were isolated by immunoprecipitation with 1 h incubation at 4 °C with anti-cPLA2 antibodies (Santa Cruz Biotechnology, sc-376,636 and sc-454) and followed by overnight incubation with Dynabeads Protein G (Thermo Scientific, 10004D). After three washes with 1 mL of ice-cold 0.1% TBST, 25 μL diluted (1.5x) sample buffer (Bio-rad, 1,610,747) was added to the beads and boiled for 5 min at 95 °C. cPLA2 protein expression was determined by western-blot imaging with anti-cPLA2 (Santa Cruz Biotechnology, sc-376,618) antibody. Beta-actin and Na,K ATPase were used as markers for loading control.

### LDH assay in synaptosomes

To assess synaptosomes membrane integrity, we performed a LDH activity test (Thermo Fisher, C20300) [28]. The synaptosomes were isolated and incubated for 1 hour at different conditions. Then, 50 μL of synaptosome in KR buffer with 1% Triton X-100 (total LDH) or without Triton X-100 (free LDH) were incubated with 50 μL LDH reaction mixture for 30 minutes. After adding the stop solution, the absorbance at 490 nm and 680 nm were measured. The LDH activity was indicated as the absorbance at 490–680 nm.

### Statistical analysis

Descriptive results are presented as the mean ± SEM. Data were analyzed using two-tailed Student’s t-test or two-way ANOVA. Statistical significance was present at *p* < 0.05.

## Supplementary Information

**Additional file 1: Supplementary Figure 1.** Validation of cPLA2 and p-cPLA2 antibodies. **A**, cPLA2 antibodies from Santa Cruz Biotech (sc-376,636, sc-376,618 and sc-454, 1:400) and Sigma (SAB4502200, 1:1000) were tested in astrocytes transfected with cPLA2 siRNA. Total cPLA2 intensity was reduced after cPLA2 siRNA treatment. **B,** Phospho-cPLA2 (p-cPLA2) antibody (#53044, Cell Signaling Technology) was validated with greater band intensity in the astrocytes treated with ATP. After imaging, the membrane was stripped and blotted with anti-cPLA2 antibody (sc-376,618, Santa Cruz Biotech) revealing that total cPLA2 did not differ after ATP treatment. The relative amount of p-cPLA2 to total cPLA2 was greater in the ATP treatment condition. **C**, cPLA2 antibody performance in human samples. Human cortex or astrocytes lysates were loaded into the gel and blotted with cPLA2 antibody (sc-376,618, Santa Cruz Biotech).

**Additional file 2: Supplementary Figure 2.** ApoE4 increases cPLA2 expression in immortalized ApoE astrocytic cultures. **A**, cPLA2 mRNA levels in immortalized ApoE3 or ApoE4 astrocytes (*n* = 3 for each genotype). **B**, cPLA2 and phosphorylated cPLA2 (p-cPLA2) protein levels in immortalized ApoE3 or ApoE4 astrocytes were detected by western blot (*n* = 3 for each genotype). **C**, cPLA2 and phosphorylated cPLA2 (p-cPLA2) protein levels in primary microglial cells from ApoE3 or ApoE4-TR mice were detected western blot (*n* = 2 for each genotype). Data are represented as mean ± SEM and analyzed by Student’s t-test (two-tailed).

**Additional file 3: Supplementary Figure 3.** cPLA2 distribution in cytosol and membrane of primary astrocytes. ApoE3 and ApoE4 primary astrocytes were labeled with biotin, and the membrane proteins were purified with Avidin agarose beads. Phosphorylated and total cPLA2 levels were detected by western blot. Beta-actin was used as the loading control for cytosolic fraction, and Na,K ATPase, was the loading control for the membranous fraction.

**Additional file 4: Supplementary Figure 4.** Aβ and APP levels in the frontal cortex of persons with AD dementia with different *APOE* genotypes. Aβ and APP protein levels in the inferior frontal cortex from AD patients were detected by western blot (*n* = 12 for AD E3/E3; *n* = 10 for AD E3/E4). The lysate of astrocytes treated with Aβ42 was used as positive control. Data are represented as mean ± SEM and analyzed by Student’s t-test (two-tailed).

**Additional file 5: Supplementary Figure 5.** Total and activated cPLA2 and p38 levels in the hippocampus of persons with different APOE genotypes and disease conditions. Frozen hippocampus from persons with NCI or AD dementia with different *APOE* genotypes were homogenized with RIPA buffer. **(A)** Phosphorylated-cPLA2 and total cPLA2 protein levels and **(B)** phosphorylated-p38 and total p38 protein levels were detected by western blot (*n* = 6 for each group). Data are represented as mean ± SEM and analyzed by Student’s t-test (two-tailed).

**Additional file 6: Supplementary Figure 6.** A, Correlation of p-cPLA2 levels with GFAP levels in the inferior frontal cortex from persons with AD dementia. **B**, Correlation of p-cPLA2 levels with Iba1 levels in the inferior frontal cortex from persons with AD dementia. The linear regression from GraphPad Prism 9 was used to measure of association. **C**, Correlation of p-cPLA2 levels in the inferior frontal cortex with the Braak stage of persons with AD dementia. A multiple linear regression analysis was used for comparisons. Student’s t-test (two-tailed) was also used as indicated.

**Additional file 7: Supplementary Table 1.**

**Additional file 8.**

## Data Availability

All data used and analyzed for the current study are available from the corresponding author on reasonable request.

## Abbreviations

ApoE: Apolipoprotein E
ApoE-TR: ApoE-targeted replacement
AD: Alzheimer disease
NCI: No cognitive impairment
cPLA2: Calcium-dependent cytosolic phospholipase A2
iPLA2: Calcium-independent phospholipase A2 (iPLA2)
DHA: Docosahexaenoic acid
AA: Arachidonic acid
LPC: Lysophosphatidylcholine
LTB4: Leukotriene B4
PGE2: Prostaglandin E2
ROS: Reactive oxygen species
iNOS: Inducible nitric oxide synthase
NO: Nitric oxide
COX: Cyclooxygenase
LOX: Lipoxygenase
MAPK: Mitogen-activated protein kinase
MAVS: Mitochondrial antiviral-signaling protein
NF-kB: Nuclear factor kappa-light-chain-enhancer of activated B cells
LPS: Lipopolysaccharide
CRP: C reactive protein
