# Supplementary figures and images for: Calcium-dependent cytosolic phospholipase A2 activation is implicated in neuroinflammation and oxidative stress associated with ApoE4
Source: Mol Neurodegener. 2022 Jun 15;17:42. doi: 10.1186/s13024-022-00549-5 (PMC9202185; doi:10.1186/s13024-022-00549-5)

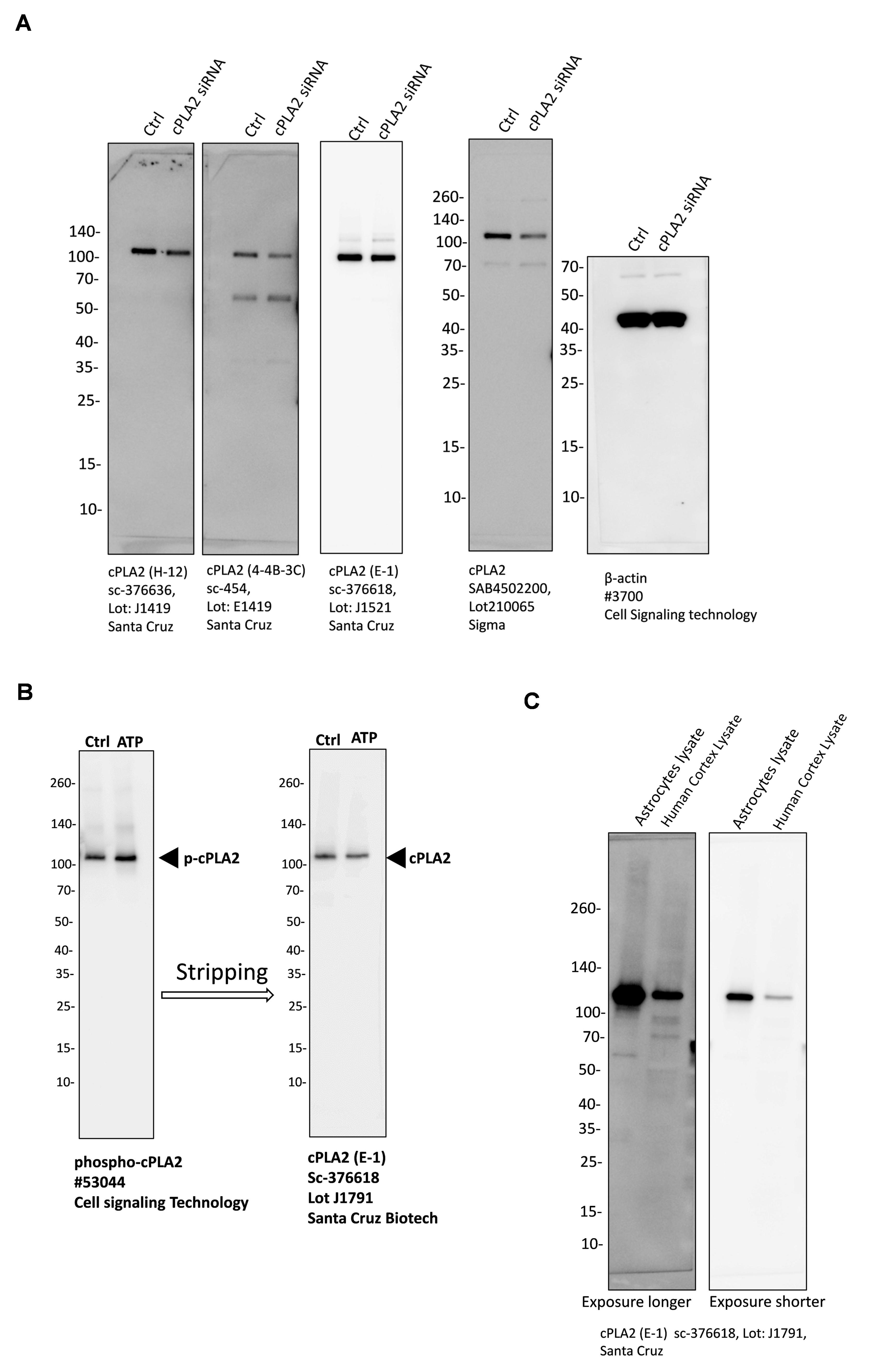

Supplement: Supplementary file 1 — Additional file 1: Supplementary Figure 1. Validation of cPLA2 and p-cPLA2 antibodies. A, cPLA2 antibodies from Santa Cruz Biotech (sc-376,636, sc-376,618 and sc-454, 1:400) and Sigma (SAB4502200, 1:1000) were tested in astrocytes transfected with cPLA2 siRNA. Total cPLA2 intensity was reduced after cPLA2 siRNA treatment. B, Phospho-cPLA2 (p-cPLA2) antibody (#53044, Cell Signaling Technology) was validated with greater band intensity in the astrocytes treated with ATP. After imaging, the membrane was stripped and blotted with anti-cPLA2 antibody (sc-376,618, Santa Cruz Biotech) revealing that total cPLA2 did not differ after ATP treatment. The relative amount of p-cPLA2 to total cPLA2 was greater in the ATP treatment condition. C, cPLA2 antibody performance in human samples. Human cortex or astrocytes lysates were loaded into the gel and blotted with cPLA2 antibody (sc-376,618, Santa Cruz Biotech). [file 13024_2022_549_MOESM1_ESM.tif]

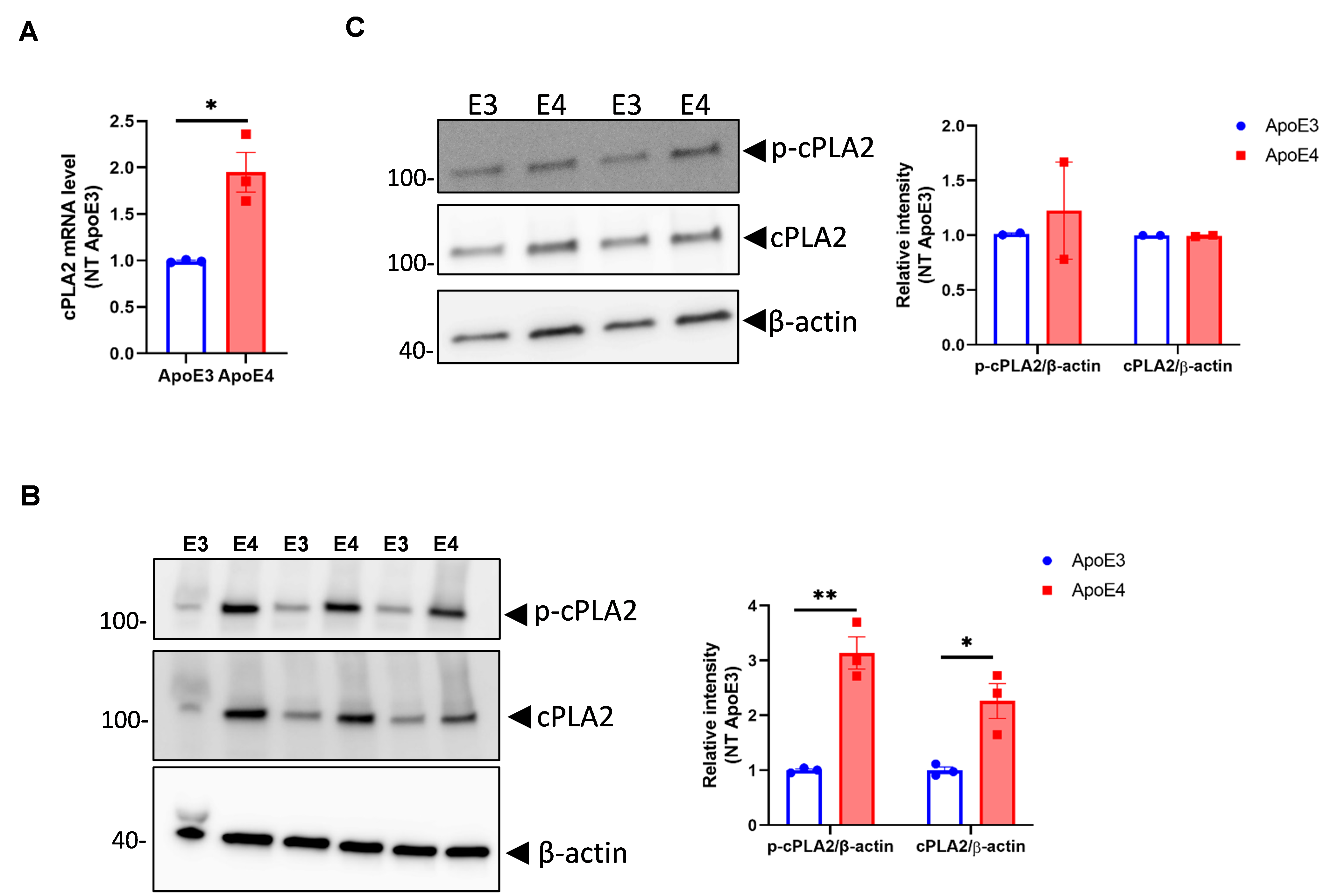

Supplement: Supplementary file 2 — Additional file 2: Supplementary Figure 2. ApoE4 increases cPLA2 expression in immortalized ApoE astrocytic cultures. A, cPLA2 mRNA levels in immortalized ApoE3 or ApoE4 astrocytes (n = 3 for each genotype). B, cPLA2 and phosphorylated cPLA2 (p-cPLA2) protein levels in immortalized ApoE3 or ApoE4 astrocytes were detected by western blot (n = 3 for each genotype). C, cPLA2 and phosphorylated cPLA2 (p-cPLA2) protein levels in primary microglial cells from ApoE3 or ApoE4-TR mice were detected western blot (n = 2 for each genotype). Data are represented as mean ± SEM and analyzed by Student’s t-test (two-tailed). [file 13024_2022_549_MOESM2_ESM.tif]

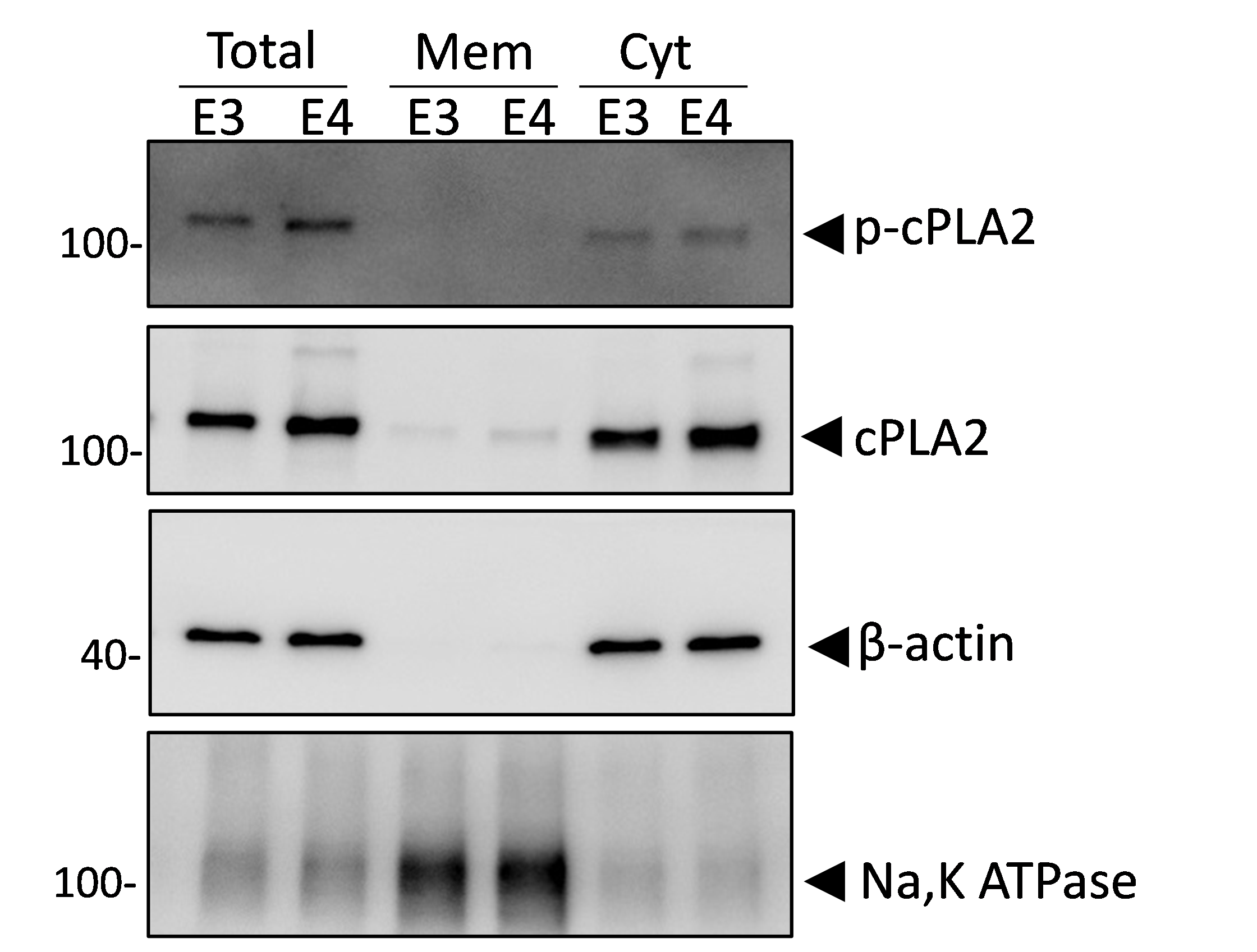

Supplement: Supplementary file 3 — Additional file 3: Supplementary Figure 3. cPLA2 distribution in cytosol and membrane of primary astrocytes. ApoE3 and ApoE4 primary astrocytes were labeled with biotin, and the membrane proteins were purified with Avidin agarose beads. Phosphorylated and total cPLA2 levels were detected by western blot. Beta-actin was used as the loading control for cytosolic fraction, and Na,K ATPase, was the loading control for the membranous fraction. [file 13024_2022_549_MOESM3_ESM.tif]

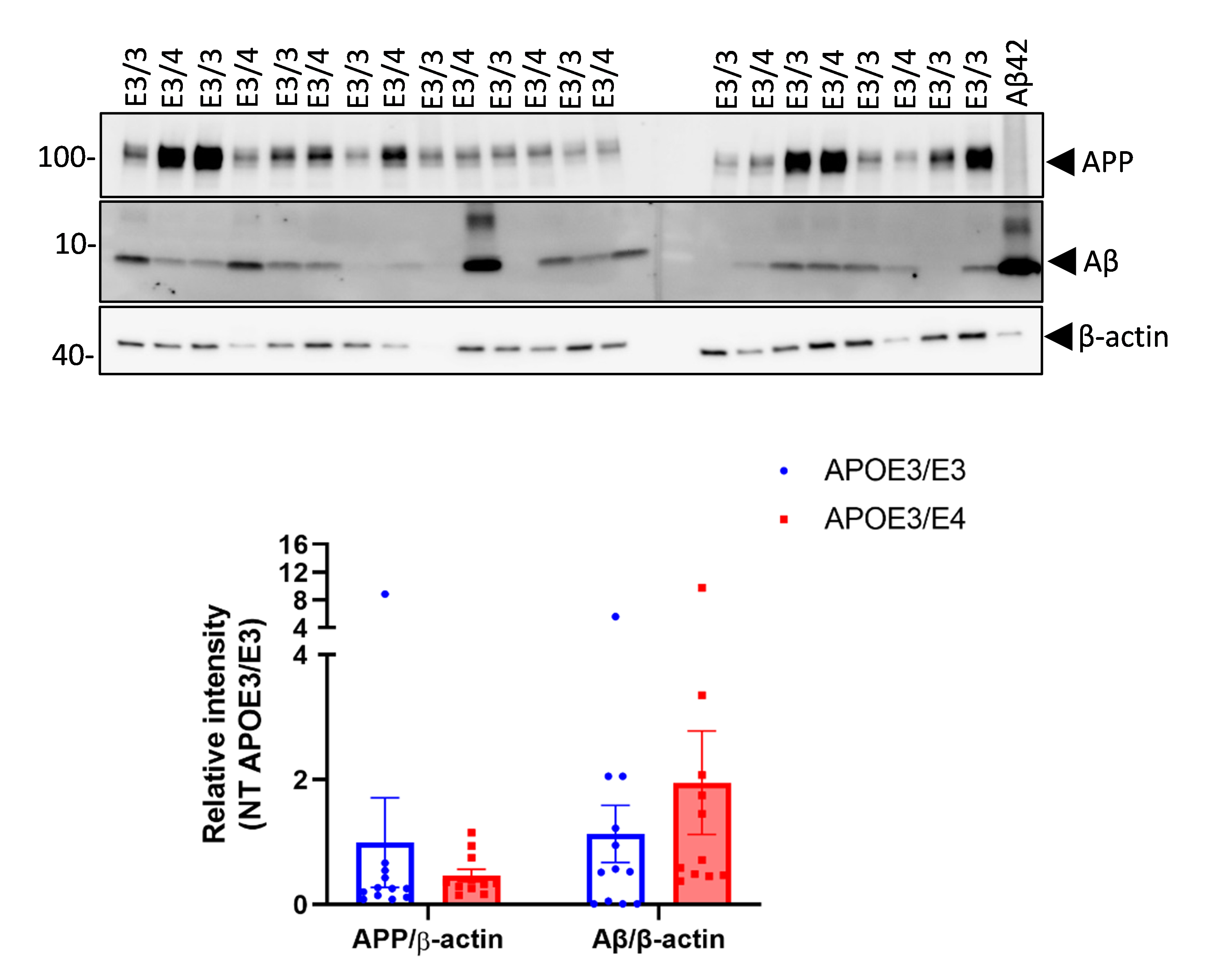

Supplement: Supplementary file 4 — Additional file 4: Supplementary Figure 4. Aβ and APP levels in the frontal cortex of persons with AD dementia with different APOE genotypes. Aβ and APP protein levels in the inferior frontal cortex from AD patients were detected by western blot (n = 12 for AD E3/E3; n = 10 for AD E3/E4). The lysate of astrocytes treated with Aβ42 was used as positive control. Data are represented as mean ± SEM and analyzed by Student’s t-test (two-tailed). [file 13024_2022_549_MOESM4_ESM.tif]

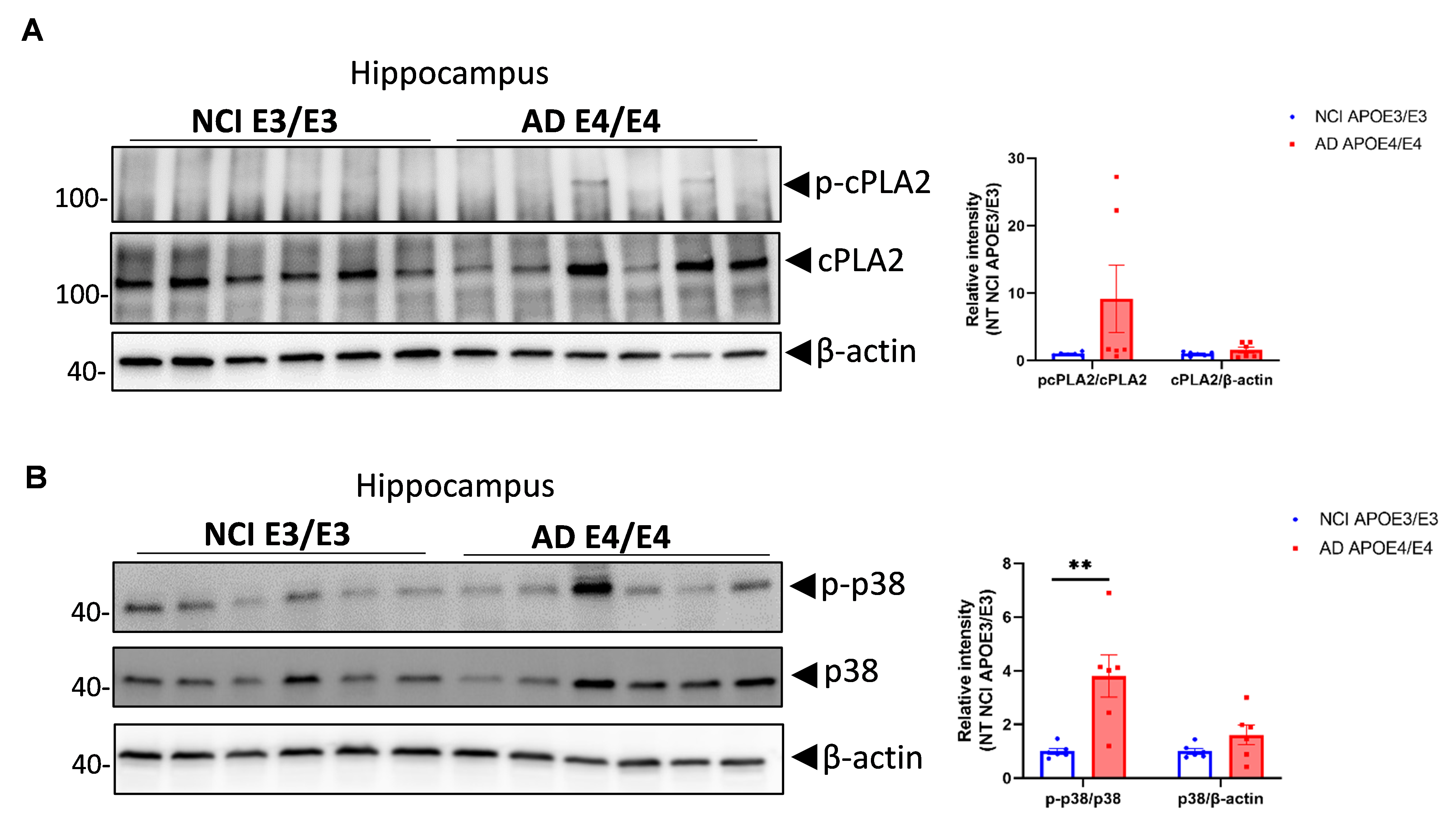

Supplement: Supplementary file 5 — Additional file 5: Supplementary Figure 5. Total and activated cPLA2 and p38 levels in the hippocampus of persons with different APOE genotypes and disease conditions. Frozen hippocampus from persons with NCI or AD dementia with different APOE genotypes were homogenized with RIPA buffer. (A) Phosphorylated-cPLA2 and total cPLA2 protein levels and (B) phosphorylated-p38 and total p38 protein levels were detected by western blot (n = 6 for each group). Data are represented as mean ± SEM and analyzed by Student’s t-test (two-tailed). [file 13024_2022_549_MOESM5_ESM.tif]

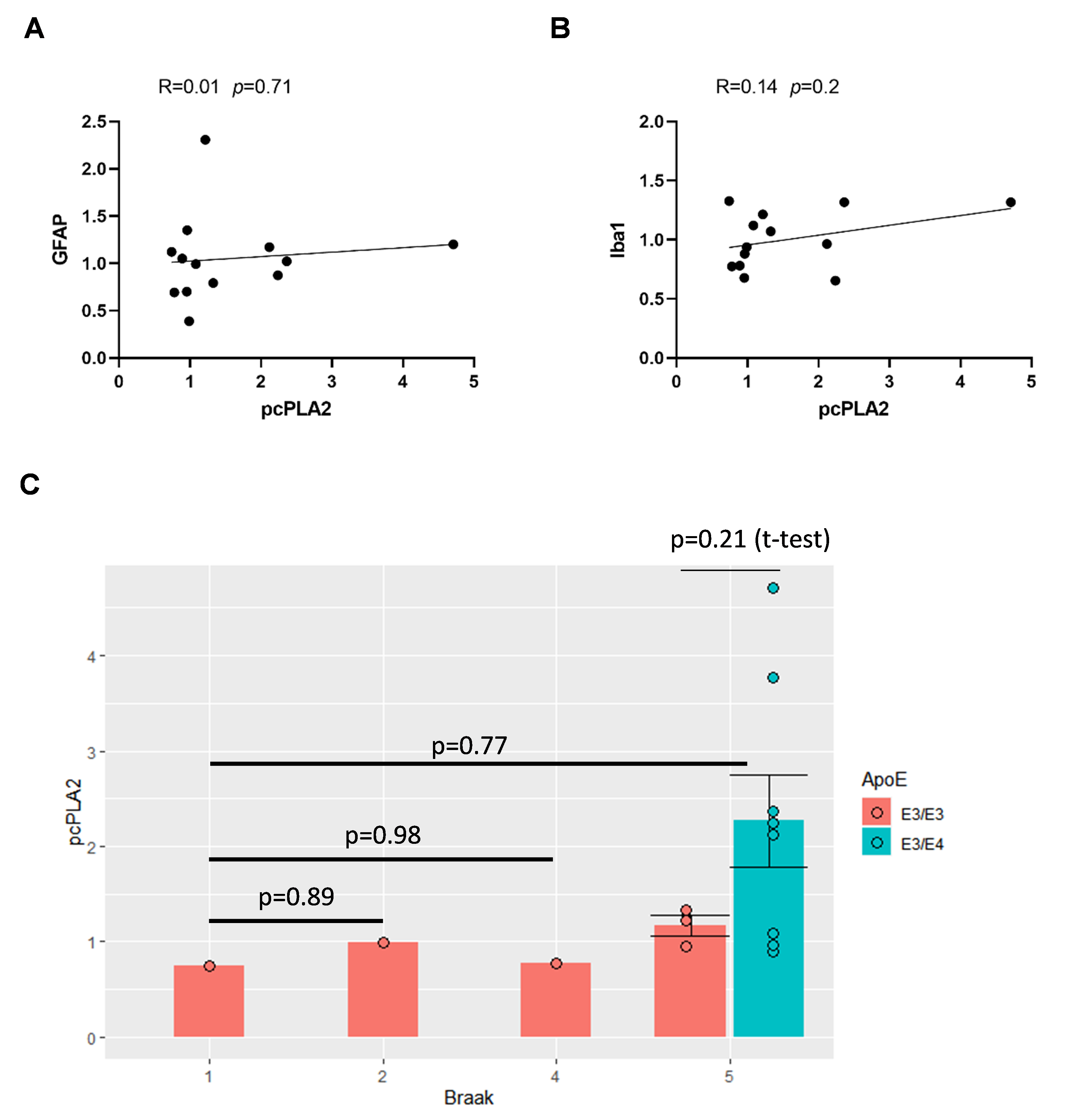

Supplement: Supplementary file 6 — Additional file 6: Supplementary Figure 6. A, Correlation of p-cPLA2 levels with GFAP levels in the inferior frontal cortex from persons with AD dementia. B, Correlation of p-cPLA2 levels with Iba1 levels in the inferior frontal cortex from persons with AD dementia. The linear regression from GraphPad Prism 9 was used to measure of association. C, Correlation of p-cPLA2 levels in the inferior frontal cortex with the Braak stage of persons with AD dementia. A multiple linear regression analysis was used for comparisons. Student’s t-test (two-tailed) was also used as indicated. [file 13024_2022_549_MOESM6_ESM.tif]
