# Supplementary material for: Calcium-dependent cytosolic phospholipase A2 activation is implicated in neuroinflammation and oxidative stress associated with ApoE4
Source: Mol Neurodegener. 2022 Jun 15;17:42. doi: 10.1186/s13024-022-00549-5 (PMC9202185; doi:10.1186/s13024-022-00549-5)
Supplement: Supplementary file 7 — Additional file 7: Supplementary Table 1. [file 13024_2022_549_MOESM7_ESM.docx]

**Supplementary Table 1.**

| Regions sampled and source | Hippocampus, USC ADRC | |
| --- | --- | --- |
| Clinical diagnosis | NCI | AD |
| Genotype | E3/E3 | E4/E4 |
| Sample size, n | 6 | 6 |
| Age (years ± SD) * | 85±5 | 75±5 |
| Sex (n, female/male) * | 5/1 | 4/2 |
| Braak stage | I | V |
