# Supplementary material for: Calcium-dependent cytosolic phospholipase A2 activation is implicated in neuroinflammation and oxidative stress associated with ApoE4
Source: Mol Neurodegener. 2022 Jun 15;17:42. doi: 10.1186/s13024-022-00549-5 (PMC9202185; doi:10.1186/s13024-022-00549-5)

Fig 1B

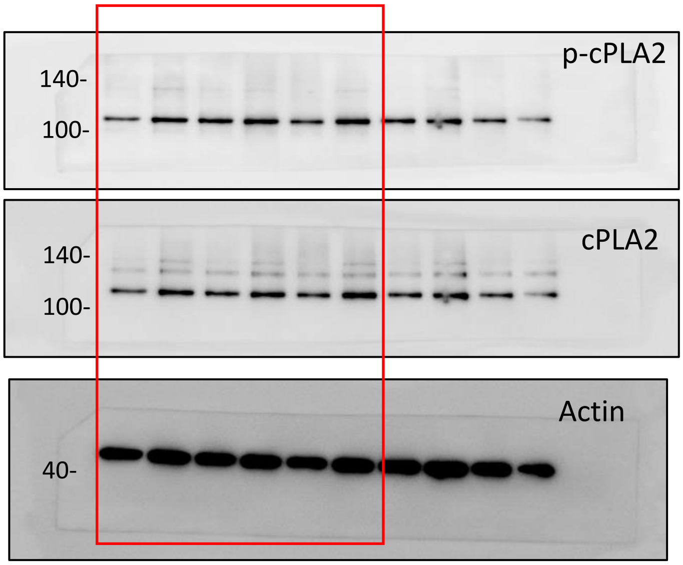

Fig. 1D

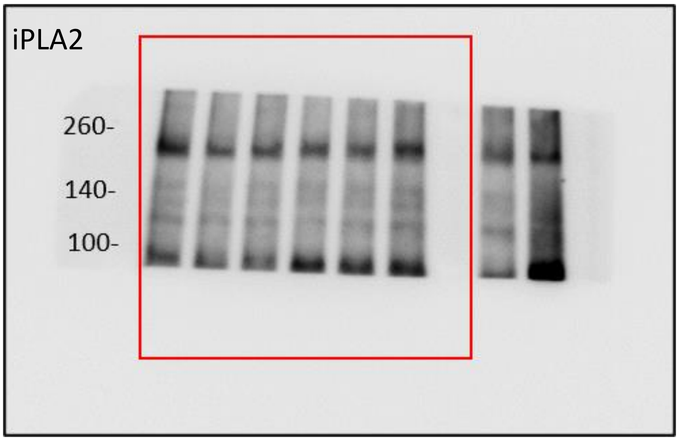

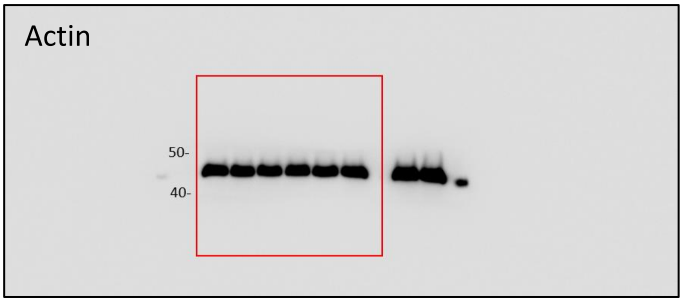

Fig. 2B

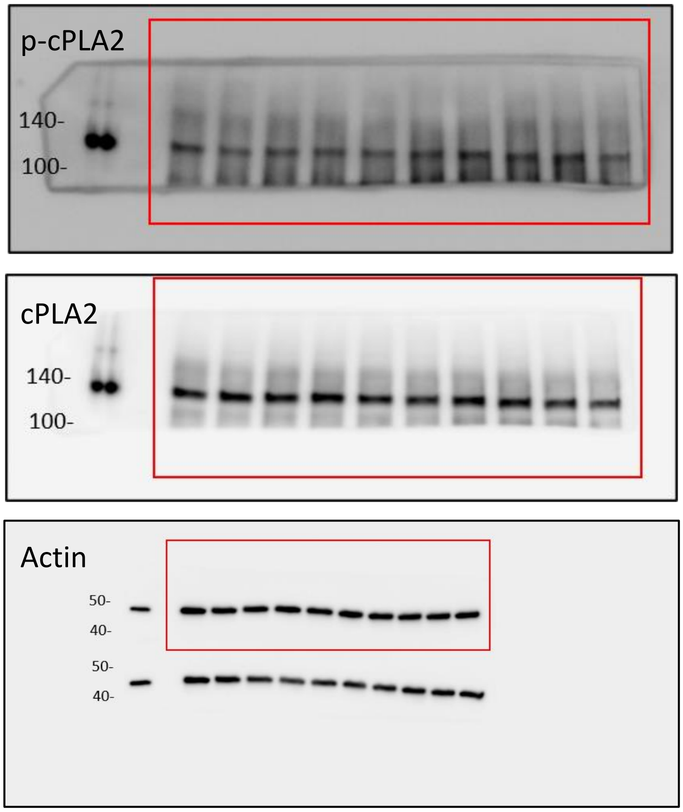

Fig. 3A

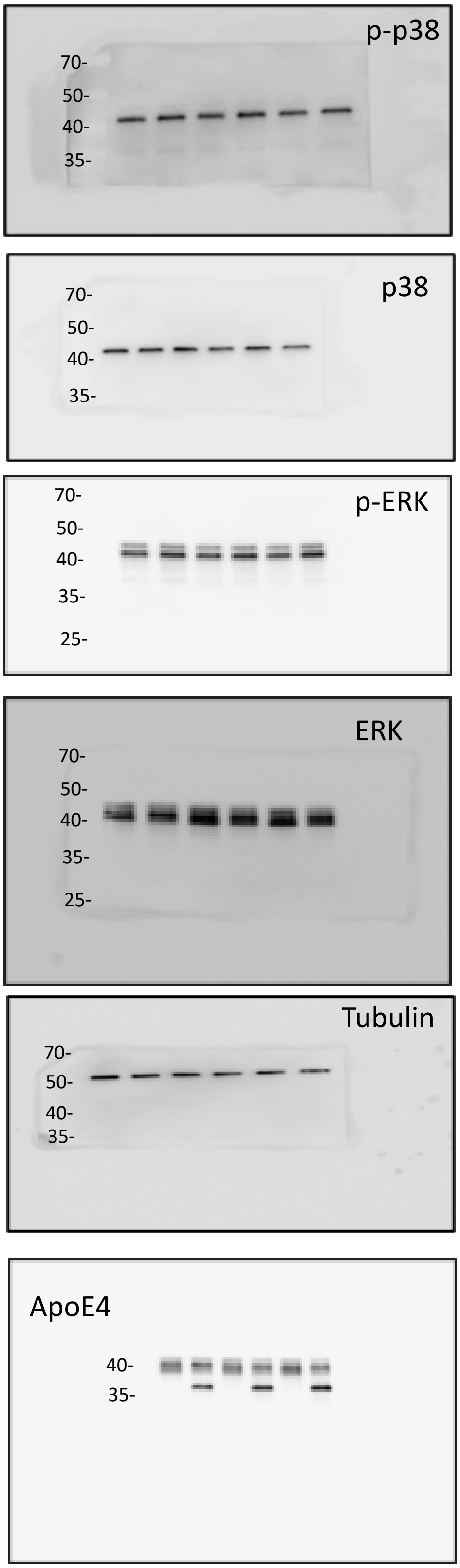

p-p38
70-
50-
40-
35-
p38
70-
50-
40-
35-
p-ERK
70-
50-
40-
35-
25-
ERK
70-
50-
40-
35-
25-
Tubulin
70-
50-
40-
35-
ApoE4
40-
35-

Fig. 3B

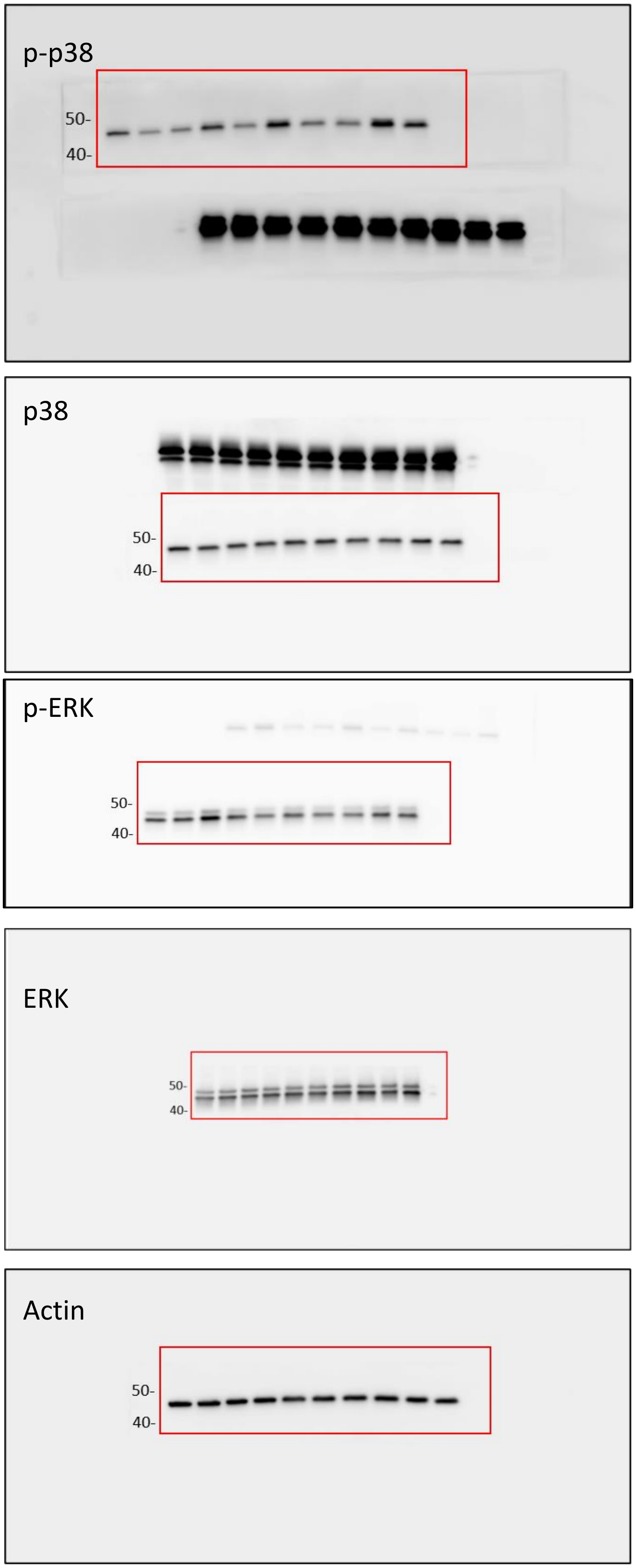

p-p38
50-
40-
p38
50-
40-
p-ERK
50-
40-
ERK
50-
40-
Actin
50-
40-

Figure 3C Repeats

1st

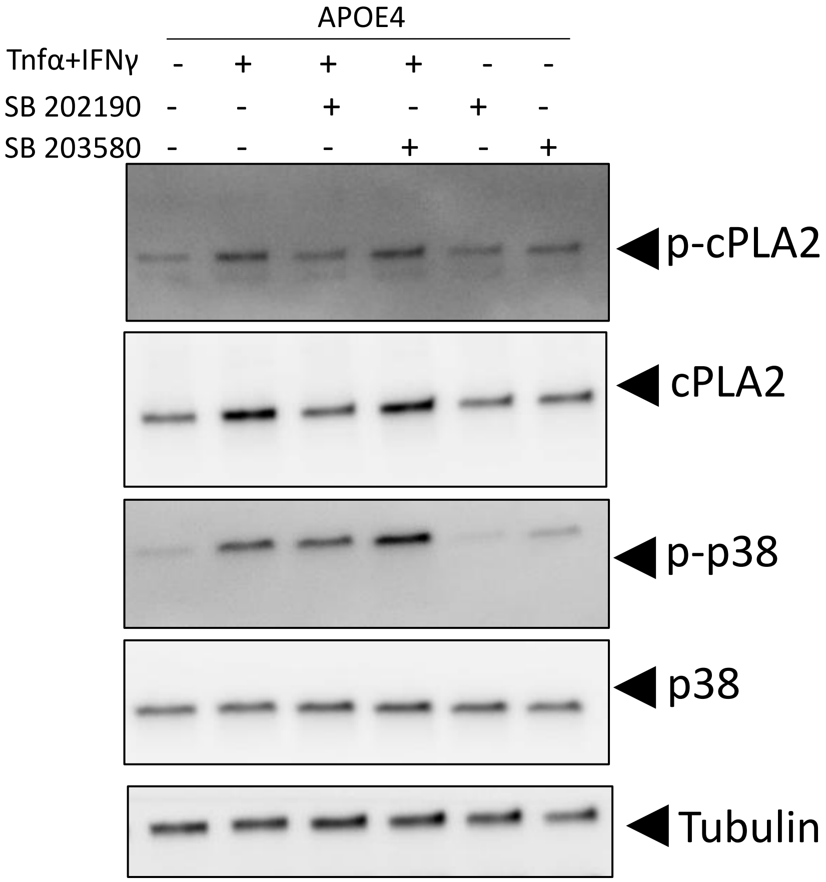

2nd

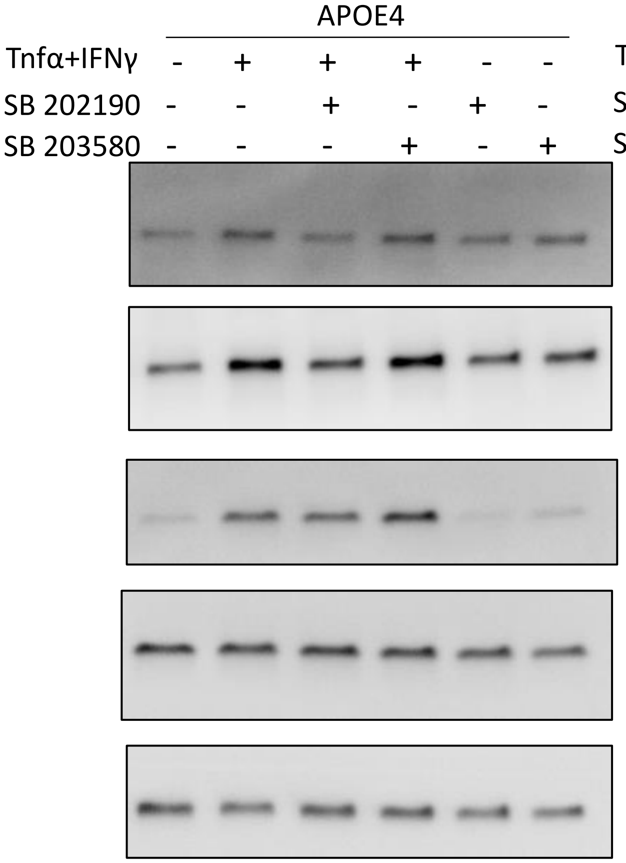

3rd

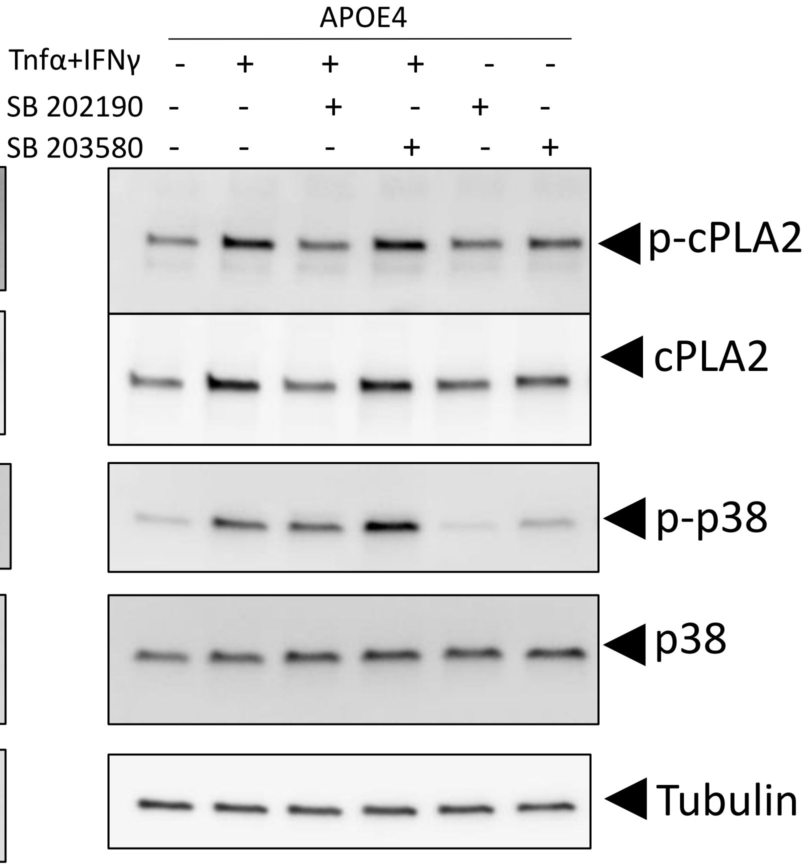

Figure 3C Repeats

Full blot 1st

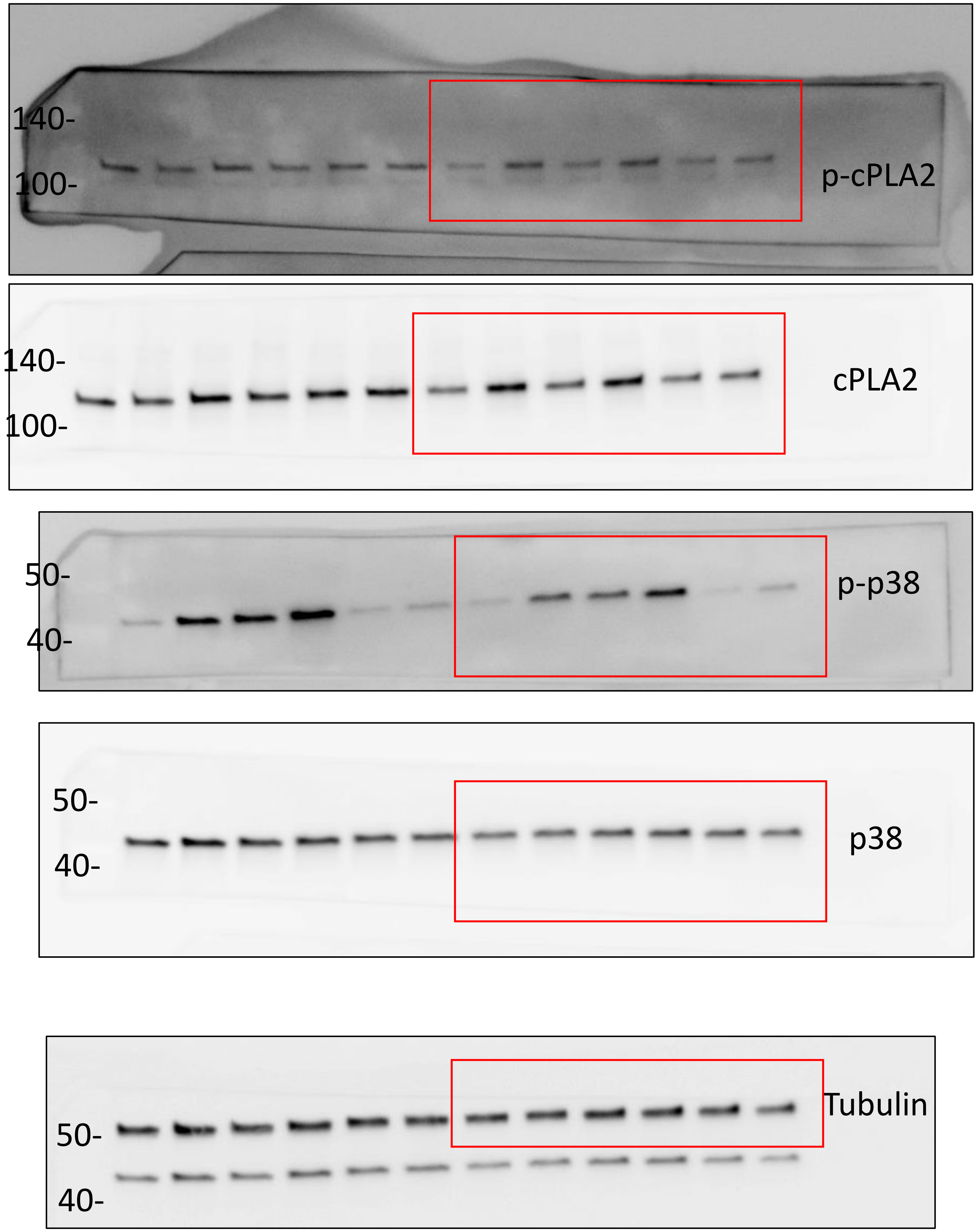

Figure 3C Repeats

Full blot 2nd

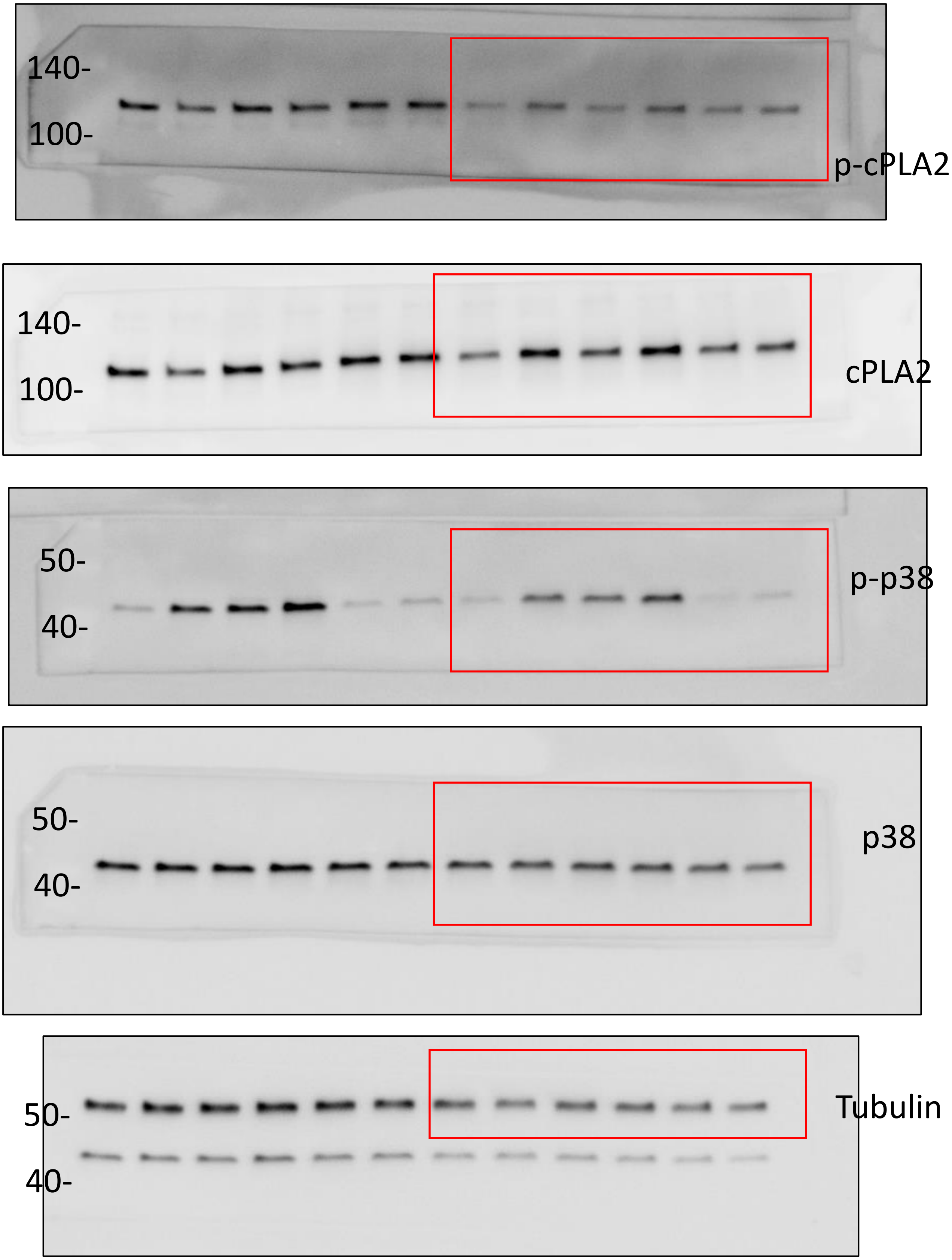

Fig. 3C

Full blot 3rd

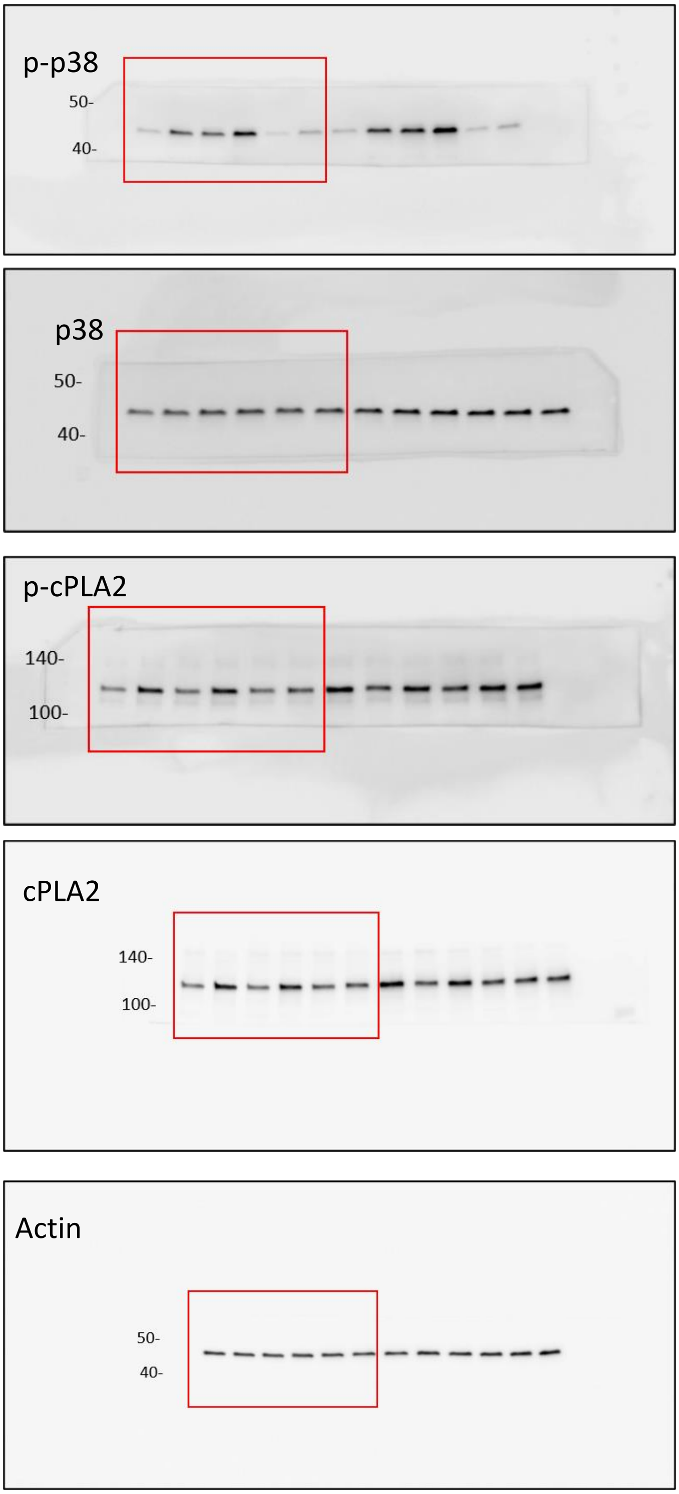

Fig. 3D

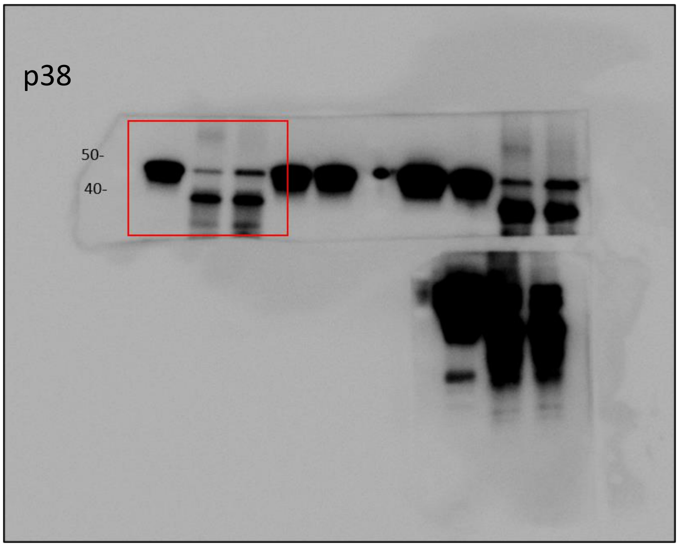

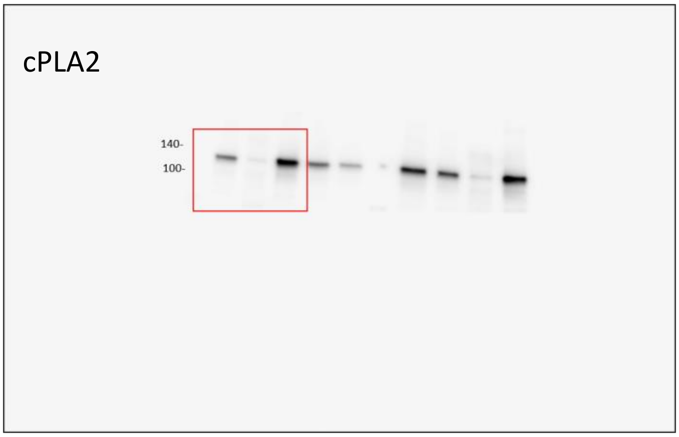

Fig. 4A

p-cPLA2

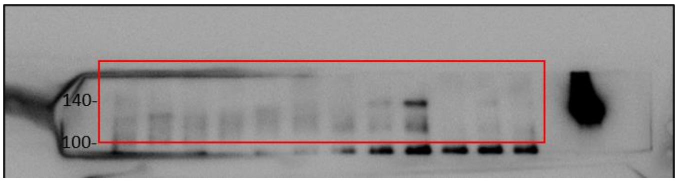

cPLA2

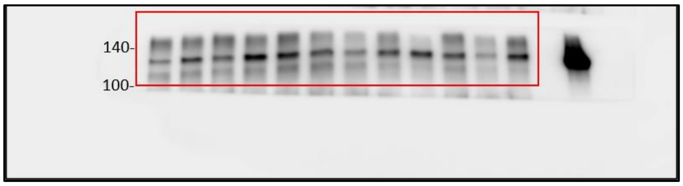

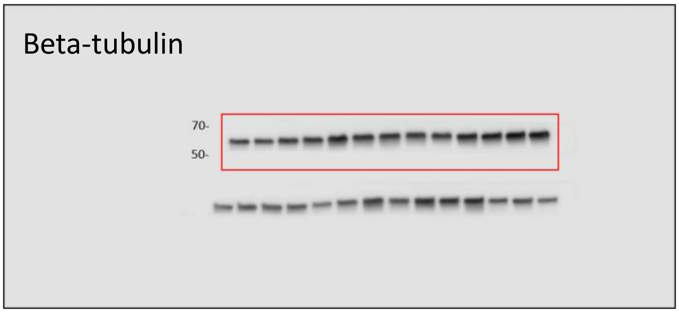

Fig. 4B

p-cPLA2

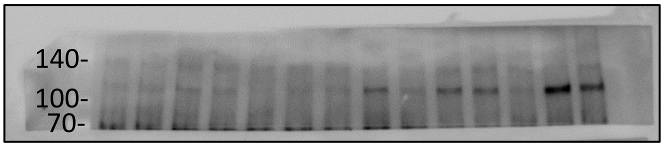

cPLA2

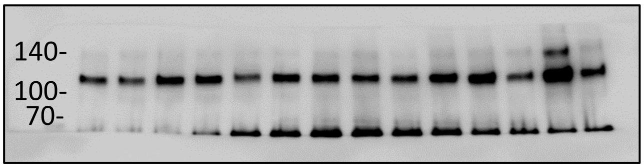

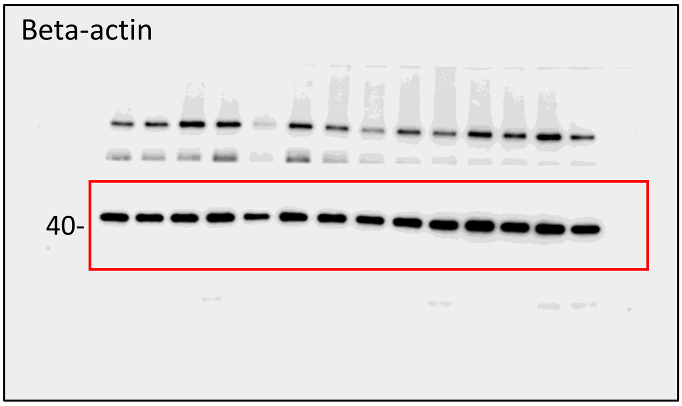

Fig. 5A

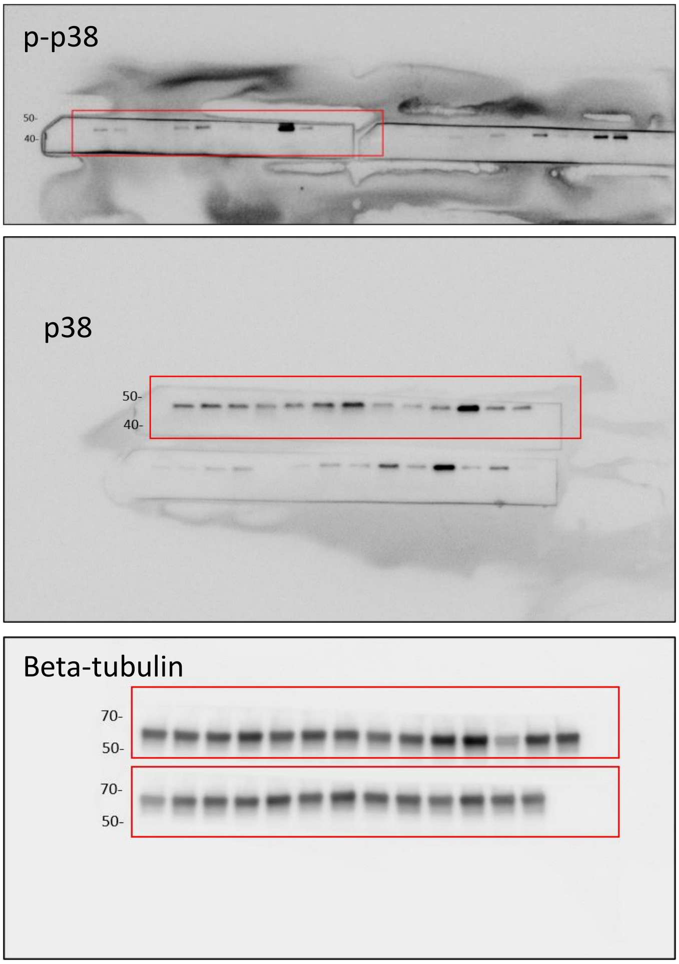

Fig. 5B

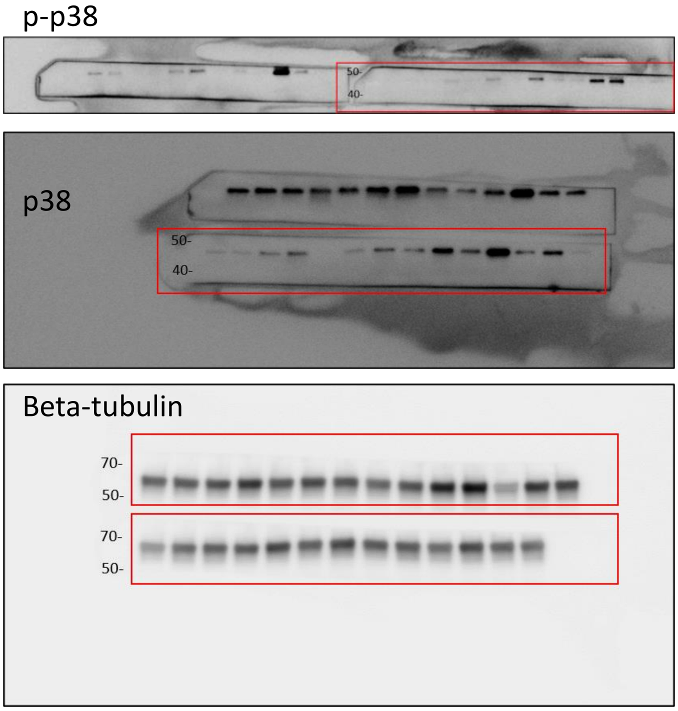

Fig. 6E

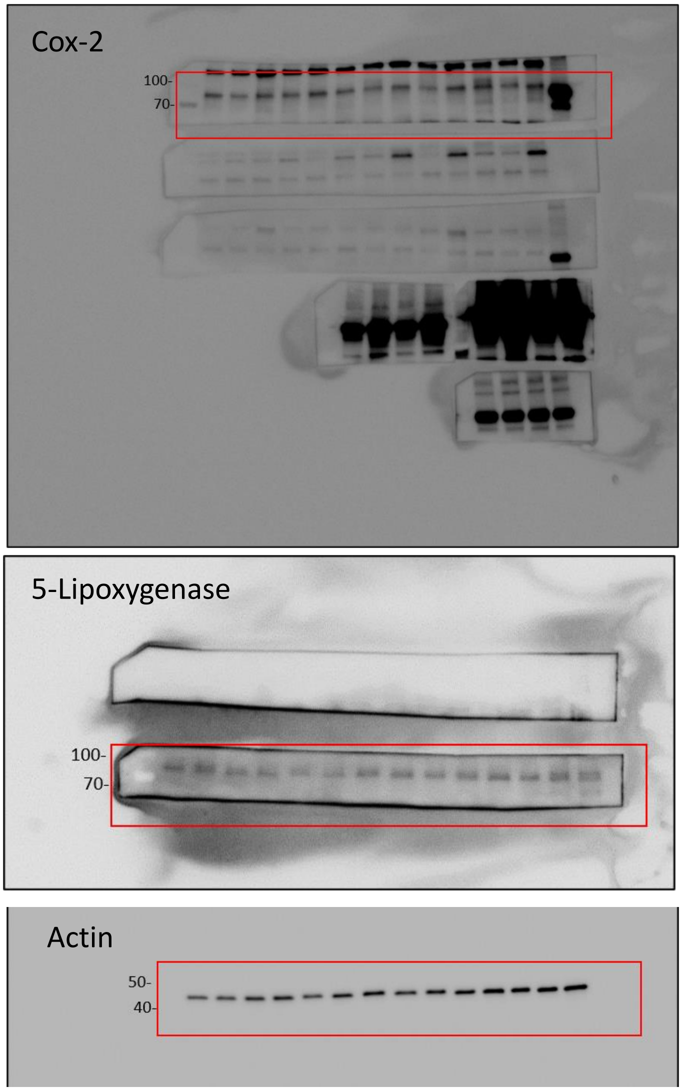

Fig. 7C

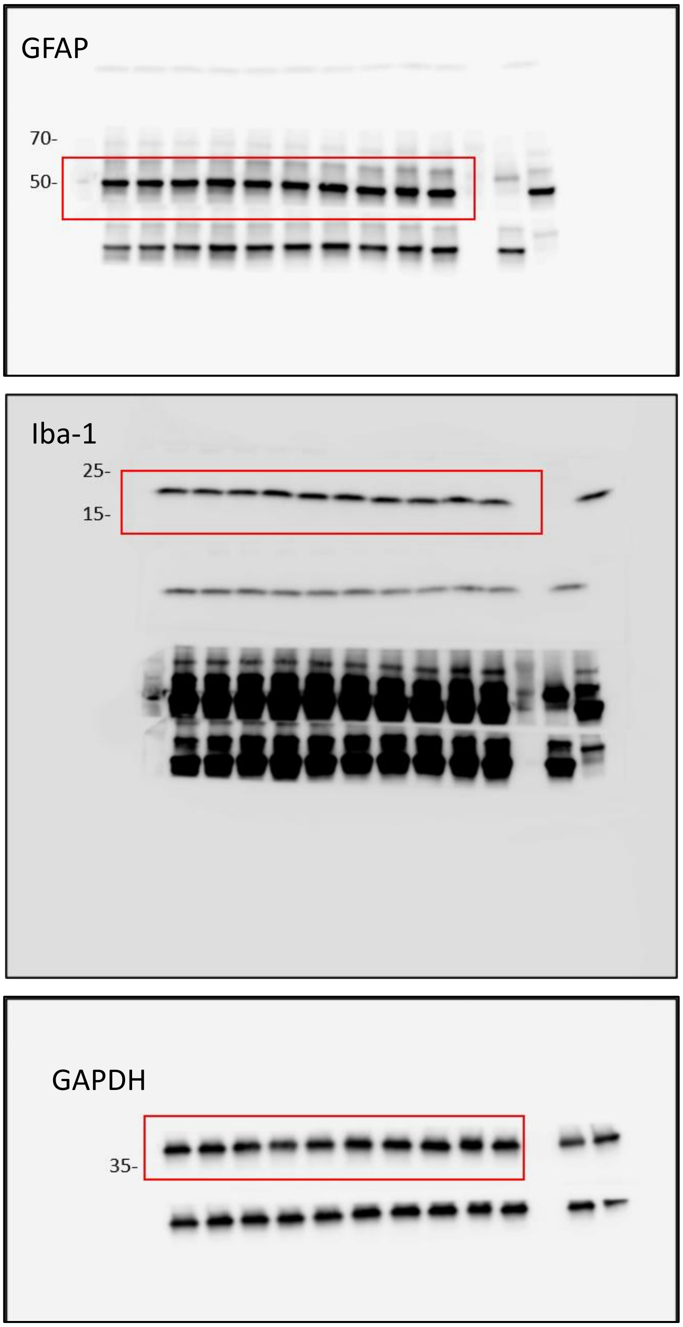
GFAP
70-
50-
Iba-1
25-
15-
GAPDH
35-

Fig. 7E

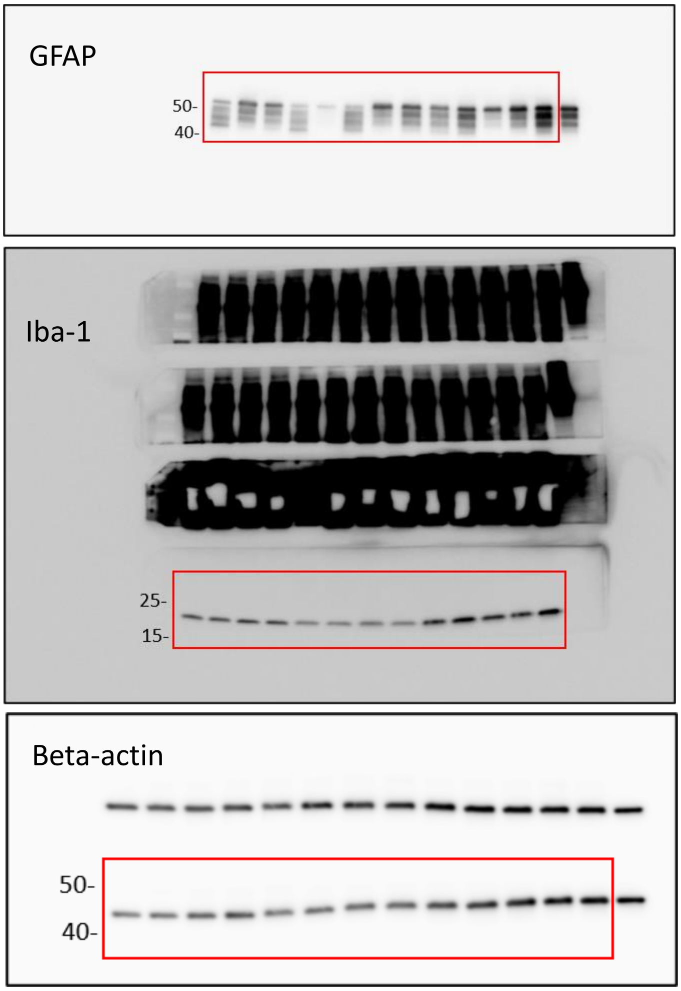
GFAP
50-
40-
Iba-1
25-
15-
Beta-actin
50-
40-

Figure 8A Repeats

1st

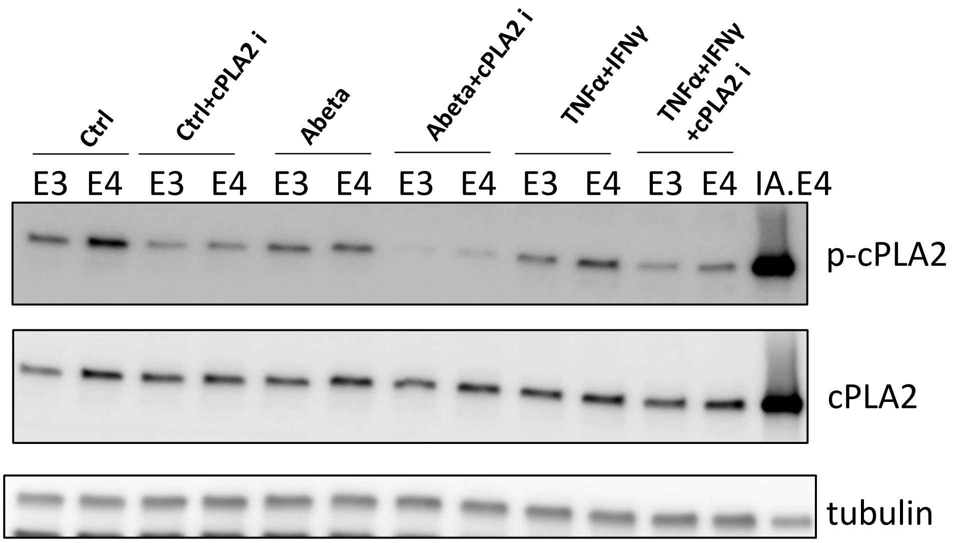

2nd

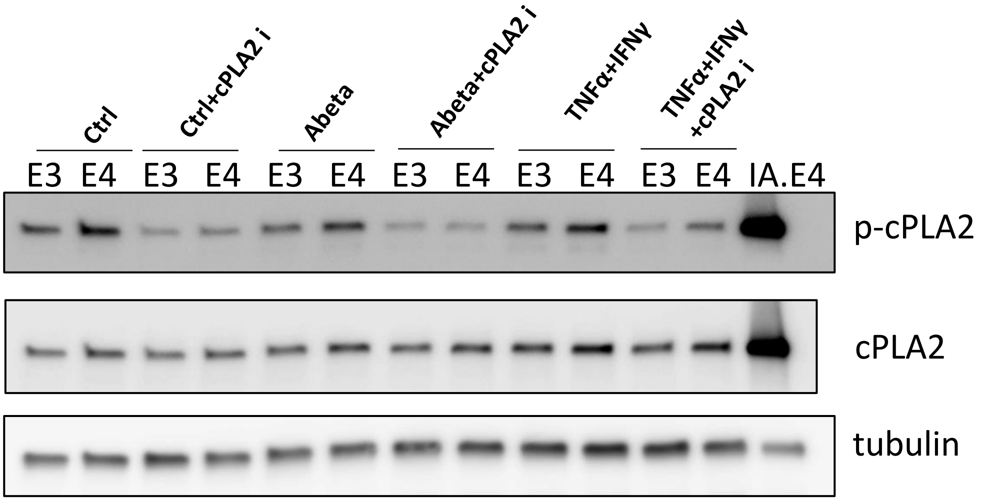

3rd

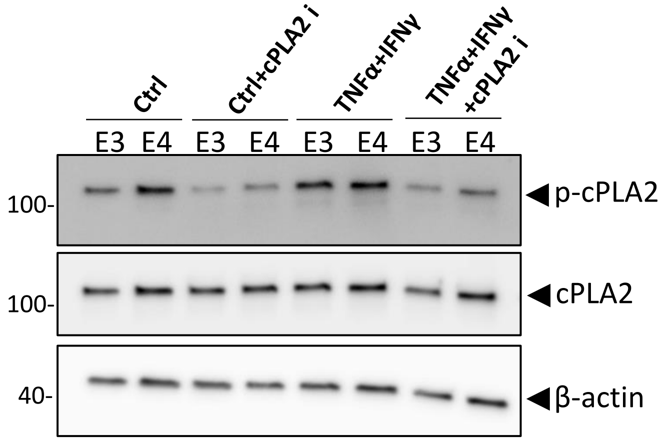

Figure 8A Repeats

Full blot 1st

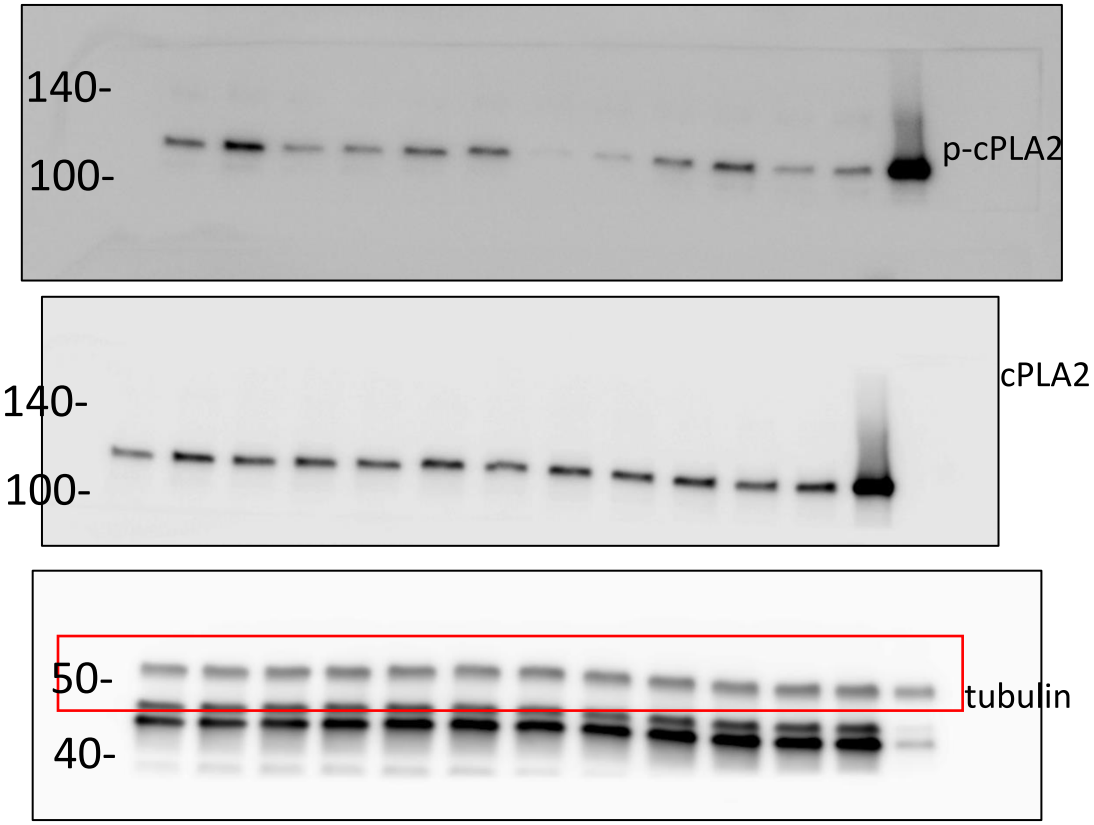

Figure 8A Repeats

Full blot 2nd

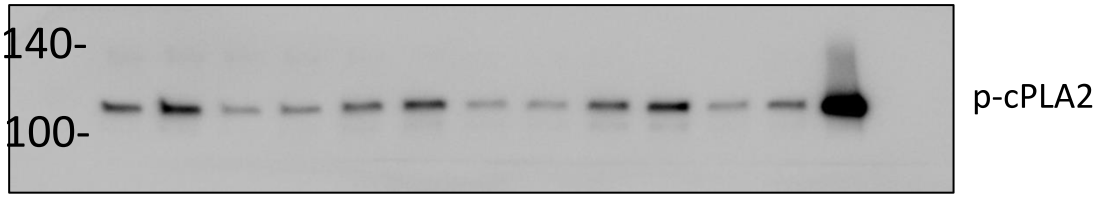

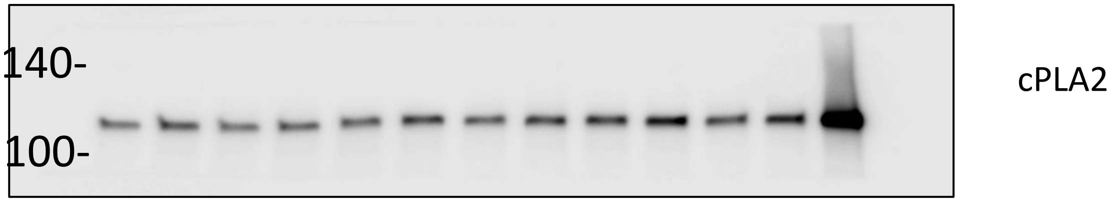

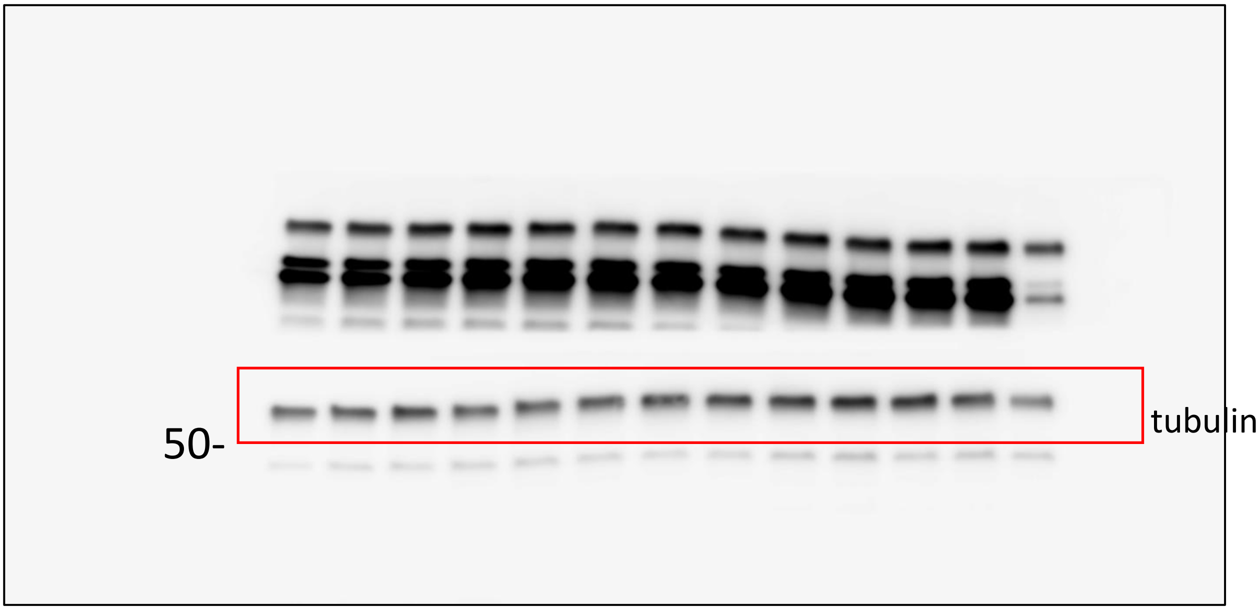

Figure 8A Repeats

Full blot 3rd

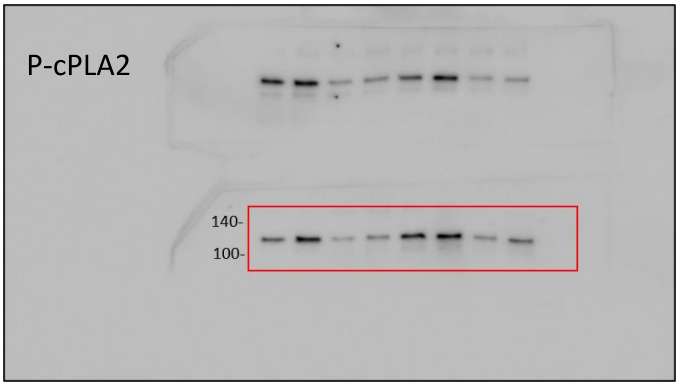

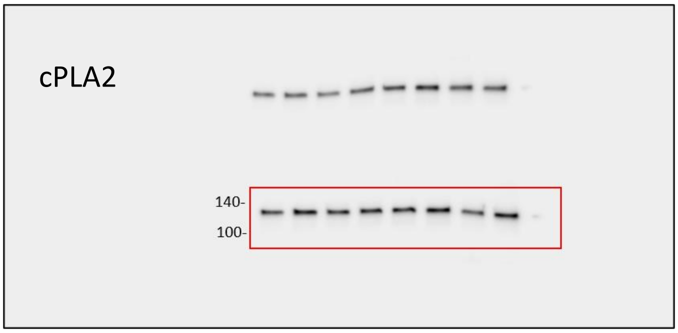

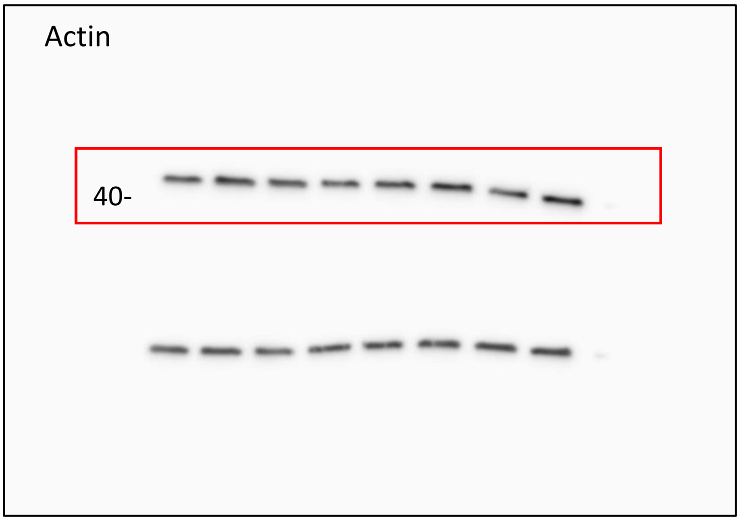

Figure 8C Repeats

1st

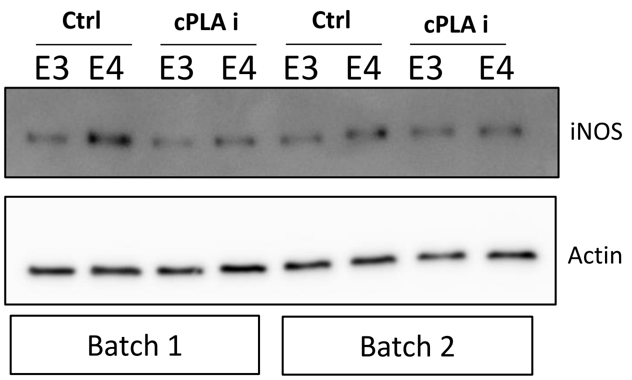

2nd

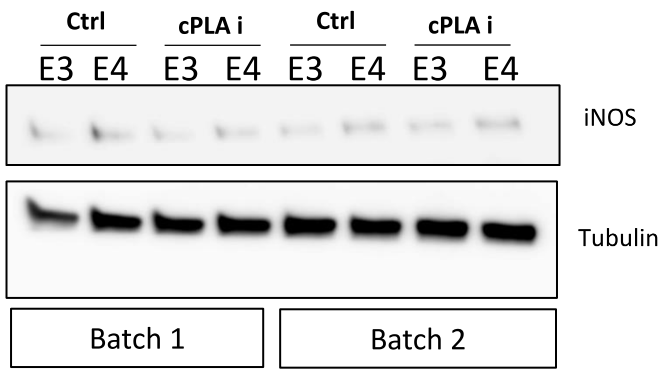

Figure 8C Repeats
Full blot 1st

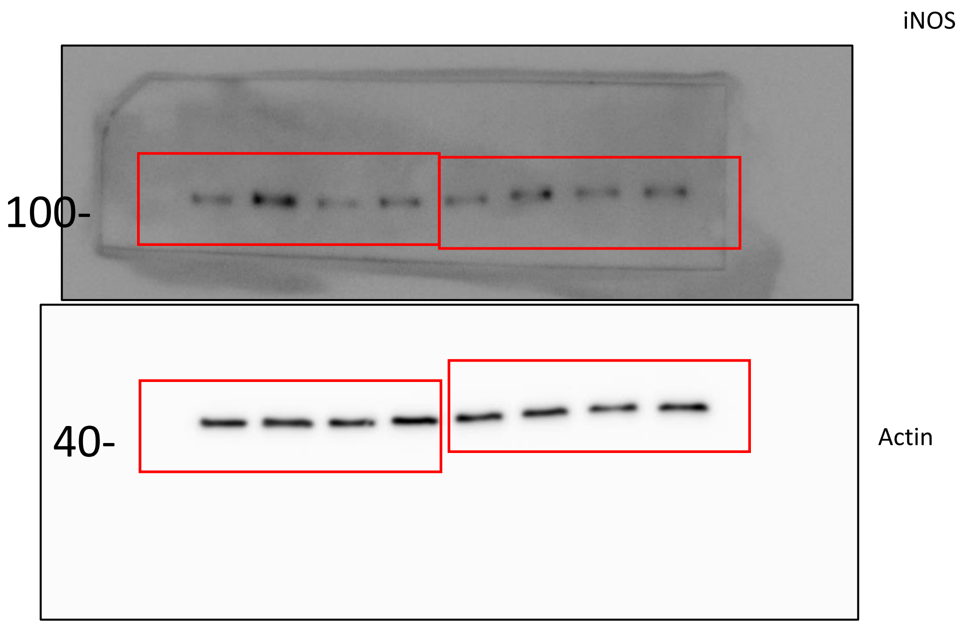

Full blot 2nd

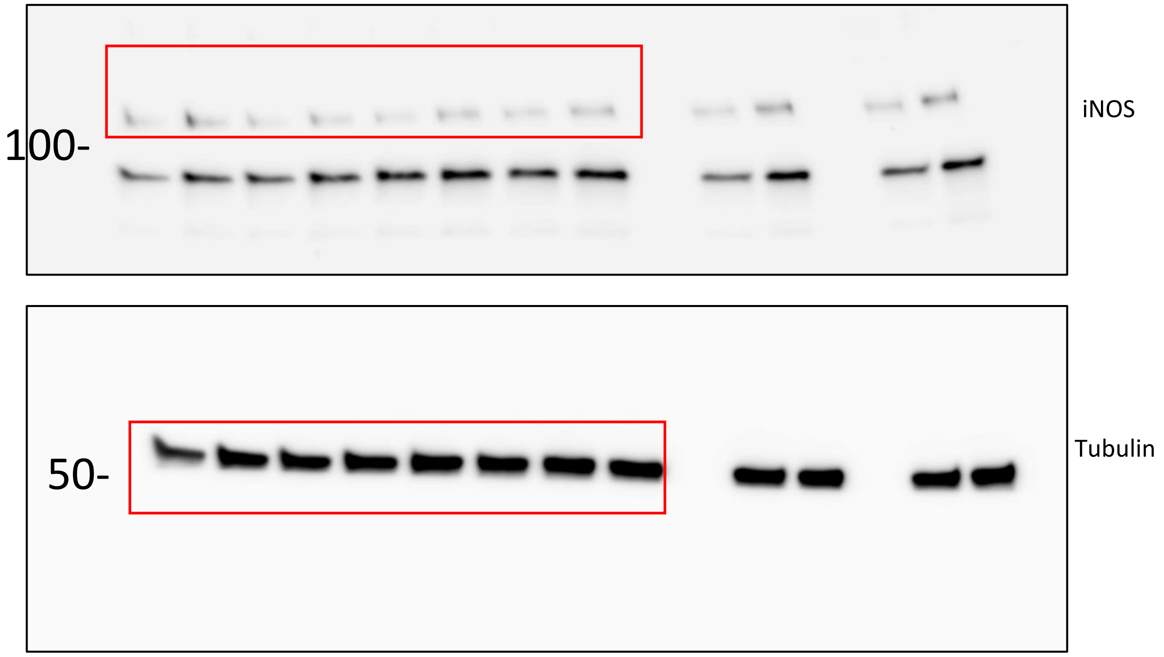

Fig. 8E

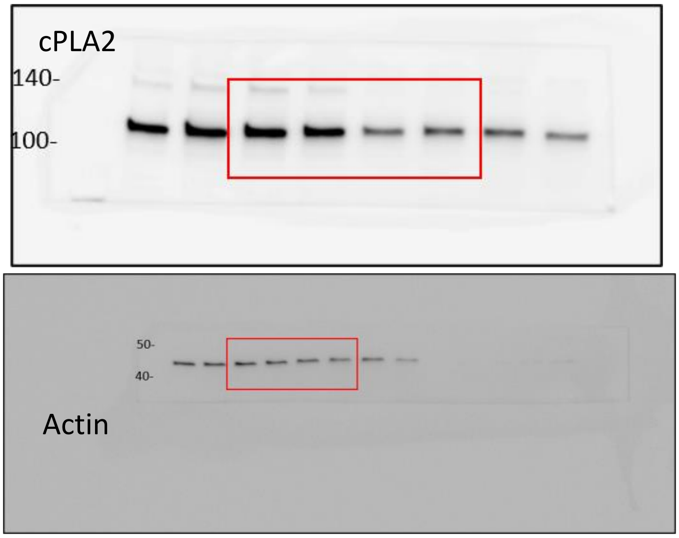

Figure 9 D repeats

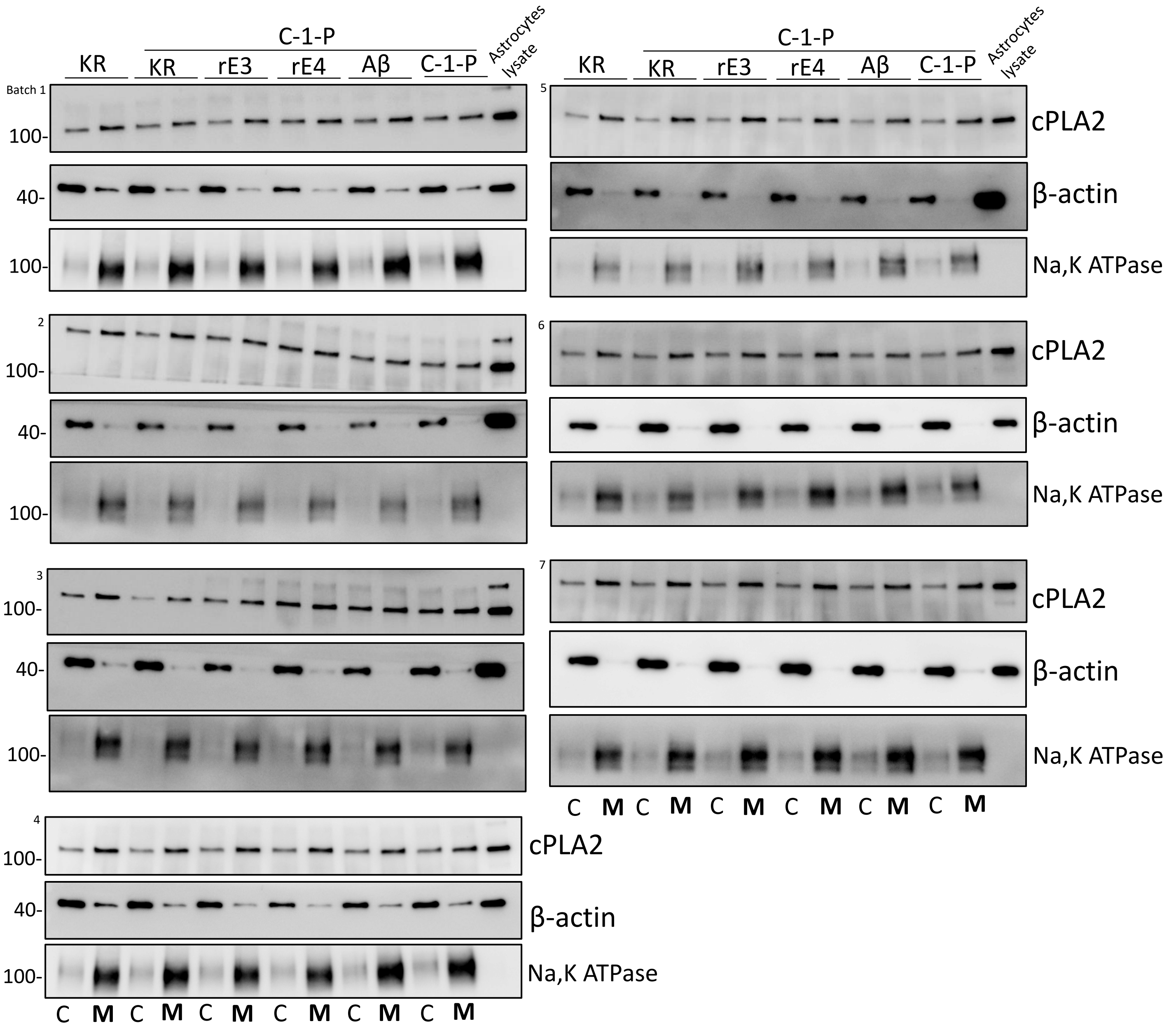

Figure 9 D repeats

Full blot batch 1

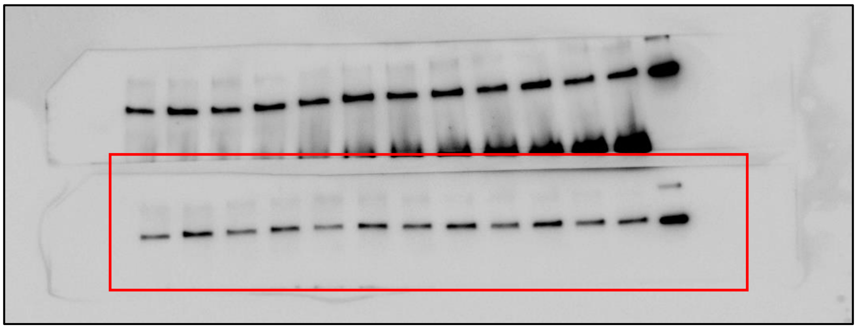

cPLA2

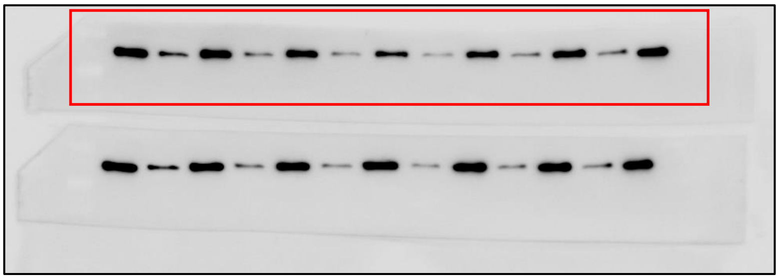

Actin

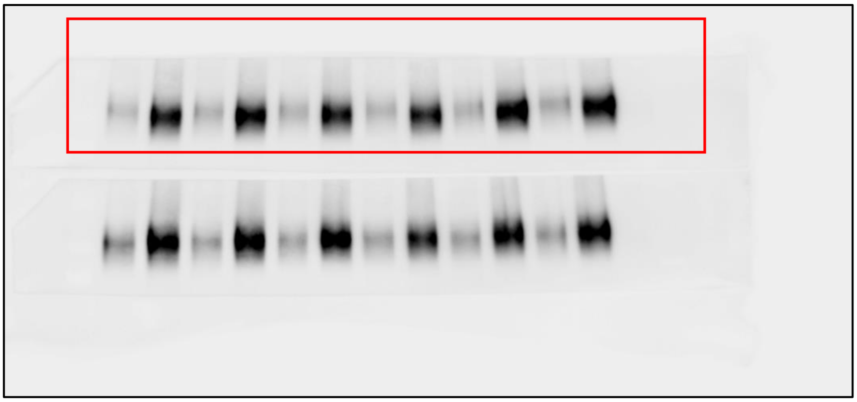

Na,K ATPase

Figure 9 D repeats

Full blot batch 2

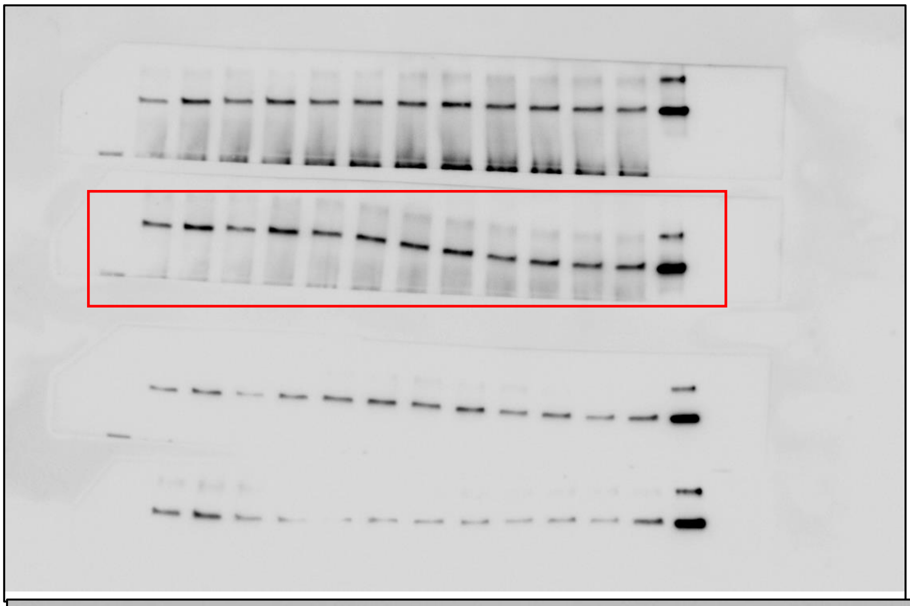

cPLA2

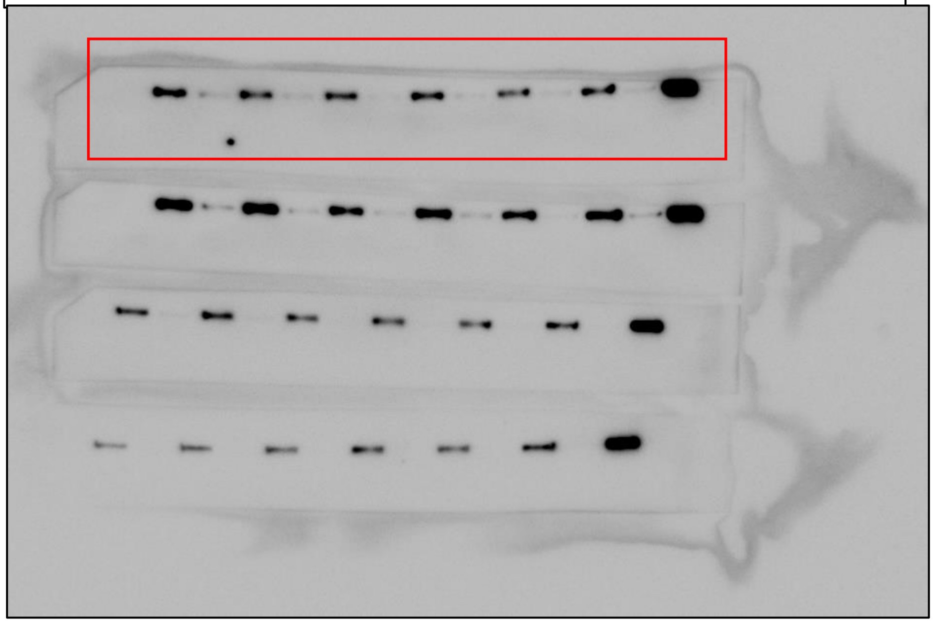

Actin

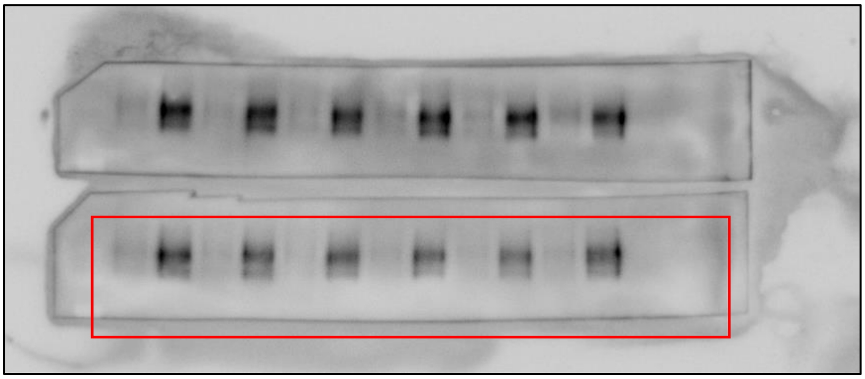

Na,K ATPase

Figure 9 D repeats

Full blot batch 3

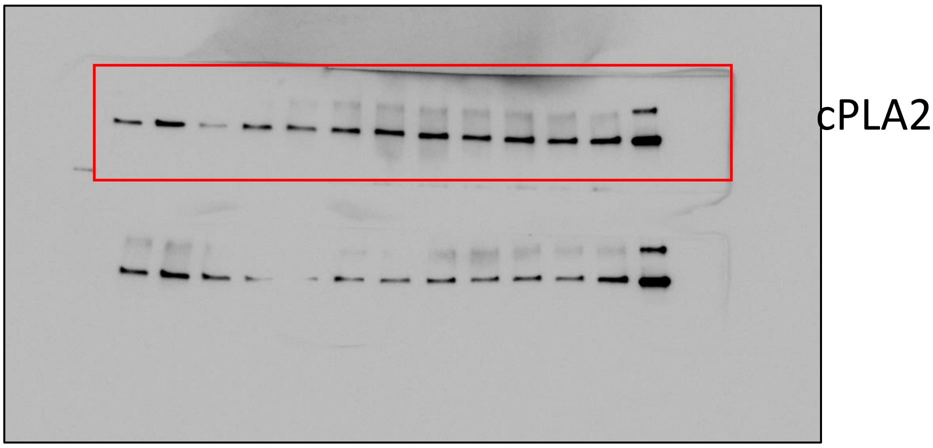

cPLA2

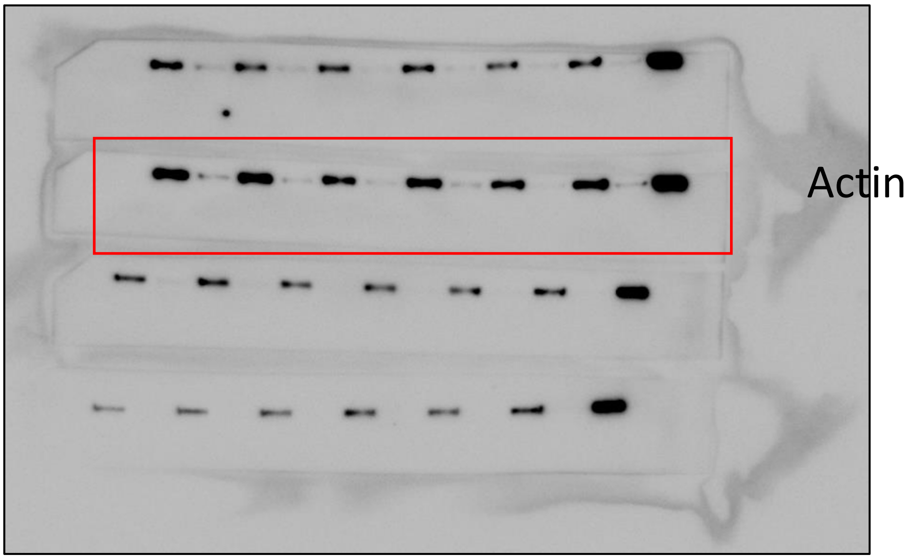

Actin

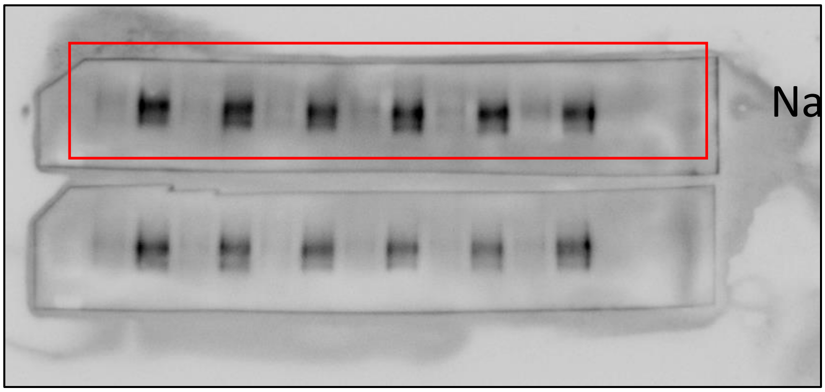

Na,K ATPase

Figure 9 D repeats

Full blot batch 4

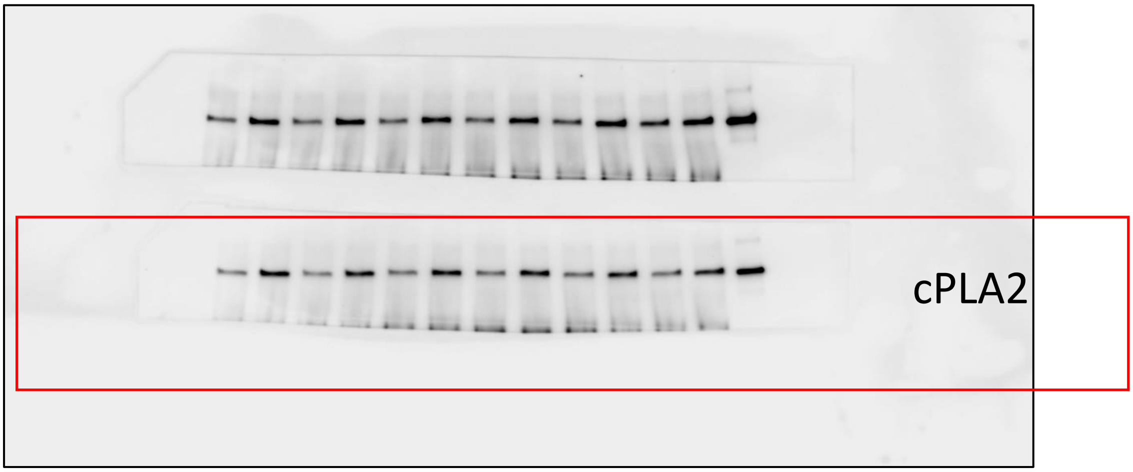

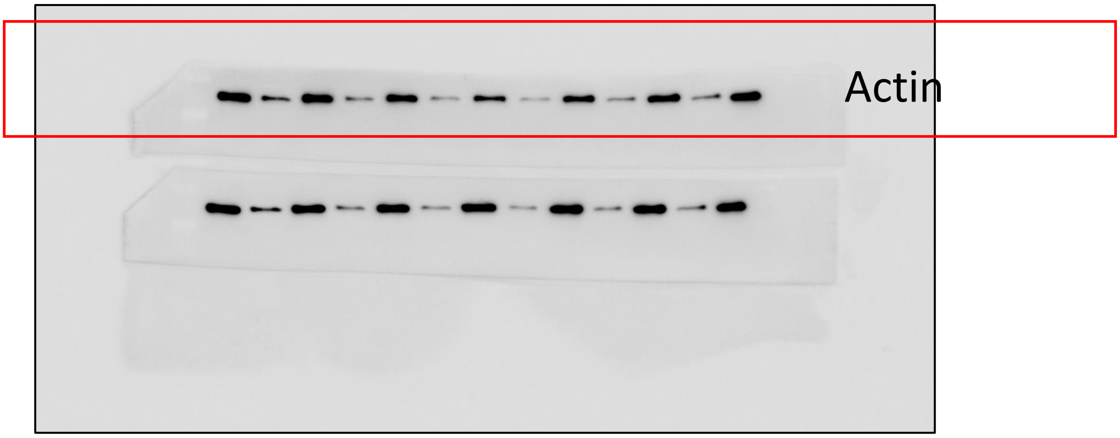

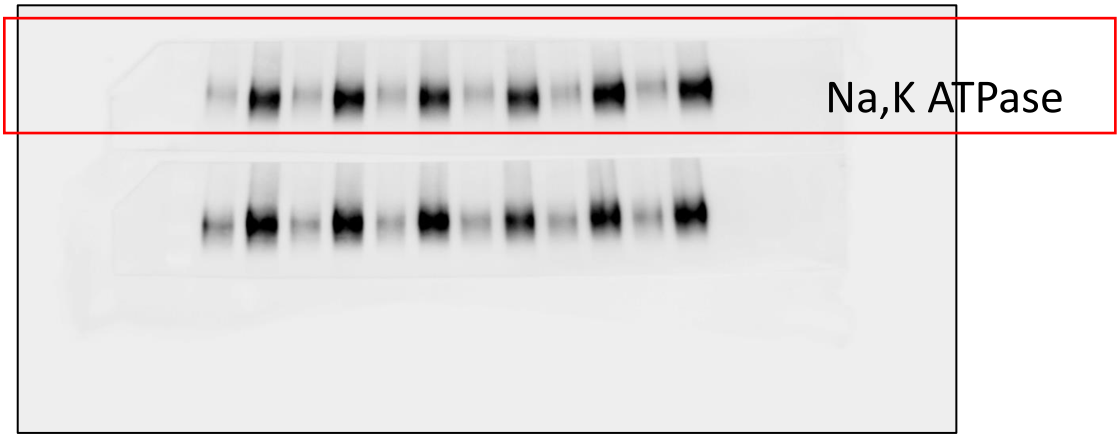

Figure 9 D repeats

Full blot batch 5

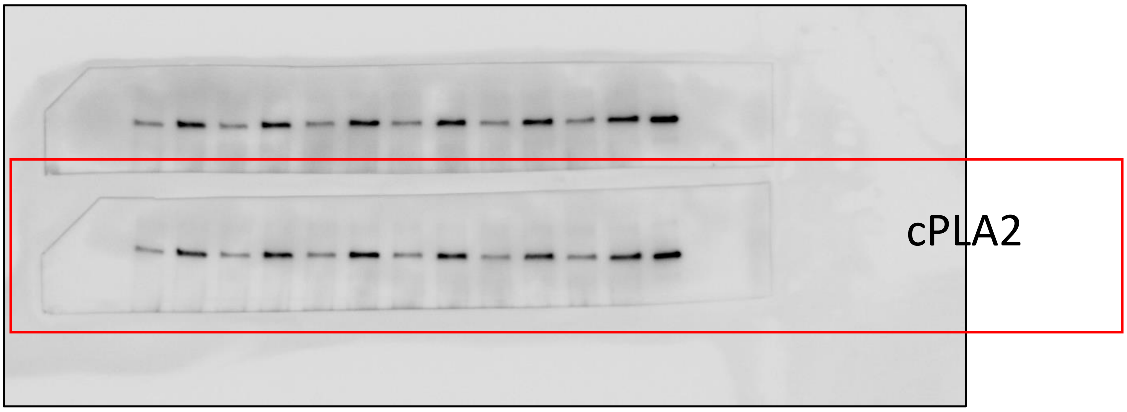

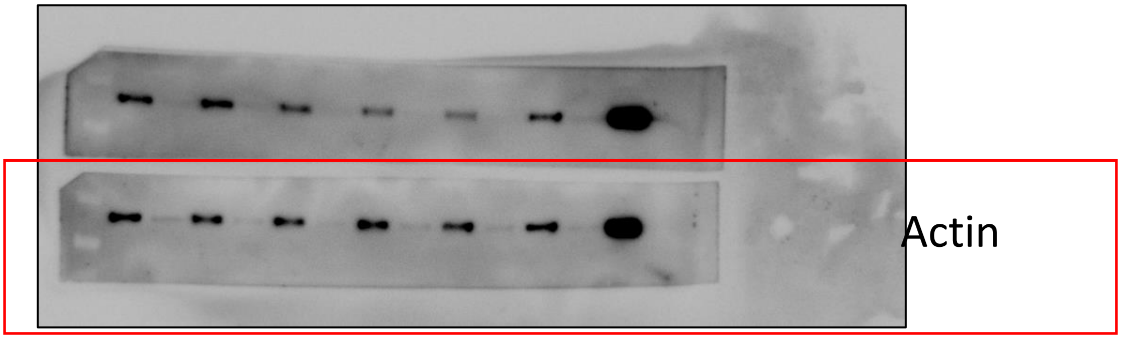

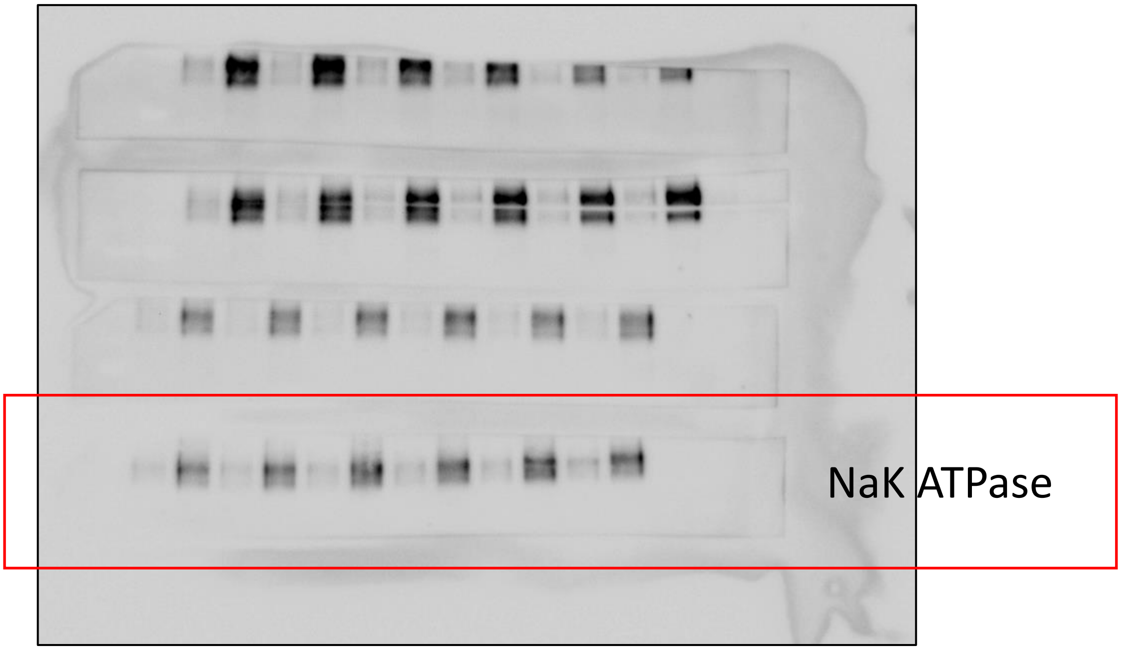

Figure 9 D repeats

Full blot batch 6

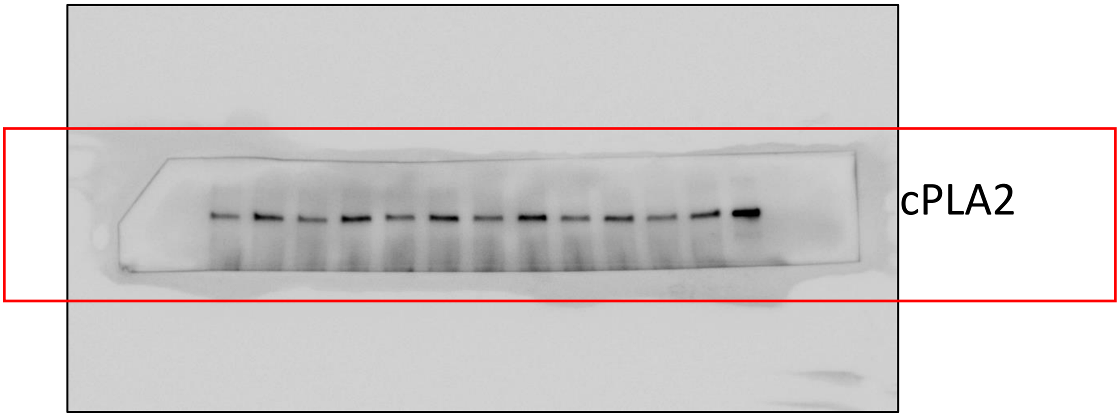

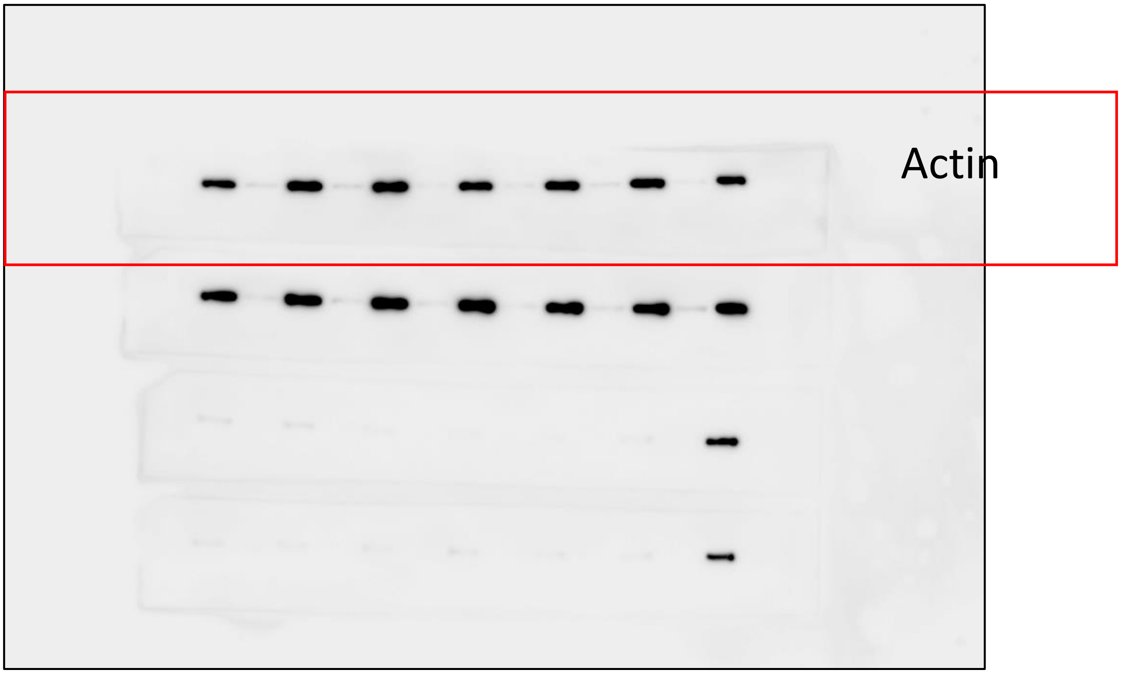

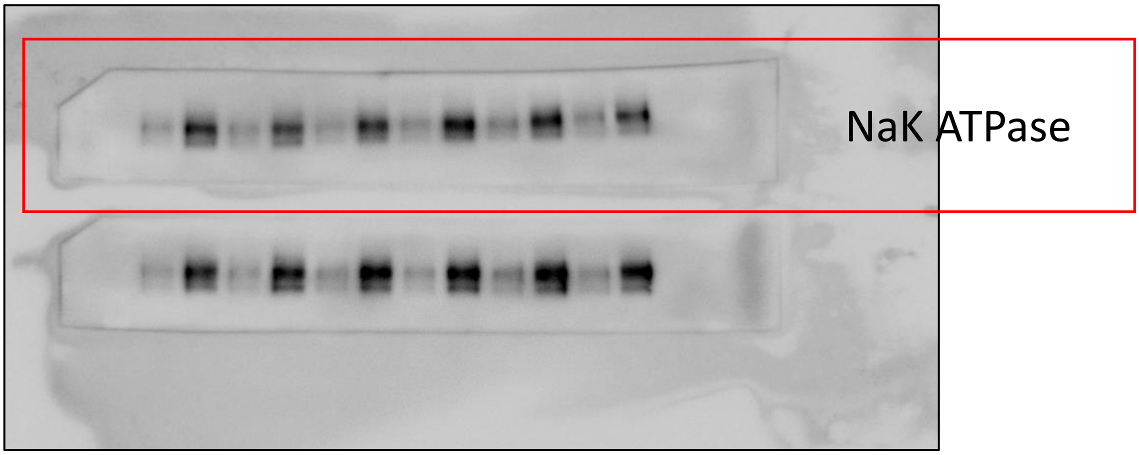

Figure 9 D repeats

Full blot batch 7

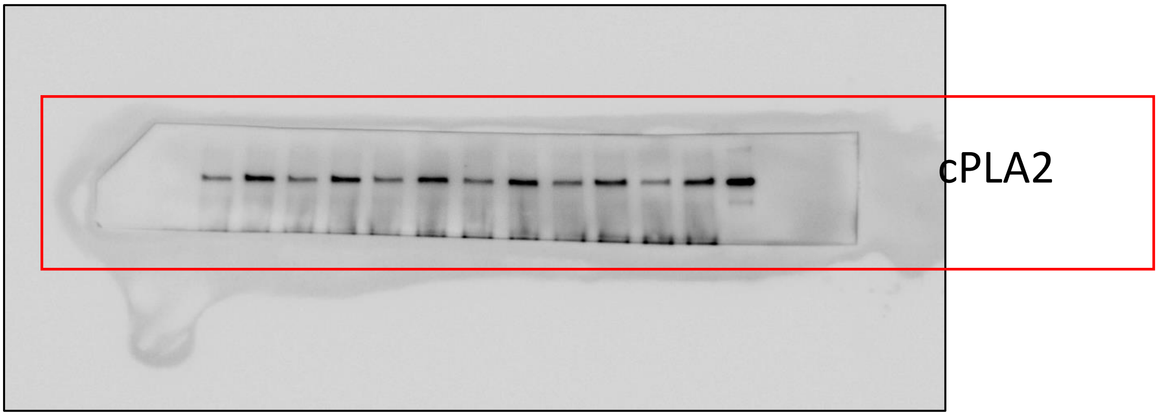

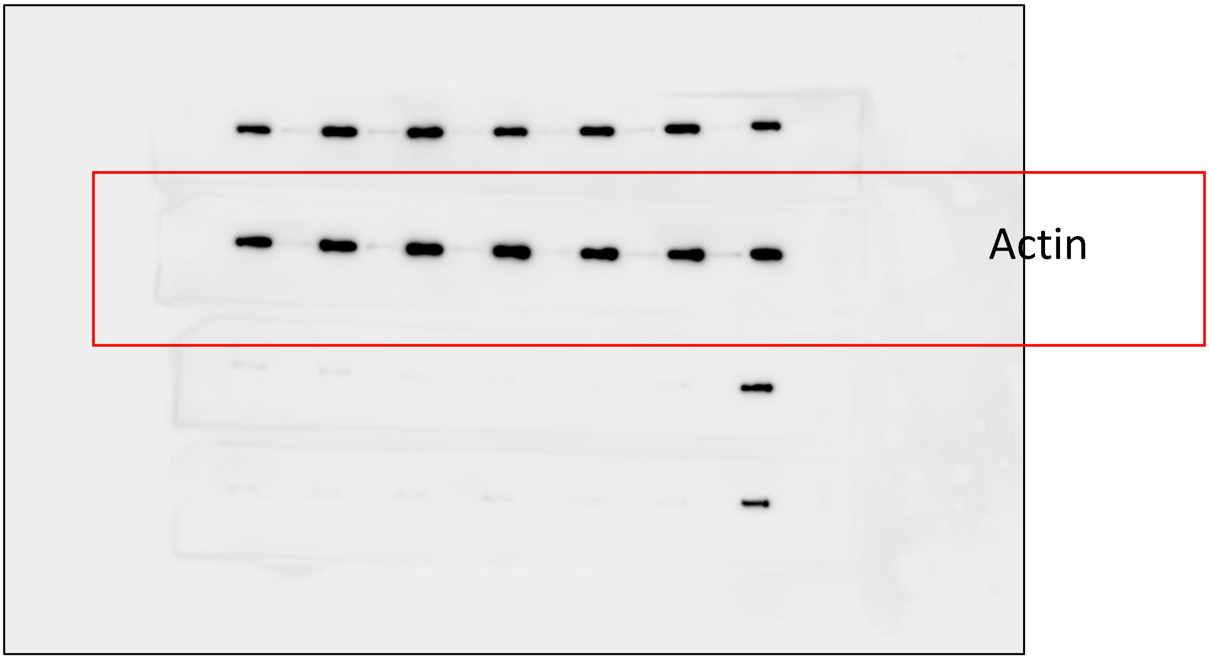

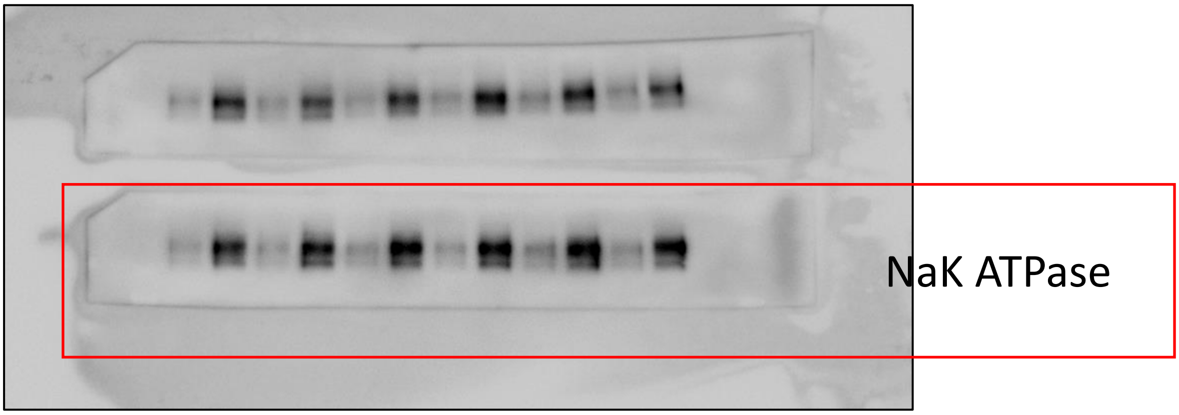

Fig. S2B

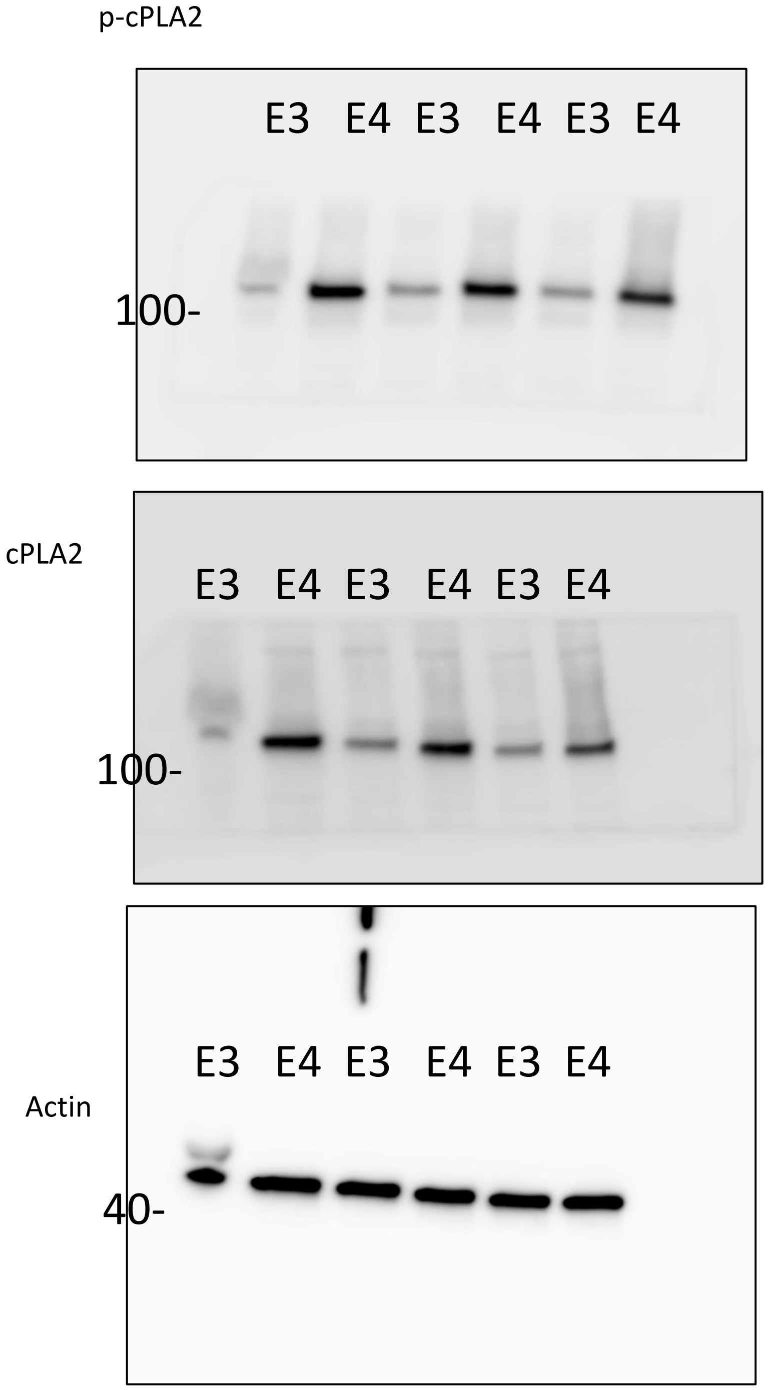

p-cPLA2
E3
E4
E3
E4
E3
E4
100-
cPLA2
E3
E4
E3
E4
E3
E4
100-
E3
E4
E3
E4
E3
E4
Actin
40-

Fig. S2C

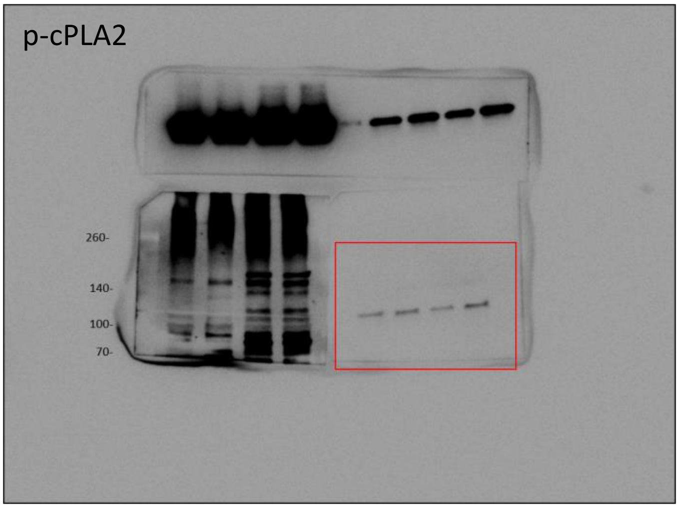

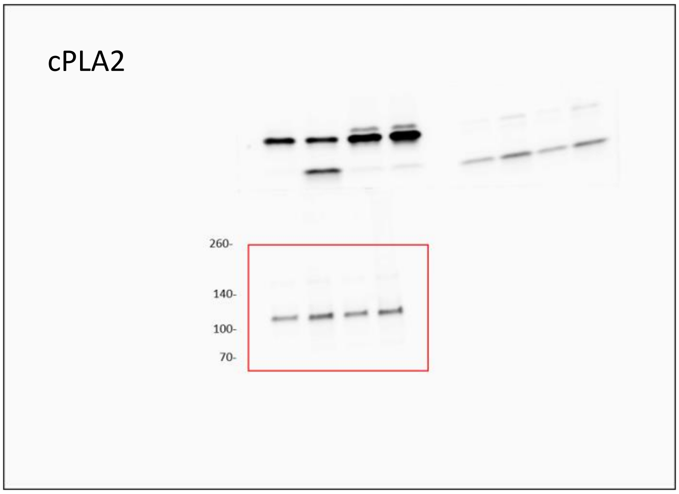

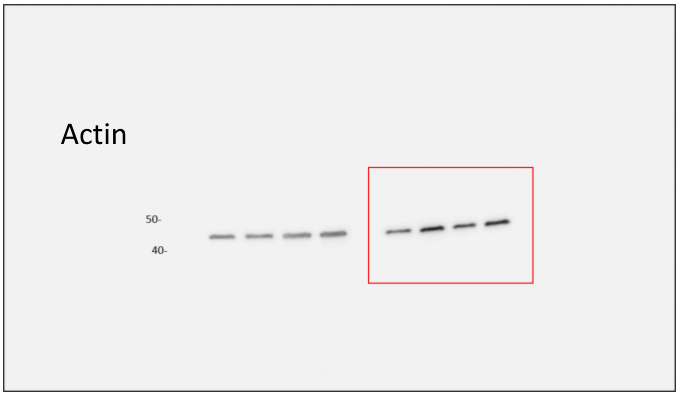

Fig. S3A

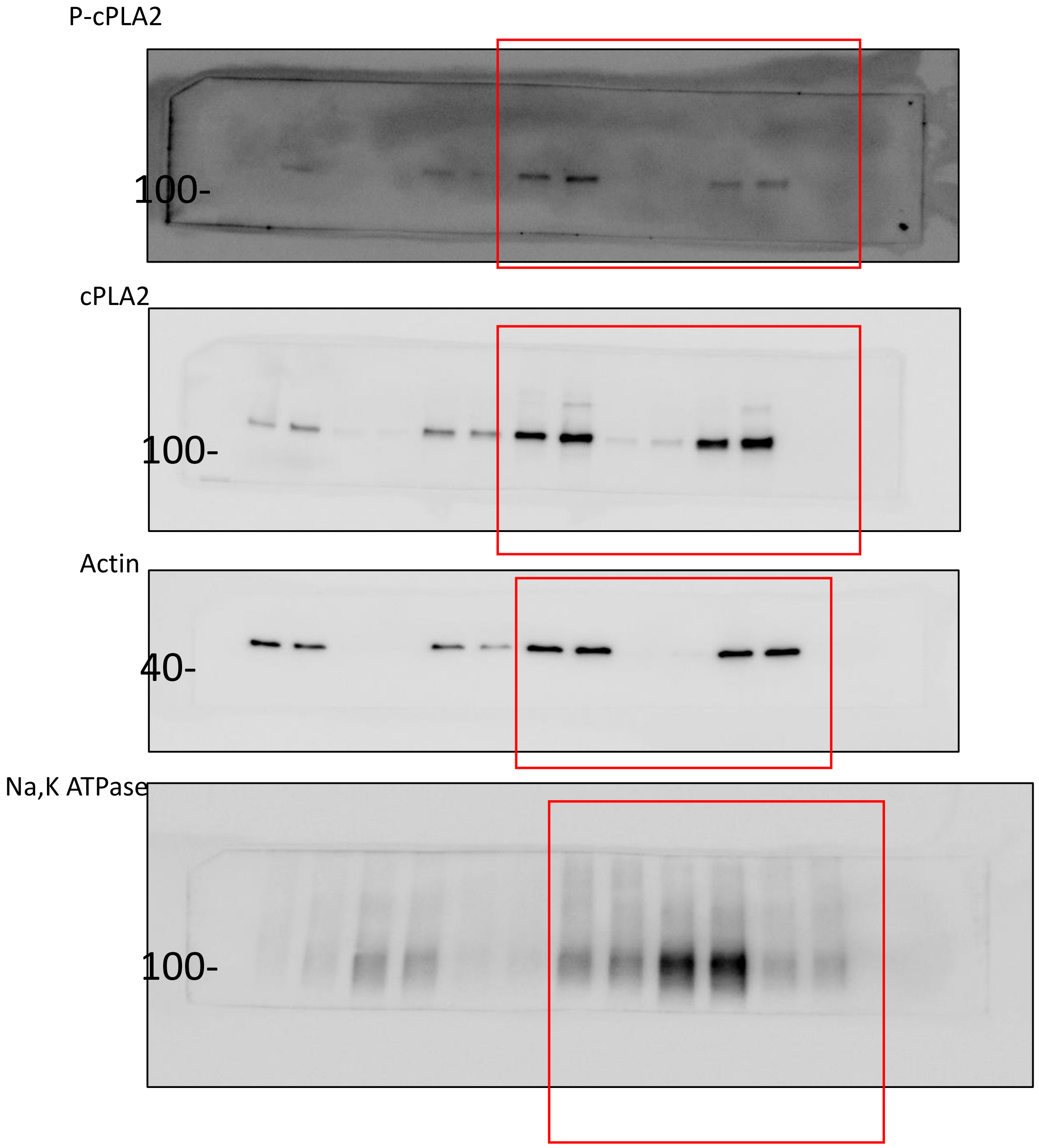

Fig. S4

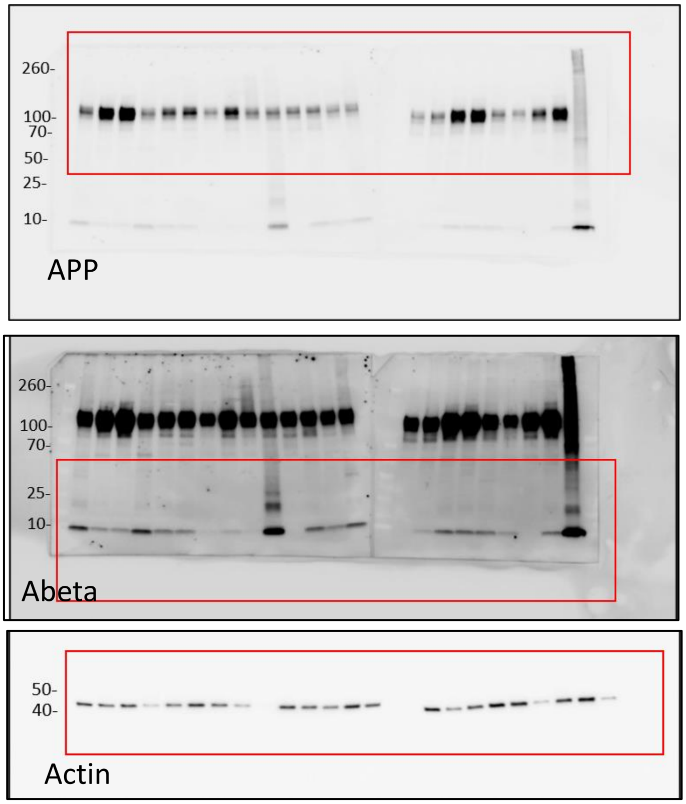

Fig. S5A

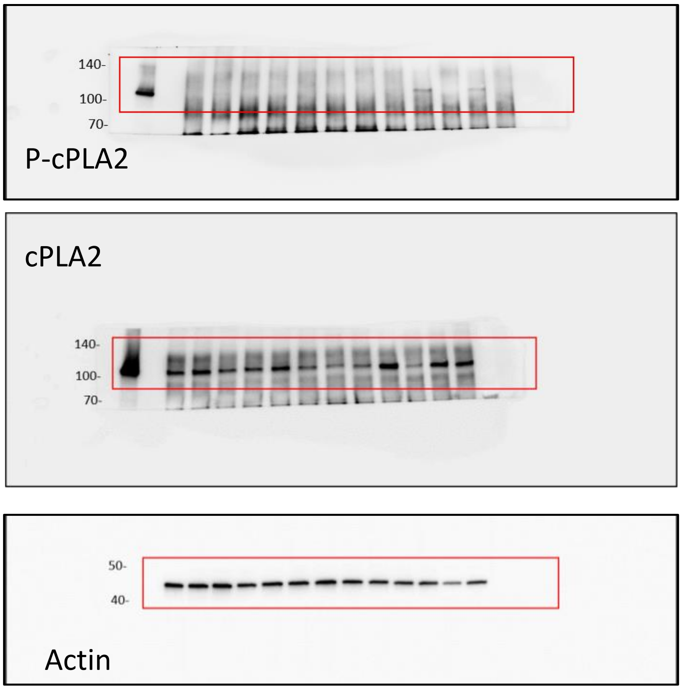

Fig. S5B

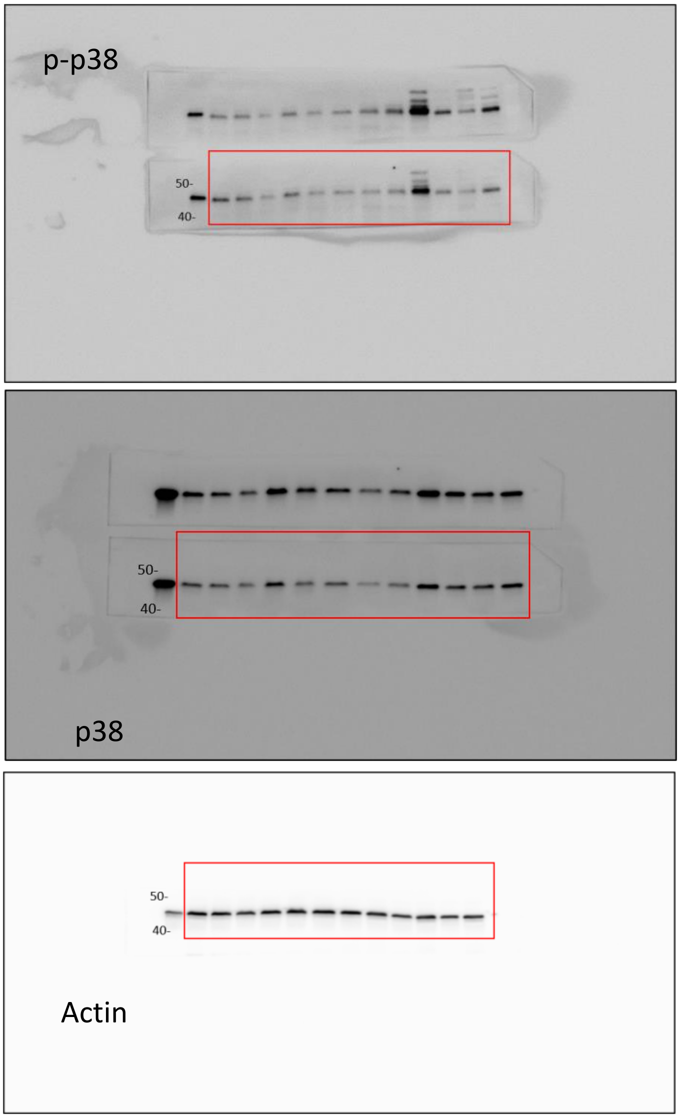

Supplement: Supplementary file 8 — Additional file 8. [file 13024_2022_549_MOESM8_ESM.pdf]
